# Advanced feature engineering in Acute:Chronic Workload Ratio (ACWR) calculation for injury forecasting in elite soccer

**DOI:** 10.1371/journal.pone.0327960

**Published:** 2025-07-23

**Authors:** Jaime B. Matas-Bustos, Antonio M. Mora-García, Moisés de Hoyo Lora, Alejandro Nieto-Alarcón, Francisco T. Gonzalez-Fernández

**Affiliations:** 1 Department of Signal Theory, Telematics and Communications, University of Granada, Granada, Spain; 2 Department of Physical Education and Sports, University of Sevilla, Sevilla, Spain; 3 Escuela Técnica Superior de Ingeniería Informática y Telecomunicaciones (ETSIIT), University of Granada, Granada, Spain; 4 Department of Physical Education and Sports, University of Granada, Granada, Spain; Portugal Football School, Portuguese Football Federation, PORTUGAL

## Abstract

Controlling training monotony and monitoring external workload using the Acute:Chronic Workload Ratio (ACWR) is a common practice among elite soccer teams to prevent non-contact injuries. However, recent research has questioned whether ACWR offers sufficient predictive power for injury prevention in elite competition settings. In this paper, we propose a novel feature engineering framework for training load management, inspired by bilinear modeling and signal processing principles. Our method represents external workload variables, derived from GPS data, as discrete time series, which are then integrated into a temporal matrix termed the Footballer Workload Footprint (FWF). We introduce calculus-based techniques—applying integral and differential operations—to derive two representations from the FWF matrix: a cumulative workload matrix ($\sum^{T}FWF$) generalizing Acute Workload (AW), and a temporal variation matrix ($\Delta^{T}FWF$) generalizing Chronic Workload (CW) and formulating the ACWR. Our approach makes traditional workload metrics suitable for modern machine learning. Using real-world data from an elite soccer team competing in LaLiga (Spain’s top division) and UEFA tournaments, we conducted exploratory and confirmatory analyses comparing multivariate models trained on FWF-derived features against those using traditional ACWR calculations. The FWF-based models consistently outperformed baseline methods across key performance metrics—including the Area Under the ROC Curve (ROC-AUC), Precision-Recall AUC (PR-AUC), Geometric Mean (G-Mean), and Accuracy—while reducing Type I and Type II errors. Tested on temporally independent holdout data, our top model performed robustly across all metrics with 95% confidence intervals. Permutation tests revealed a significant association between FWF matrices and injury risk, supporting the empirical validity of our approach. Additionally, we introduce an interpretability framework based on heatmap visualizations of the FWF’s cumulative and temporal variations, enhancing explainability.

These findings indicate that our approach offers a robust, interpretable, and generalizable framework for sports science and medical professionals involved in injury prevention and training load monitoring.

## 1 Introduction

Injuries represent one of the most undesirable and unpredictable events that professional soccer players may experience over the course of a season. Soccer is a sport characterized by frequent physical contact; however, non-contact musculoskeletal injuries, such as hamstring strains and anterior cruciate ligament ruptures, remain common. Indeed, more than 90% of all muscle injuries and 51%–64% of joint and ligament injuries in soccer occur in non-contact situations [1], with injury rates being higher in matches than in training sessions [2].

In general, training load is strongly associated with injury and illness risk in athletes [3]. As a consequence, sports science and medicine professionals monitor training monotony (TM) [4] to prevent overtraining and to avoid acute spikes in loads [5]. These professionals extract variables from all workload events for each player during the whole team season employing Electronic Performance and Tracking Systems (EPTS), like Heart Rate Variability Monitors (HRVM) and Global Positioning Systems (GPS) [6]. Medical and sports science experts monitor the internal and external workload of players, measuring the *acute:chronic workload ratio* (ACWR) for a set of variables [7], in a specific time period; then, depending on a threshold, called “*sweet spot*”, the risk is evaluated [8].

This process has become an established and widely adopted practice in elite soccer teams to prevent non-contact injuries and minimize the risk [9]. Nonetheless, recent research indicates that the ACWR model should be discarded as a framework [10] and highlights the lack of clear evidence on the ability of ACWR and TM variations to predict non-contact injuries in elite soccer players, although there is an association with injury risk [11]. Also, the proposer of ACWR, Tim Gabett, argued that “*traditional calculations of ACWR are ‘mathematically coupled’, as the most recent week is included in the estimation of both the acute and chronic workloads.*” [12]. The main effect is a ‘spurious’ correlation [13] of approximately r=0.5, between acute and chronic loads [12], which makes it difficult to clarify the influence of the different variables on injury risk, using this calculation.

In response, recent literature has proposed monitoring training progression by combining acute and chronic measures through a variety of strategies. For example, grouping time windows to measure week-to-week changes [14], calculate ACWR with rolling averages (RA) [7], using uncoupled ACWR calculation [8,12], or employing exponentially weighted moving averages (EWMA) [15]. Despite the popularity of the EWMA method to calculate ACWR, which is considered more sensitive to variations in load over time [16], most researchers agree that future investigations need to focus on more reliable measures. This is considered essential to improve the predictive power and accuracy of injury risk estimation [17].

Should these methods be considered sufficient? Can analytical ACWR calculations truly predict injuries the day before a match, in the context of modern elite soccer? Artificial Intelligence/Machine Learning (AI/ML) has been used to identify athletes at high injury risk during sport participation, and that it may be helpful to identify injury risk factors [18], but also with several limitations. Given that the core data of our research is derived from GPS technology, we conducted an exhaustive review of the literature, identifying four key challenges in using GPS-derived data to predict injury risk in soccer players from an AI/ML perspective, namely:

- Firstly, from an AI/ML perspective, monitoring training monotony (TM) using GPS data entails solving a data modeling and preprocessing challenge. How the raw data are cleaned, organized, and sampled for each player directly affects ML performance [19], and consequently, the accuracy of predictions and injury risk evaluations. The research, highlighted in the systematic review titled “*Machine Learning for Understanding and Predicting Injuries in Football*” [20] published in June 2022, demonstrates the potential of machine learning for bringing new insights to our understanding of injury prediction in soccer. This review highlights the considerable variability in study design and analysis and identifies as a key limitation the fact that most studies are based on data from a single season. It emphasizes the need to test and refine models with data from subsequent seasons, incorporating changes in players, coaches, training, and match conditions. Reviewing the state of the art, the authors have not been able to identify a standardized model describing how data should be collected and analyzed, beyond the calculation of the ACWR and the comparison with the “*sweet spot*” [8]. We observed that each team staff currently uses its own procedures to handle and process data, suggesting that the process remains informal in terms of data governance [21] and lacks a standardized data quality framework [22].
- Secondly, ACWR—whether calculated using the ‘${\text{ACWR}}_{\text{COUPLED}}$’ [7], ‘${\text{ACWR}}_{\text{UNCOUPLED}}$’ [8,12], or ‘${\text{ACWR}}_{\text{EWMA}}$’ [15,16]—along with other approaches such as the robust exponential decreasing index (REDI) [23], can all be framed within the AI/ML field of feature engineering [24]. However, none of these methods have been specifically designed to reflect how data-driven intelligent models operate when making predictions. Feature engineering is a crucial step in the ML pipeline, as selecting the right features can reduce modeling complexity and lead to better results [25].Accurate feature definition and selection [26] are also essential for addressing the correlation–causation dilemma [27] in complex multivariate problems such as injury prediction.
- Thirdly, while some studies associate ACWR peaks with increased injury risk [9], a mathematical analysis reveals that similar “*sweet spot*” values can result from very different training patterns. Hence, it is evident that the calculation of the ACWR, when used as a standalone threshold, is insufficient to depict the distribution of training over time, as well as to serve as a conclusive measure for predicting the risk of injury. Instead of debating the validity of ACWR as a predictor, it may be more appropriate to shift the focus toward the allocation and management of training load over time, which plays a critical role in either mitigating or increasing injury risk.
- Fourthly, to the best of our knowledge, the most cited study on GPS-based injury prediction in soccer is that of Rossi et al. [28]. Although their work was pioneering, injury prediction remains a major challenge in AI/ML due to the pronounced imbalance between injured and non-injured cases. The imbalanced learning issue [29], named so by data science researchers, is concerned with the performance of learning algorithms in the presence of underrepresented patterns and severe class distribution skews [30]. This requires dedicated techniques to transform large quantities of raw data into usable knowledge representations [31]. In practice, it also demands the use of appropriate algorithms [32], evaluation metrics [33], and performance analyses [34], all while managing trade-offs in model evaluation [35]. The choice of model must strike a balance between metric optimization [36], generalization capacity [37], and robustness against biased predictions and overfitting [38].

In this study, we introduce a novel approach to model and manage player training workloads, aiming to improve the prediction of non-contact injuries in elite soccer, particularly in the period leading up to matches. We present a new approach to control training load inspired by bilinear modeling [39] and the theoretical foundations of signal processing [40]. Our method represents each external workload variable extracted from GPS data as a discrete time series (DTS), which are joined together in a temporal discrete matrix that we call the Footballer Workload Footprint (FWF). We also define a method for computing the cumulative and temporary variations of the FWF matrix using integral and differential calculus, which we propose as a modernized version of ACWR adapted to the requirements of machine learning. In alignment with AI ethical principles [41], we additionally explore the explainability of the FWF model.

**Fig 1:**
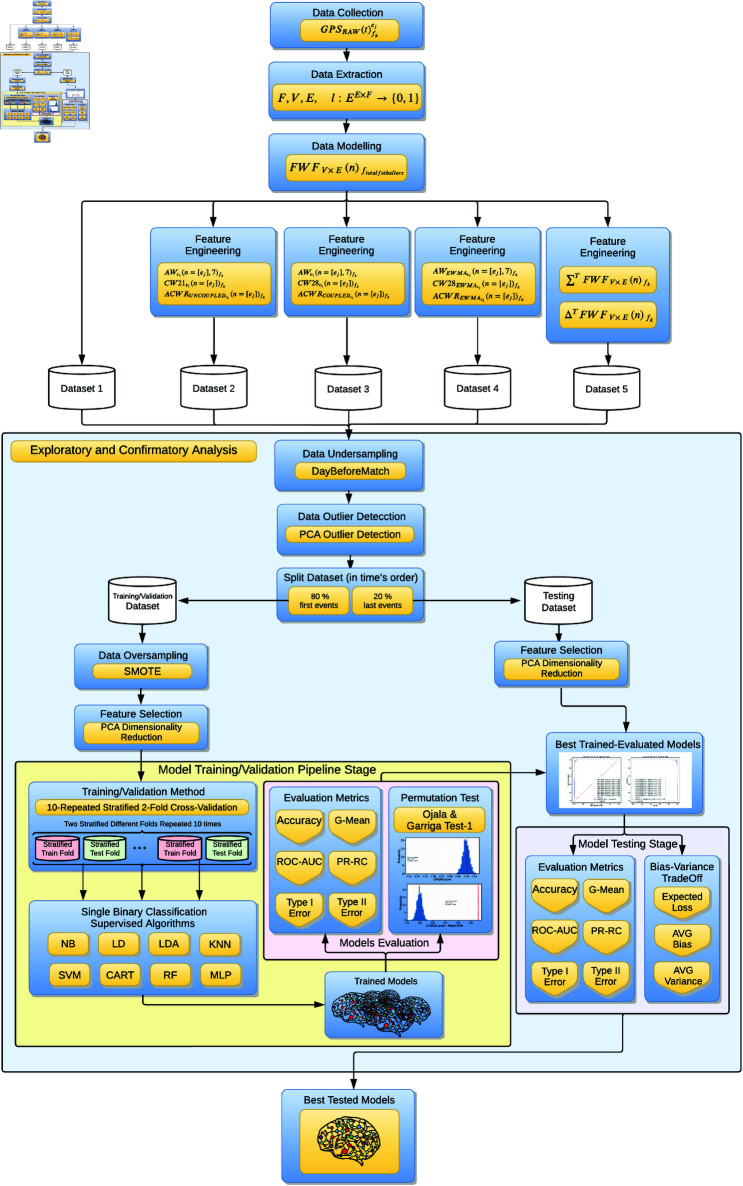
Experiment design and stages. The figure illustrates a summary of the different phases of our research. It outlines the sequential steps undertaken in our study to develop the datasets used later to perform the exploratory and the confirmatory analysis.

To assess our proposal, we compared the models trained using our novel approach with those trained using the most commonly referenced and utilized ACWR calculations by practitioners, specifically (‘${\text{ACWR}}_{\text{COUPLED}}$’ [7], ‘${\text{ACWR}}_{\text{UNCOUPLED}}$’ [8,12], and ‘${\text{ACWR}}_{\text{EWMA}}$’ [15,16]). As illustrated in Fig 1, we created five distinct datasets from a full season of GPS data recorded by a Spanish First Division (LaLiga) elite team, which also competed in Copa del Rey and UEFA tournaments. The medical services also provided the injury reports collected by the medical staff, containing information related to every injury suffered by the players during the season. In order to comprehensively analyze the datasets, we conducted both an exploratory analysis [42] and a confirmatory analysis [43] to assess, validate, and compare the models. This approach ensures a consistent and well-grounded understanding of the data.

This study constitutes a first step toward evaluating the validity of the FWF method, using widely accepted supervised machine learning techniques to ensure fair and objective assessment of the proposed approach. The study will evaluate models using key performance metrics, such as accuracy, Type I and Type II errors, G-mean, also ROC/AUC and PR/RC curves. We expect our models to demonstrate strong predictive performance during testing on new data, particularly in distinguishing between injury and non-injury events, with balanced results across evaluation metrics. We will compare our results with the most cited studies in the field, to the best of our knowledge [20], including Rossi’s work [28], as well as studies by Colby et al. [44], Carey et al. [45], Vallance et al. [46], and Hecksteden et al. [47]. Furthermore, permutation tests will be conducted to confirm the statistical significance of our model’s predictions, and a bias-variance trade-off analysis will assess its ability to capture the underlying relationships between input and output variables.

This paper is structured in five sections, including this introduction. Sect 2 presents the methodology used in this study. It includes a description of participants, data collection and extraction process, and an analysis of the different ACWR methods used in elite soccer to model and control the training workload of players, their shortcomings and defects. It also explains the data modeling and preprocessing steps, and details our feature engineering approach using the FWF matrix and the computation of cumulative and temporary variations, as well as their explainability. In addition, the section details the design of the datasets used for the exploratory and confirmatory analysis, the multivariate methods applied, and the training, validation, and evaluation process on new data. It also describes the permutation tests conducted and the bias–variance trade-off analysis performed. Sect 3 presents the results obtained from the experiments performed. Sect 4 is devoted to the discussion of these results, where we further examine the implications of these results in the context of their applicability to actual elite soccer. Finally, Sect 5 summarizes the findings of this study, limitations and highlights potential directions for future research.

## 2 Materials and methods

The design and phases of our experiment can be reviewed in Fig 1. More details about each stage are explained in successive subsections.

### 2.1 Participants

A total of 23 professional soccer players voluntarily agreed to participate in the data collection for this study. The study was carried out according to the Declaration of Helsinki [48]. Participants gave their informed written consent to participate in the study. The athletes belonged to an elite Spanish Primera División (LaLiga) soccer team, which also competed in the Copa del Rey and in UEFA European competitions. All participant data were thoroughly anonymized before being made available to the research team for the development of this retrospective study. The anonymization process ensured that the data scientists involved could not identify individual players based on the GPS-derived variables.

Accordingly, throughout the study, we refer to each soccer player as ‘${\text{f}}_{\text{k}}$’, representing one member of the total set of players, as expressed in Eq (1):

$$F=\{f_{1},f_{2},...,f_{k},...,f_{total\;footballers}\}$$ (1)

### 2.2 Data collection and data extraction

The data used in this study were collected over the course of one full season, including both the preseason and the competitive period, from July to May. During this time, the team participated in two official domestic competitions—the Spanish league and the Spanish cup—as well as in a European tournament. The dataset was provided to the researchers in September 2023 to carry out the analyses described in this manuscript.

We analyzed a total of 4,124 samples (from both training sessions and matches) corresponding to 23 individual players. All official matches played were included in the analysis. Training data consisted exclusively of ‘on-pitch’ sessions scheduled by the coaching staff. Sessions such as individual training, recovery, and rehabilitation were excluded. When a player was injured, he was only re-included in the study after completing one full week of training with the team. Goalkeepers were excluded from the analysis.

We stand for each season event ‘${\text{e}}_{\text{j}}$’ as part of the total set of events collected (Eq (2)):

$$E=\{e_{1},e_{2},...,e_{j},...,e_{total\;events}\}$$ (2)

Player movement during training sessions and matches was recorded using the WIMU PRO portable GPS device (a hybrid GNSS/LPS unit equipped with 4 x 3D accelerometers up to 1000 Hz, 3 x gyroscopes up to 1000 Hz, a 3D magnetometer at 100 Hz, and a barometer at 120 kPa, among other sensors). The GPS device was positioned between the shoulder blades using a compression vest to reduce movement artifacts. Speed and distance were sampled at 20 Hz, a frequency previously shown to offer valid and reliable measurements of instantaneous velocity across different movement intensities [49].

To ensure optimal performance, GPS units were activated and placed outdoors 15 minutes before warm-up [50]. Each player consistently used the same unit during the season to avoid inter-device variability [51].

We denote a collection of RAW data coming from GPS sensors for a footballer ‘${\text{f}}_{\text{k}}$’ in an event ‘${\text{e}}_{\text{j}}$’ as follows (Eq (3)):

$$GPS_{RAW}[n{]}_{f_{k}}^{e_{j}}=(\begin{array}{c} sensor_{1}[n]\\ sensor_{2}[n]\\ \vdots \\ sensor_{s}[n] \end{array})\;,\;n=0,1,2,\ldots ,N-1$$ (3)

where ‘*n*’ denotes the discrete sample index corresponding to the 20Hz sampling rate (i.e., one sample every Δt=0.05 seconds).

The raw GPS data for each player ‘${\text{f}}_{\text{k}}$’ during event ‘${\text{e}}_{\text{j}}$’ were downloaded using the manufacturer’s software and processed to extract the corresponding external workload variables. We denote the set ‘*V*’ of external workload variables extracted in an event ‘${\text{e}}_{\text{j}}$’, sampling from ‘*s*’ sensors of ${\text{GPS}}_{\text{RAW}}{(n{)}_{f_{k}}}^{e_{j}}$ as follows (Eq (4)):

$$V=\overset{s}{\underset{k=1}{⋃}}V^{\left(k\right)},\;\text{and}\;V^{\left(k\right)}=\{v_{1}^{\left(k\right)},v_{2}^{\left(k\right)},\ldots ,v_{m_{k}}^{\left(k\right)}\}$$ (4)

where:

(i) ${\text{V}}^{\left(\text{k}\right)}$: Variables derived from the *k*-th sensor (k∈{1,2,…,s}).
(ii) *m*_*k*_: Number of variables extracted from sensor *k*.
(iii) ⋃: Union operator indicating all variables across sensors.

For this study, we extracted for every event ‘${\text{e}}_{\text{j}}$’ considered and for each footballer ‘${\text{f}}_{\text{k}}$’ the same set ‘*V*’ of external workload variables. Table 1 shows the categories of all types of variables used.

**Table 1:** Categories of variables extracted from GPS sensors.

(1) Time Event Variables
(2) Distance Variables
(3) Speed Variables
(4) Acceleration and Deceleration Variables
(5) Sprints Variables
(6) Load Variables
(7) Power of Moving Variables
(8) Jumps, Step Balance and Impact Forces Variables
(9) Workload Variables Calculated from Expert Knowledge
(10) Intensity Variables Calculated from Expert Knowledge

Note: *the same category and workload variables were extracted for each footballer ‘${\text{f}}_{\text{k}}$’ in all events ‘${\text{e}}_{\text{j}}$’ considered.*

The set of workload variables ‘*V*’ was constructed by extracting external workload variables from the raw signals acquired by the multiple sensors embedded in the GPS units, including GNSS positioning, tri-axial accelerometry, gyroscopy, magnetometry, and barometric pressure sensors. The set includes metrics such as distance, speed, acceleration, deceleration, step balance, player load, jump count, and impact forces. It also includes variables that describe the distribution of speed and acceleration across defined ranges, as well as the percentage of time spent at different intensities. Additional metrics capture the duration and intensity of activity, power of movement, number of sprints, and time-event-related data. Some variables were directly measured (e.g., distance covered, instantaneous speed, linear acceleration), while others were derived through processing and expert domain knowledge (e.g., playerload, number of sprints, step balance, or jump counts). A complete list and description of the 73 variables ‘${\text{v}}_{\text{i}}$’ used in this study is provided in S1 Table.

The injury reports, provided by the club’s medical services, included complete information on all injuries sustained by players throughout the season and were fully anonymized. In total, 49 injuries were reported by the medical personnel and, in particular, 33 non-traumatic muscular injuries were recorded during the season. According to UEFA regulations, a non-contact injury is defined as any tissue damage that causes a player to be absent from physical activity for at least one day following the onset of symptoms.

Only injuries sustained by the 23 participants in this study were considered; all others were excluded from the analysis. In line with the objectives of our investigation and following recommendations from previous studies [52], we focused specifically on 23 muscle injuries, which were classified by the authors according to the UEFA muscle injury classification system [53] as grade I and grade II fiber ruptures. Contractures and minor overloads were excluded. A comprehensive list of these injuries is provided in Table 2.

**Table 2:** Injuries considered in the study.

Grade I Muscle-Fascial Strain
Left Quadriceps Vastus Internus
Right Biceps Femoris Distal
Left Obturator Externus
Left Psoas
Right Miotendinous Soleus
Left Miofascial Semitendinosus
Left Miofascial Biceps Femoris
Right Biceps Femoris
Right Miofascial Biceps Femoris

Grade II Muscle-Fascial Strain
Right Internal Gastrocnemius
Right Soleus
Left Soleus
Left Semitendinosus
Left Biceps Femoris
Right Biceps Femoris
Right Miotendinous Biceps Femoris
Right Adductor Longus

*Possible types of injuries sustained by a soccer player ‘${\text{f}}_{\text{k}}$’ during the events ‘${\text{e}}_{\text{j}}$’ under consideration.*

Leveraging the injury reports provided by the club, we have the capability to formulate the following linear application ‘*l*’ (Eq (5)):

$$l:E^{E\times F}\to \{0,1\},\;l(f_{k},e_{j})=\left\{ \begin{array}{cc} 1, & \text{if }f_{k}\text{ suffers an injury in event }e_{j}.\\ 0, & \text{if }f_{k}\text{ does not suffer an injury in event }e_{j}. \end{array}\right.$$ (5)

where, ‘${\text{f}}_{\text{k}}$’ ∈F and ‘${\text{e}}_{\text{j}}$’∈E, enabling us to query the occurrence of injuries in each soccer player ‘${\text{f}}_{\text{k}}$’, specifically within the context of event ‘${\text{e}}_{\text{j}}$’.

### 2.3 Data modeling and preprocessing: Introducing the Footballer Workload Footprint (FWF)

The first major contribution of this paper lies in the domain of data modeling [54]. The data model developed for this study is embedded within an advanced Extract-Transform-Load (ETL) process [55], designed to assist sports scientists and medical staff in managing the growing volume of data produced by tracking systems [56]. Their responsibilities are increasingly complex, involving not only decisions at the organizational level [57] but also detailed management of player performance and injury risk in top-tier soccer clubs [58].

The primary goal of the proposed model is to improve daily monitoring of training monotony (TM) and to optimize the tracking of both internal and external workload variables in elite soccer players. As previously described, this problem is framed across three dimensions: F (footballers, Eq (1)), V (variables, Eq (4)), and E (season events, Eq (2)).

**Fig 2:**
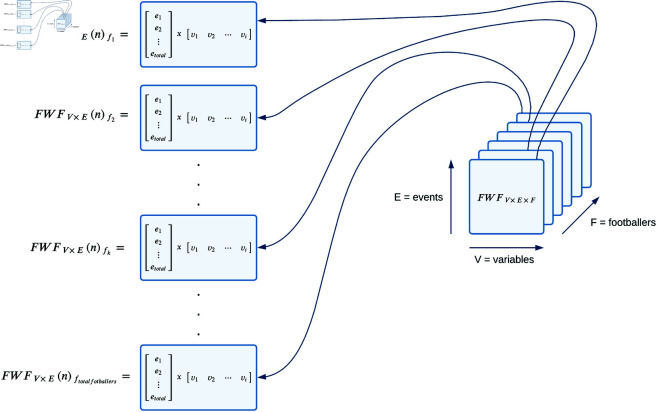
Bilinear modeling of batch process: variable-wise approximation, 3-way problem in a 2-way problem. Figure shows the method that transforms our 3-way problem F (footballers Eq (1)), V (variables Eq (4)), and E (season events Eq (2)) in a 2-way problem.

To handle this complexity, we represent the data following principles from data-centric process systems engineering [59]. Specifically, we model the data as if it originated from a process tracking batch system [60]. Based on techniques from bilinear model processing, we adopt a ‘*variable-wise*’ bilinear approximation for batch processes [39]. This method treats each sample collected at a specific time point as an individual object, thereby transforming the original three-way problem into a two-way problem, as illustrated in Fig 2.

Inspiring us also on theoretical bases of signal processing [40], our method represents each external workload variable ‘${\text{v}}_{\text{i}}$’ extracted from a GPS unit of a footballer ‘${\text{f}}_{\text{k}}$’ and during all the ‘*n*’ season events, as a Discrete Time Series (DTS) denoted as ‘$X_{v_{i}}(n)$’ Eq (6):

$$X_{{\text{v}}_{\text{i}}}(n)=[\begin{array}{cccc} x_{{\text{v}}_{\text{i}}}(e_{1}) & x_{{\text{v}}_{\text{i}}}(e_{2}) & \cdots & x_{{\text{v}}_{\text{i}}}(e_{\text{total}}) \end{array}]$$ (6)

Afterward, we joined together all ‘$X_{{\text{v}}_{\text{i}}}(n)$’ for a footballer ‘${\text{f}}_{\text{k}}$’ in a temporal discrete matrix denoted as Eq (7):

$$FWF_{V\times \;E}\;(n{)}_{f_{k}}=(\begin{array}{c} X_{v_{1}}(n)\\ X_{v_{2}}(n)\\ \vdots \\ X_{v_{i}}(n) \end{array})={\left(\begin{array}{cccc} x_{v_{1}}(e_{1}) & x_{v_{1}}(e_{2}) & \cdots & x_{v_{1}}(e_{j})\\ x_{v_{2}}(e_{1}) & x_{v_{2}}(e_{2}) & \cdots & x_{v_{2}}(e_{j})\\ \vdots & \vdots & \ddots & \vdots \\ x_{v_{i}}(e_{2}) & x_{v_{i}}(e_{2}) & \cdots & x_{v_{i}}(e_{j}) \end{array}\right)}_{\#(V)\;\times \;\#(E)}$$ (7)

where:

(i) $f_{\text{k}}\in F$, $F=\{{\text{f}}_{1},{\text{f}}_{2},...,{\text{f}}_{\text{k}},...,{\text{f}}_{\text{totalfootballers}}\}$ (Eq (1)).
(ii) n∈E, $E=\{{\text{e}}_{1},{\text{e}}_{2},...,{\text{e}}_{\text{j}}\}$ is a subset of season events considered from the total set of events (Eq (2).
(iii) $v_{\text{i}}\in V$, $V=\{{\text{v}}_{1},{\text{v}}_{2},...,{\text{v}}_{\text{i}}\}$ is a subset of variables considered from the total set of external workload variables sampled and extracted (Eq (4) from ‘*k*’ sensors of ${\text{GPS}}_{\text{RAW}}(\text{n}{)}_{\text{f}}^{e_{\text{j}}}$ (Eq (3)).
(iv) #(V) = total ‘${\text{v}}_{i}$’ variables considered.
(v) #(E) = total ‘${\text{e}}_{\text{j}}$’ events considered.

We named this temporal discrete matrix (Eq (7)) as Footballer Workload Footprint (FWF) or “*footprint*”, because it represents the external workload of the footballer as a “*footprint in the time*”. The proposed model is flexible and configurable, since it let us to determine the number of events and workload variables, and it allows to explore and focus the data in the most convenient temporal window required for any specific analysis.

Also, a compatible structure is necessary for managing injuries in accordance with ‘*FWF*’. We denote the matrix of injuries of a footballer ‘${\text{f}}_{\text{k}}$’ during all the ‘*n*’ season events, as a discrete time series (DTS) denoted as ‘$L_{f_{k}}(n)$’ Eq (8):

$$L_{E\times \;1}\;(n{)}_{f_{k}}={\left[\begin{array}{ccc} l(e_{1},f_{k}) & l(e_{2},f_{k})\cdots & l(e_{j},f_{k}) \end{array}\right]}_{\#(E)\;\times \;1}$$ (8)

where:

(i) $f_{\text{k}}\in F$, $F=\{{\text{f}}_{1},{\text{f}}_{2},...,{\text{f}}_{\text{k}},...,{\text{f}}_{\text{totalfootballers}}\}$ (Eq (1)).
(ii) n∈E, $E=\{{\text{e}}_{1},{\text{e}}_{2},...,{\text{e}}_{\text{j}}\}$ is a subset of season events considered from the total set of events (Eq (2).
(iii) *l* is the linear application $l:E^{E\times F}\to \{0,1\}$, defined in (Eq (5)).
iv #(E) = total ‘${\text{e}}_{\text{j}}$’ events considered.

This last structure complements the ‘*footprint*’, concluding our data modeling process and allowing us to manage information with agility in order to design and build the different datasets used in our experiments.

Beyond supporting our experimental framework, the Footballer Workload Footprint (FWF) also facilitates the standardized sharing of precise digital information about a footballer over a specific time span. We believe this approach could be seriously considered as a future industry standard by sports scientists and medical staff. It promotes collaboration with the data science community in studies involving workload datasets, as it provides a “*common language*” for structuring and analyzing data.

The conceptual foundation of the footprint lies in its ability to extract, transform, and load heterogeneous data sources—such as internal and external workload, musculoskeletal and biochemical data, nutritional and wellness factors—into a unified FWF representation for a given player ‘${\text{f}}_{k}$’. This process enables normalization of data from diverse origins, allowing for coherent and consistent analysis while also opening new possibilities for time-variant modeling of training loads. In addition, the method allows researchers to reinterpret datasets from previous studies and convert them into a FWF-based format—“*footprints datasets*”—suitable for comparative and longitudinal analysis.

It also supports the integration of data from multiple teams, measurement systems, or contexts with limited injury records, enabling the construction of larger, more generalizable databases. This is particularly relevant in a domain where data collection protocols often differ, for example, due to variations in GPS hardware. Moreover, existing datasets tend to be highly imbalanced, with very few injury cases—a problem already noted in the introduction. Standardized and anonymized repositories of injured player footprints could therefore play a critical role in advancing injury modeling and predictive analytics in elite sport.

#### 2.3.1 The calculation of FWF cumulative variation’s matrix: A generalization and a temporal diversification of acute workload (AW).

In sports science, Acute Workload (AW) is most commonly defined as the amount of external load accumulated over a one-week period, including both training and match data [61]. This metric is widely used to estimate player ‘fatigue’.

Following the conventional definition used in workload monitoring in team sports, as proposed by Gabbett (2016) [7] and continuing with the notation defined in Eq (6), the AW for a footballer ‘${\text{f}}_{\text{k}}$’ over *n* = 7 season events ‘${\text{e}}_{\text{j}}$’, for a given external workload variable ‘${\text{v}}_{\text{i}}$’, is calculated as summarized in Table 3.

**Table 3:** Example of calculus of AW (‘*f*atigue’).

$X_{{\text{v}}_{\text{i}}}(n=\{e_{1},e_{2},...,e_{7}\})=[\begin{array}{cccc} x_{{\text{v}}_{\text{i}}}(e_{1}) & x_{{\text{v}}_{\text{i}}}(e_{2}) & \cdots & x_{{\text{v}}_{\text{i}}}(e_{7}) \end{array}]$
where:
$x_{{\text{v}}_{\text{i}}}(e_{1})=1.5$ units,
$x_{{\text{v}}_{\text{i}}}(e_{2})=0.33$ units,
$x_{{\text{v}}_{\text{i}}}(e_{3})=1.5$ units,
$x_{{\text{v}}_{\text{i}}}(e_{4})=0.33$ units,
$x_{{\text{v}}_{\text{i}}}(e_{5})=1.5$ units,
$x_{{\text{v}}_{\text{i}}}(e_{6})=0.33$ units,
$x_{{\text{v}}_{\text{i}}}(e_{7})=1.5$ units.

$AW=x_{{\text{v}}_{\text{i}}}(e_{1})+x_{{\text{v}}_{\text{i}}}(e_{2})+\cdots +x_{{\text{v}}_{7}}(e_{7})=1.5+0.33+1.5+0.33+1.5+0.33+1.5=7.$


Note: *Example calculated for a footballer ‘${\text{f}}_{\text{k}}$’ who trains or plays a game in seven different event seasons ‘${\text{e}}_{\text{j}}$’ with a specific temporal workload distribution, for an external variable workload ‘${\text{v}}_{\text{i}}$*

However, the rationale behind limiting the calculation window to exactly seven events deserves critical reflection [62]. Equally important is the question of which day of the week is most relevant for computing AW. We propose that managing and storing a broader range of temporal views of this feature—denoted by the parameter τ—could enrich datasets and improve the performance of machine learning models designed to assess injury risk.

Accordingly, based on the principles of discrete-time integral calculus [40], we propose a generalization of the acute workload calculation. For each variable ‘${\text{v}}_{\text{i}}$’, and for each player ‘${\text{f}}_{\text{k}}$’, we compute the cumulative load over a rolling window of size τ≥1 ending at event ‘${\text{e}}_{\text{j}}$’. We name this generalized metric ‘*tau-acute-workload*’, denoted as ‘${AW}_{v_{\text{i}}}(n=[e_{j}],\tau )$’, and define it formally in Eq (9).

$$AW_{{\text{v}}_{\text{i}}}(n=[e_{\text{j}}],\tau {)}_{f_{k}}=\sum_{z=e_{j}}^{e_{j-(\tau -1)}}X_{v_{i}}(z)=x_{{\text{v}}_{\text{i}}}(e_{\text{j}})+x_{{\text{v}}_{\text{i}}}(e_{\text{j-1}})+...+x_{{\text{v}}_{\text{i}}}(e_{\text{j}-(\tau -2)})+x_{{\text{v}}_{\text{i}}}(e_{\text{j}-(\tau -1)})$$ (9)

**Fig 3:**
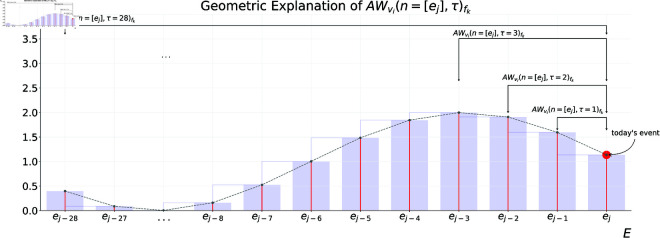
Geometric explanation of ‘${AW}_{v_{i}}(n=[e_{j}],\tau )$’. Figure shows how to calculate it for an external workload variable ‘${\text{v}}_{\text{i}}$’ extracted from a GPS unit of a footballer ‘${\text{f}}_{\text{k}}$’ in an event ‘${\text{e}}_{\text{j}}$’ during a specific τ∈T, T={1,2,3,...,τ=28} windows size of previous events.

Fig 3 provides a geometrical illustration that can assist the reader in understanding the calculation process.

Consequently, we also show how to calculate the ‘*FWF cumulative variations*’ matrix of a set *T* of several τ≥1 windows size Eq (10):

$$\sum^{T}FWF_{V\times \;E}\;(n{)}_{f_{k}}={\left(\begin{array}{cccc} AW_{v_{1}}(n=[e_{j}],1{)}_{f_{k}} & AW_{v_{1}}(n=[e_{j}],2{)}_{f_{k}} & \cdots & AW_{v_{1}}(n=[e_{j}],\tau {)}_{f_{k}}\\ AW_{v_{2}}(n=[e_{j}],1{)}_{f_{k}} & AW_{v_{2}}(n=[e_{j}],2{)}_{f_{k}} & \cdots & AW_{v_{2}}(n=[e_{j}],\tau {)}_{f_{k}}\\ \vdots & \vdots & \ddots & \vdots \\ AW_{v_{i}}(n=[e_{j}],1{)}_{f_{k}} & AW_{v_{i}}(n=[e_{j}],2{)}_{f_{k}} & \cdots & AW_{v_{i}}(n=[e_{j}],\tau {)}_{f_{k}} \end{array}\right)}_{\#(V)\;\times \;(\#E\cdot \#T)}$$ (10)

where:

(i) $f_{\text{k}}\in F$, $F=\{{\text{f}}_{1},{\text{f}}_{2},...,{\text{f}}_{\text{k}},...,{\text{f}}_{\text{totalfootballers}}\}$ (Eq (1)).
(ii) n∈E, $E=\{{\text{e}}_{1},{\text{e}}_{2},...,{\text{e}}_{\text{j}}\}$ is a subset of season events considered from the total set of events (Eq (2).
(iii) $v_{\text{i}}\in V$, $V=\{{\text{v}}_{1},{\text{v}}_{2},...,{\text{v}}_{\text{i}}\}$ is a subset of variables considered from the total set of external workload variables sampled and extracted (Eq (4) from ‘*k*’ sensors of ${\text{GPS}}_{\text{RAW}}(\text{n}{)}_{\text{f}}^{{\text{e}}_{\text{j}}}$ (Eq (3)).
(iv) τ∈T, T={1,2,...,τ}
(v) #(V) = total ‘${\text{v}}_{\text{i}}$’ variables considered.
(vi) #(E) = total ‘${\text{e}}_{\text{j}}$’ events considered.
(vii) #(T) = total different ‘τ’ windows sizes of past events considered.

The feature engineering method that we have developed, termed the ‘*Calculation of Cumulative Variations of an External Workload Footprint of a Footballer*’, assists in the generalization and calculation of acute workload (AW) for a specific event ‘${\text{e}}_{\text{j}}$’ across various window sizes denoted by ‘τ’. It enables the consolidation and management of all cumulative variations in acute workloads for a set of variables associated with an event ‘${\text{e}}_{\text{j}}$’ under different window sizes, all within a single framework.

#### 2.3.2 The calculation of FWF temporary variations’ matrix: The generalization and temporal diversification of chronic workload (CW) and a new perspective for ACWR calculation.

Following the methodology described by Gabbett (2016), Chronic Workload (CW) is typically defined as the 4-week (28-day) rolling average of acute workloads (AW) [8]. In terms of Eq (9), CW for an external workload variable ‘${\text{v}}_{\text{i}}$’ extracted from a GPS unit of a footballer ‘${\text{f}}_{\text{k}}$’ in an event ‘${\text{e}}_{\text{j}}$’ is computed as shown in Eq (11).

$$CW28_{{\text{v}}_{\text{i}}}(n=[e_{\text{j}}]{)}_{f_{k}}=\frac{AW_{v_{i}}(n=[e_{j}],7{)}_{f_{k}}+AW_{v_{i}}(n=[e_{j-7}],7{)}_{f_{k}}+AW_{v_{i}}(n=[e_{j-14}],7{)}_{f_{k}}+AW_{v_{i}}(n=[e_{j-21}],7{)}_{f_{k}}}{4}\;=\frac{\sum_{w_{1}=e_{j}}^{e_{j-6}}X_{v_{i}}(w_{1})+\sum_{w_{2}=e_{j-7}}^{e_{j-13}}X_{v_{i}}(w_{2})+\sum_{w_{3}=e_{j-14}}^{e_{j-20}}X_{v_{i}}(w_{3})+\sum_{w_{4}=e_{j-21}}^{e_{j-27}}X_{v_{i}}(w_{4})}{4}\;=\frac{x_{{\text{v}}_{\text{i}}}(e_{\text{j}})+x_{{\text{v}}_{\text{i}}}(e_{\text{j}-1})+\ldots +x_{{\text{v}}_{\text{i}}}(e_{\text{j}-26})+x_{{\text{v}}_{\text{i}}}(e_{\text{j}-27})}{4}$$ (11)

This value serves as an indicator of accumulated training over a broader time window, and is often interpreted as a proxy for an athlete’s ‘fitness’. High chronic workload is generally associated with improved resilience and reduced injury risk [63], as it reflects ongoing physiological adaptation to repeated load [64].

In accordance with Gabbett (2016) [8], the Acute:Chronic Workload Ratio (ACWR), ‘${\text{ACWR}}_{RA}$’ (*RA* = ‘*rolling averages*’) or ‘${\text{ACWR}}_{\text{COUPLED}}$’, as currently calculated and used by specialists, calculates for an external workload variable‘${\text{v}}_{\text{i}}$’ of a footballer ‘${\text{f}}_{\text{k}}$’ in an event ‘${\text{e}}_{\text{j}}$’, the ratio between the acute workload (AW), or ‘fatigue’ and the average of the last 4 weeks of chronic workload (CW) [61] or ‘fitness’, as described in equation Eq (12)

$$ACWR=ACWR_{\text{RA}}=ACWR_{{\text{COUPLED}}_{{\text{v}}_{\text{i}}}}(n=[e_{\text{j}}]{)}_{f_{k}}=\frac{AW_{v_{i}}(n=[e_{j}],7{)}_{f_{k}}}{CW28_{{\text{v}}_{\text{i}}}(n=[e_{\text{j}}]{)}_{f_{k}}}$$ (12)

By meticulously recording both, acute and chronic training loads, and subsequently modeling the ACWR, practitioners gain the ability to discern whether athletes are in a state of ‘fitness’ (indicative of net training recovery and a below-average risk of injury) or ‘fatigue’ (signifying net training stress and an above-average risk of injury) [8]. Furthermore, the utilization of ACWR equips practitioners with the means to obtain a comprehensive overview of an athlete’s training and match load history [5]. This facilitates a more streamlined assessment of readiness, aiding in the formulation of better training plans and periodization strategies [61]. Moreover, ACWR serves as a pivotal indicator for injury risk [9], acting as an early warning system, and consequently contributing to performance enhancement [3].

However, recent research has raised doubts about the suitability of the ACWR as a reliable approach for modeling training loads [52]. Furthermore, some researchers argue that despite the observed association, ACWR does not effectively predict non-contact injuries among elite footballers [11]. Impellizeri et al. [10], even suggest that “*ACWR be dismissed as a framework and model, and in line with this, injury frameworks, recommendations, and consensus should be updated to reflect the lack of predictive value of and statistical artifacts inherent in ACWR models*”. From a mathematical standpoint, the ratio is essentially a form of proportional calculation. It quantifies whether the training load in the most recent week is disproportionately different from the average of the past four weeks. In particular, the same acute chronic workload ratio can be achieved through various temporal distributions of acute workloads, potentially leading to spurious correlations [12].

To illustrate this, let’s consider an example involving three soccer players (‘${\text{f}}_{1}$’, ‘${\text{f}}_{2}$’, and ‘${\text{f}}_{3}$’), who had the same training loads in the three weeks leading up to the present week. However, disparities in their training and/or competition schedules emerged in the current week, as detailed below in Table 4. Evidently, all three soccer players exhibit identical ACWR values. Now, when considering only their training loads, the fitness coach must decide which of the three players to place greater trust in for competition, with the aim of minimizing injury risk. Would it be reasonable to assert that ‘${\text{f}}_{2}$’ faces a heightened risk of injury in the upcoming event compared to ‘${\text{f}}_{1}$’ and ‘${\text{f}}_{3}$’, due to the less progressive distribution of their training load? Similarly, if the choice is between ‘${\text{f}}_{1}$’ and ‘${\text{f}}_{3}$’, which player should be considered the safer option?.

**Table 4:** Illustration of the temporal distribution of training loads over 4 weeks (28 events) for 3 distinct soccer players, alongside corresponding acute to chronic workload ratio (ACWR) calculations.

‘${\text{f}}_{1}$’
$w1=[1.5,0.33,1.5,0.33,1.5,0.33,1.5]\Rightarrow AW(w1{)}_{f_{1}}=7units$
$w2=[1.5,0.33,1.5,0.33,1.5,0.33,1.5]\Rightarrow AW(w2{)}_{f_{1}}=7units$
$w3=[1.5,0.33,1.5,0.33,1.5,0.33,1.5]\Rightarrow AW(w3{)}_{f_{1}}=7units$
$w4=[0,0,0,0,0,0,7]\Rightarrow AW(w4{)}_{f_{1}}=7units$

$CW(w1,w2,w3,w4{)}_{f_{1}}=\text{avg}[AW(w1),AW(w2),AW(w3),AW(w4)]=7units$
$ACWR(w1,w2,w3,w4{)}_{f_{1}}=AW(w4{)}_{f_{1}}/CW(w1,w2,w3,w4{)}_{f_{1}}=7/7=1unit$

‘${\text{f}}_{2}$’
$w1=[1.5,0.33,1.5,0.33,1.5,0.33,1.5]\Rightarrow AW(w1{)}_{f_{2}}=7units$
$w2=[1.5,0.33,1.5,0.33,1.5,0.33,1.5]\Rightarrow AW(w2{)}_{f_{2}}=7units$
$w3=[1.5,0.33,1.5,0.33,1.5,0.33,1.5]\Rightarrow AW(w3{)}_{f_{2}}=7units$
$w4=[1,1,1,1,1,1,1]\Rightarrow AW(w4{)}_{f_{2}}=7units$

$CW(w1,w2,w3,w4{)}_{f_{2}}=\text{avg}[AW(w1),AW(w2),AW(w3),AW(w4)]=7units$
$ACWR(w1,w2,w3,w4{)}_{f_{2}}=AW(w4{)}_{f_{2}}/CW(w1,w2,w3,w4{)}_{f_{2}}=7/7=1unit$

‘${\text{f}}_{3}$’
$w1=[1.5,0.33,1.5,0.33,1.5,0.33,1.5]\Rightarrow AW(w1{)}_{f_{3}}=7units$
$w2=[1.5,0.33,1.5,0.33,1.5,0.33,1.5]\Rightarrow AW(w2{)}_{f_{3}}=7units$
$w3=[1.5,0.33,1.5,0.33,1.5,0.33,1.5]\Rightarrow AW(w3{)}_{f_{3}}=7units$
$w4=[1.5,0.33,1.5,0.33,1.5,0.33,1.5]\Rightarrow AW(w4{)}_{f_{3}}=7units$

$CW(w1,w2,w3,w4{)}_{f_{3}}=\text{avg}[AW(w1),AW(w2),AW(w3),AW(w4)]=7units$
$ACWR(w1,w2,w3,w4{)}_{f_{3}}=AW(w4{)}_{f_{3}}/CW(w1,w2,w3,w4{)}_{f_{3}}=7/7=1unit$


Note: *The calculation of the ACWR is the same despite the different temporal distribution of the training loads.*

This scenario underscores the fact that relying solely on ‘${\text{ACWR}}_{\text{COUPLED}}$’ disregards a substantial amount of information concerning the monotony of the load, including its temporal progression and regression, not resolved either by new more sophisticated calculation proposed by Windt and Gabbett (2019) [12], such as ‘${\text{ACWR}}_{\text{UNCOUPLED}}$’ where the chronic workload is computed over a rolling 3-week period (CW21) as defined in Eqs (13) and (14),

$$ACWR_{{\text{UNCOUPLED}}_{{\text{v}}_{\text{i}}}}(n=[e_{\text{j}}]{)}_{{\text{f}}_{\text{k}}}=\frac{AW_{{\text{v}}_{\text{i}}}(n=[e_{\text{j}}],7{)}_{f_{k}}}{CW21_{{\text{v}}_{\text{i}}}(n=[e_{\text{j}}]{)}_{f_{k}}}$$ (13)

$$CW21_{{\text{v}}_{\text{i}}}(n=[e_{\text{j}}]{)}_{{\text{f}}_{\text{k}}}=\frac{AW_{v_{i}}(n=[e_{j-7}],7{)}_{f_{k}}+AW_{v_{i}}(n=[e_{j-14}],7{)}_{f_{k}}+AW_{v_{i}}(n=[e_{j-21}],7{)}_{f_{k}}}{3}\;=\frac{\sum_{w_{2}=e_{j-7}}^{e_{j-13}}X_{v_{i}}(w_{2})+\sum_{w_{3}=e_{j-14}}^{e_{j-20}}X_{v_{i}}(w_{3})+\sum_{w_{4}=e_{j-21}}^{e_{j-27}}X_{v_{i}}(w_{4})}{3}\;=\frac{x_{{\text{v}}_{\text{i}}}(e_{\text{j}-7})+x_{{\text{v}}_{\text{i}}}(e_{\text{j}-8})+\ldots +x_{{\text{v}}_{\text{i}}}(e_{\text{j}-26})+x_{{\text{v}}_{\text{i}}}(e_{\text{j}-27})}{3}$$ (14)

or calculating acute:chronic workload ratios following the Exponentially Weighted Moving Average (EWMA) methodology proposed by Murray et al. (2017), ‘${\text{ACWR}}_{\text{EWMA}}$’ [16] defined in Eqs (15) and (16)

$$ACWR_{{\text{EWMA}}_{{\text{v}}_{\text{i}}}}(n=[e_{\text{j}}]{)}_{f_{k}}=\frac{AW_{{\text{EWMA}}_{{\text{v}}_{\text{i}}}}(n=[e_{j}],7{)}_{f_{k}}}{AW_{{\text{EWMA}}_{{\text{v}}_{\text{i}}}}(n=[e_{\text{j}}],28{)}_{f_{k}}}$$ (15)

$$AW_{{\text{EWMA}}_{{\text{v}}_{\text{i}}}}(n=[e_{j}],N{)}_{f_{k}}=AW_{v_{i}}(n=[e_{j}],\tau =N{)}_{f_{k}}\cdot \frac{2}{N+1}+(1-(\frac{2}{N+1})\cdot AW_{v_{i}}(n=[e_{j-1}],\tau =N{)}_{f_{k}})$$ (16)

or ‘${\text{ACWR}}_{\text{REDI}}$’ [23], experimental and not widespread among professionals.

As a result, it seems that decision models, whether they are designed by humans or generated by machines, which rely solely on these characteristics, may not meet the desired standards for effectively assessing the risk of non-contact muscle injuries that are primarily attributed to imbalanced training loads.

Consequently, given the multifaceted challenges outlined above, based on differential calculus of a discrete temporal series (DTS) [40], we propose to calculate for an external workload variable ‘${\text{v}}_{\text{i}}$’ extracted from a GPS unit of a footballer ‘${\text{f}}_{\text{k}}$’ the difference of ‘*tau-acute-workloads*’ between an event ‘${\text{e}}_{\text{j}}$’ and the previous ‘${\text{e}}_{j-1}$’, during a specific τ≥1 windows size of previous events, a value named as ‘*delta-acute-cronic-workload*’, and denoted as ‘$ACW_{{\text{v}}_{\text{i}}}(n=[e_{\text{j}}],\tau )$’, as described in equation Eq (17):

$$ACW_{v_{i}}(n=[e_{j}],\tau {)}_{f_{k}}=\Delta [AW_{v_{i}}(n=[e_{j}],\tau {)}_{f_{k}}]\;=AW_{v_{i}}(n=[e_{j}],\tau {)}_{f_{k}}-AW_{v_{i}}(n=[e_{j-1}],\tau {)}_{f_{k}}\;=\sum_{z_{1}=e_{j}}^{e_{j-(\tau -1)}}X_{v_{i}}(z_{1})-\sum_{z_{2}=e_{j-1}}^{e_{j-1-(\tau -1)}}X_{v_{i}}(z_{2})\;=\sum_{z_{1}=e_{j}}^{e_{j-(\tau -1)}}X_{v_{i}}(z_{1})-\sum_{z_{2}=e_{j-1}}^{e_{j-\tau }}X_{v_{i}}(z_{2})\;=\left(x_{v_{i}}(e_{j})+x_{v_{i}}(e_{j-1})+\ldots +x_{v_{i}}(e_{j-(\tau -2)})+x_{v_{i}}(e_{j-(\tau -1)})\right)\;\;-\left(x_{v_{i}}(e_{j-1})+x_{v_{i}}(e_{j-2})+\ldots +x_{v_{i}}(e_{j-(\tau -1)})+x_{v_{i}}(e_{j-\tau })\right)\;=x_{v_{i}}(e_{j})-x_{v_{i}}(e_{j-\tau })$$ (17)

**Fig 4:**
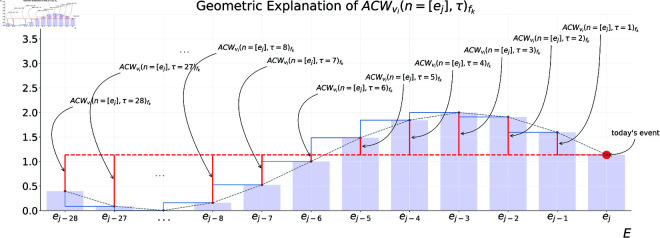
Geometric explanation of ‘$ACW_{{\text{v}}_{\text{i}}}(n=[e_{\text{j}}],\tau )$’. Figure shows how to calculate it for an external workload variable ‘${\text{v}}_{\text{i}}$’ extracted from a GPS unit of a footballer ‘${\text{f}}_{\text{k}}$’ in an event ‘${\text{e}}_{\text{j}}$’ during a specific τ∈T, T={1,2,3,4,5,6,7,8,...,τ=28} windows size of previous events.

Fig 4 provides a geometrical illustration that can aid the reader in comprehending the calculation process.

Furthermore, we provide a detailed explanation of how to compute the ‘*FWF temporary variations*’ matrix, which can be conceptualized as a discrete-time causal system [40]:

$$\Delta^{T}FWF_{V\times \;E}\;(n{)}_{f_{k}}={\left(\begin{array}{cccc} ACW_{{\text{v}}_{1}}(n=[e_{\text{j}}],1{)}_{f_{k}} & ACW_{{\text{v}}_{1}}(n=[e_{\text{j}}],2{)}_{f_{k}} & \cdots & ACW_{{\text{v}}_{1}}(n=[e_{\text{j}}],\tau {)}_{f_{k}}\\ ACW_{{\text{v}}_{2}}(n=[e_{\text{j}}],1{)}_{f_{k}} & ACW_{{\text{v}}_{2}}(n=[e_{\text{j}}],2{)}_{f_{k}} & \cdots & ACW_{{\text{v}}_{2}}(n=[e_{\text{j}}],\tau {)}_{f_{k}}\\ \vdots & \vdots & \ddots & \vdots \\ ACW_{{\text{v}}_{\text{i}}}(n=[e_{\text{j}}],1{)}_{f_{k}} & ACW_{{\text{v}}_{\text{i}}}(n=[e_{\text{j}}],2{)}_{f_{k}} & \cdots & ACW_{{\text{v}}_{\text{i}}}(n=[e_{\text{j}}],\tau {)}_{f_{k}} \end{array}\right)}_{\#(V)\;\times \;(\#E\cdot \#T)}$$ (18)

where:

(i) $f_{\text{k}}\in F$, $F=\{{\text{f}}_{1},{\text{f}}_{2},...,{\text{f}}_{\text{k}},...,{\text{f}}_{\text{totalfootballers}}\}$ (Eq (1)).
(ii) n∈E, $E=\{{\text{e}}_{1},{\text{e}}_{2},...,{\text{e}}_{\text{j}}\}$ is a subset of season events considered from the total set of events (Eq (2).
(iii) $v_{\text{i}}\in V$, $V=\{{\text{v}}_{1},{\text{v}}_{2},...,{\text{v}}_{\text{i}}\}$ is a subset of variables considered from the total set of external workload variables sampled and extracted (Eq (4) from ‘*k*’ sensors of ${\text{GPS}}_{\text{RAW}}(\text{n}{)}_{\text{f}}^{{\text{e}}_{\text{j}}}$ (Eq (3)).
(iv) τ∈T, T={1,2,...,τ}
(v) #(V) = total ‘${\text{v}}_{\text{i}}$’ variables considered.
(vi) #(E) = total ‘${\text{e}}_{\text{j}}$’ events considered.
(vii) #(T) = total different ‘τ’ *windows sizes of past events considered.*

The feature engineering method that we have developed is referred as the ‘*Calculation of Temporary Variations of an External Workload Footprint of a Footballer*’. As demonstrated previously, it is represented as another discrete temporal matrix. The primary objective of the ‘*FWF temporary variations’*’ matrix is to expand the perspective on ACWR ratios by addressing the gaps in their mathematical formulation, thereby striving to enhance and advance beyond the proposals outlined in the existing state of the art.

Firstly, the method generalizes the computation of acute loads by considering various window sizes for a range of previous events. Consequently, it enables the calculation of chronic loads for various time periods, which extend beyond the conventional 28-day period. Secondly, it calculates the differences in monotony for a given event concerning all past events deemed relevant for consideration.

This approach aims to provide a more comprehensive and nuanced understanding of workload dynamics in sports, not just soccer, contributing to improved injury risk assessment and performance optimization strategies.

#### 2.3.3 A sample graphical depiction of the cumulative and temporary variations of the FWF matrix: Aiding in the Explainability of Machine Learning Models.

Explainability in the context of machine learning refers to the ability of a model to articulate the rationale behind its predictions in terms that humans can understand. This aspect of artificial intelligence is particularly relevant, as it encompasses the broader effort to comprehend how and why a model arrives at specific decisions, especially important when dealing with complex or “*black box*” models that do not inherently provide insight into their internal working. [65]

Explainability is critical not only for trust and transparency, but also for model debugging and improvement. It helps to ensure that the model’s decisions are fair, adhere to ethical guidelines, and are free of bias. In addition, in regulated industries such as finance [66] and healthcare [67], explainability is essential to ensure safety and comply with legal standards that require explanations of algorithmic decisions.

In conclusion, the pursuit of explainability in machine learning is focused on making models as clear and understandable as possible, ensuring that their decisions can be trusted and effectively utilized in real-world applications. [68]

**Fig 5:**
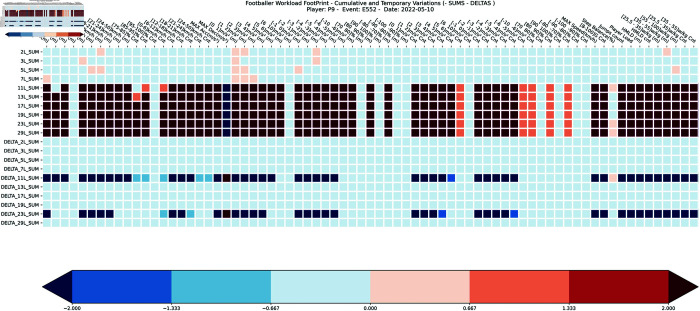
Example 1: Heatmap representation of the cumulative and temporary variations of FWF matrix for player 9 in event 552. Figure shows $\sum^{T}FWF_{V\times \;E}\;(n=[e_{j=552}]{)}_{f_{k=9}}$ and $\Delta^{T}FWF_{V\times E}(n=[e_{j=552}]{)}_{f_{k=9}}$ of a footballer ‘${\text{f}}_{\text{k}=9}$’ in an event ‘${\text{e}}_{\text{j}=554}$’ during a specific τ∈T, T={2,3,5,6,7,11,13,17,19,23,29} windows size of previous events.

**Fig 6:**
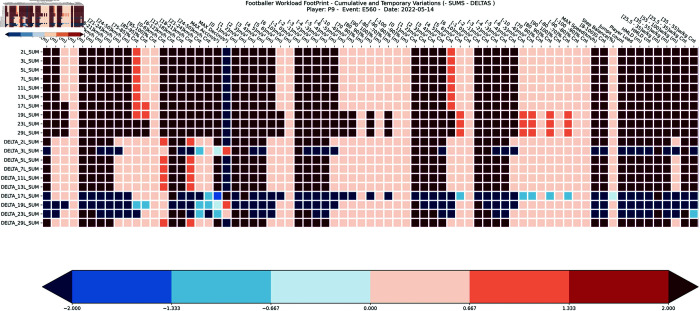
Example 2: Heatmap representation of the cumulative and temporary variations of FWF matrix for player 9 in event 560. Figure shows $\sum^{T}FWF_{V\times \;E}\;(n=[e_{j=552}]{)}_{f_{k=9}}$ and $\Delta^{T}FWF_{V\times E}(n=[e_{j=552}]{)}_{f_{k=9}}$ of a footballer ‘${\text{f}}_{\text{k}=9}$’ in an event ‘${\text{e}}_{\text{j}=560}$’ during a specific τ∈T, T={2,3,5,6,7,11,13,17,19,23,29} windows size of previous events.

For an effective visual representation of the *cumulative and temporary variations of the Footballer Workload Footprint (FWF)* matrix, used as a support tool in explaining machine learning models, specifically within soccer teams, we explored the possibility of creating heatmaps. A ‘*heatmap*’ is a popular method for visualizing matrix-like data by taking colors as aesthetic elements. It can condense complex information into an easy-to-understand format, helping stakeholders quickly grasp key patterns and anomalies without detailed statistical analysis [69]. An example of heatamps designed specifically to represent FWF is depicted in Figs 5 and 6.

In our domain problem, the x-axis of the heatmap could represent different subset of variables $V=\{{\text{v}}_{1},{\text{v}}_{2},...,{\text{v}}_{\text{i}}\}$ included in the FWF matrix. The y-axis might show different periods of cumulative and / or time variations, reflecting how the variable values changed during a specific τ∈T, T={1,2,3,4,5,6,7,8,...,τ=28} windows size of previous events, for a footballer ‘${\text{f}}_{\text{k}}$’ in an specific event ‘${\text{e}}_{\text{j}}$’. We use a color gradient to denote the magnitude of each variable’s value. Warmer colors (e.g., reds) could indicate higher values, and cooler colors (e.g., blues) lower values. This color coding enhances the ability to swiftly recognize and contrast the most active or influential variables within the heatmap. Additionally, it facilitates straightforward comparisons between different heatmaps for various events, whether pertaining to the same player or different players. Optionally, annotations can be added to highlight specific information such as events represented, the concrete footballer, date times, or trends in a specific variable, such as peak loads before an injury or deviations from a player’s normal range that could suggest potential risks or the need for recovery.

This method not only aids in understanding intricate machine learning models, but also allows for the comparison and discussion of various heatmaps (as pictured in Figs 5 and 6), thereby improving the communication of insights and suggestions to sports scientists, medical personnel, and non-technical stakeholders within sports organizations.

### 2.4 Exploratory and confirmatory analysis: Comparing the predictive effectiveness of ACWR against ‘*Cumulative and Temporal Variations of FWF*’

Does the calculation of ‘*Cumulative and Temporal variations of the FWF*’ matrix improve the predictive ability of the non-contact injury risk associated with the acute/chronic workload ratio (ACWR)? To evaluate the effectiveness of our proposed methodology, we utilized multivariate supervised techniques to perform confirmatory data analysis.

This analysis entailed comparing models trained using our innovative approach with those trained using the most commonly cited and used by practitioners, ACWR calculations: ‘${\text{ACWR}}_{COUPLED}$’ [7], ‘${\text{ACWR}}_{\text{UNCOUPLED}}$’ [8,12] , or ‘${\text{ACWR}}_{EWMA}$’ [15,16]. As illustrated in Fig 1, we initially established five distinct datasets to systematically assess, validate, and objectively compare the models. Furthermore, to obtain a holistic understanding of the data sets, we concurrently perform an exploratory analysis [42] in conjunction with the confirmatory analysis [43]. This approach guarantees an uninterrupted and all-encompassing grasp of the complexities of datasets. To rigorously assess model generalization and mitigate the risk of overfitting, we employed a 10-repeated stratified 2-fold cross-validation strategy [37,38]. This method ensures that models are trained and validated across multiple randomized splits while preserving the original class distribution. In addition, Principal Component Analysis (PCA) [70,71] was applied prior to model training, retaining 95% of the cumulative explained variance. Together, these techniques were implemented to balance bias and variance, and to provide robust, reliable model evaluation.

To perform all data preprocessing, feature engineering, model training, validation, and evaluation procedures, as describes Fig 1, we developed a computational pipeline based on Python 3.9. Our workflow was orchestrated using the scientific computing environment IPython [72], which provided an interactive framework for executing and managing experiments. For data management and transformation tasks, we employed Pandas [73], a high-level library offering versatile data structures and analytical operations, alongside Numpy [74], the core Python package for efficient numerical array computing. For statistical analysis, scientific computation, and matrix manipulation, we utilized SciPy [75]. To generate graphical representations of the results, we applied Matplotlib [76], enabling the creation of detailed and customizable visualizations. Regarding machine learning implementations, we used Scikit-learn [77], an extensive library encompassing state-of-the-art algorithms for classification, cross-validation strategies, dimensionality reduction, feature selection, oversampling, and model evaluation metrics. All software components were used in their stable release versions as of September 2023, ensuring reproducibility and compatibility. The entire pipeline was designed to guarantee robust, transparent, and scalable experimental procedures.

As depicted in Fig 1, we designed five distinct datasets, based in the same data collected, extracted and cleaned. We modeled all datasets using ‘FWF_*V*× *E*_ (*n*)_*f*_*k*__’ Eq (7) and ‘$L_{f_{k}}(n)$’ Eq (8) matrixes and applied the respective feature engineering process to ‘*FWF*’. Summarizing, the final designs of the five datasets were as follows:

- **Dataset 1** = $\left[\begin{array}{c} \left(FWF_{V\times \;E}\;(n{)}_{f_{k}}|L_{f_{k}}(n)\right) \end{array}\right]$, $\forall v_{i}\in V$, $\forall f_{k}\in F$ but considering only $e_{j}\in E$ that each ‘${\text{f}}_{\text{k}}$’ participate in. Notably, if a player did not partake in an ‘${\text{e}}_{\text{j}}$’ session, then ‘$x_{{\text{v}}_{\text{i}}}(e_{\text{j}})$’ is omitted from consideration $\forall v_{i}\in V$. As a consequence, the ‘*footprint*’ size varies for each player ‘${\text{f}}_{\text{k}}$’ within the dataset. Moreover, we intentionally refrain from applying any feature engineering to this dataset. It is deliberately positioned as a baseline for comparative analysis, as the models exclusively will leverage the values of the event variables from the current day, in order to predict.
- **Dataset 2** = $\left[\begin{array}{c} \left(FWF_{V\times \;E}\;(n{)}_{f_{k}}|L_{f_{k}}(n)\right) \end{array}\right]$, $\forall v_{i}\in V$, $\forall e_{j}\in E$, $\forall f_{k}\in F$, where:
  $$FWF_{V\times \;E}\;(n{)}_{f_{k}}=(\begin{array}{c} X_{v_{1}}(n)\\ X_{v_{2}}(n)\\ \vdots \\ X_{v_{i}}(n) \end{array})={\left(\begin{array}{cccc} AW_{v_{1}}(n=[e_{j}],7{)}_{f_{k}} & CW21_{{\text{v}}_{1}}(n=[e_{\text{j}}]{)}_{f_{k}} & ACWR_{{UNCOUPLED}_{{\text{v}}_{1}}}(n=[e_{\text{j}}]{)}_{f_{k}} & \\ AW_{v_{2}}(n=[e_{j}],7{)}_{f_{k}} & CW21_{{\text{v}}_{2}}(n=[e_{\text{j}}]{)}_{f_{k}} & ACWR_{{UNCOUPLED}_{{\text{v}}_{2}}}(n=[e_{\text{j}}]{)}_{f_{k}} & \\ \vdots & \vdots & \vdots \\ AW_{v_{i}}(n=[e_{j}],7{)}_{f_{k}} & CW21_{{\text{v}}_{\text{i}}}(n=[e_{\text{j}}]{)}_{f_{k}} & ACWR_{{UNCOUPLED}_{{\text{v}}_{\text{i}}}}(n=[e_{\text{j}}]{)}_{f_{k}} \end{array}\right)}_{\#(V)\;\times \;\#(E)}$$
  Notably, if a player did not partake in an ‘${\text{e}}_{\text{j}}$’ session, then ‘ $x_{{\text{v}}_{\text{i}}}(e_{\text{j}})$’ is assigned as‘*0*’ $\forall v_{i}\in V$. As a consequence, the ‘*footprint*’ size has the same dimension for each player $\forall f_{k}\in F$. We applied featuring engineering process based on ‘*AW7*’, ‘*CW21*’ and ‘${\text{ACWR}}_{\text{UNCOUPLED}}$’, so the models exclusively will leverage the values of the event variables from the current day based in ‘*AW7*’, ‘*CW21*’ and ‘${\text{ACWR}}_{\text{UNCOUPLED}}$’, in order to predict.
- **Dataset 3** = $\left[\begin{array}{c} \left(FWF_{V\times \;E}\;(n{)}_{f_{k}}|L_{f_{k}}(n)\right) \end{array}\right]$, $\forall v_{i}\in V$, $\forall e_{j}\in E$, $\forall f_{k}\in F$, where:
  $$FWF_{V\times \;E}\;(n{)}_{f_{k}}=(\begin{array}{c} X_{v_{1}}(n)\\ X_{v_{2}}(n)\\ \vdots \\ X_{v_{i}}(n) \end{array})={\left(\begin{array}{cccc} AW_{v_{1}}(n=[e_{j}],7{)}_{f_{k}} & CW28_{{\text{v}}_{1}}(n=[e_{\text{j}}]{)}_{f_{k}} & ACWR_{{COUPLED}_{{\text{v}}_{1}}}(n=[e_{\text{j}}]{)}_{f_{k}} & \\ AW_{v_{2}}(n=[e_{j}],7{)}_{f_{k}} & CW28_{{\text{v}}_{2}}(n=[e_{\text{j}}]{)}_{f_{k}} & ACWR_{{COUPLED}_{{\text{v}}_{2}}}(n=[e_{\text{j}}]{)}_{f_{k}} & \\ \vdots & \vdots & \vdots \\ AW_{v_{i}}(n=[e_{j}],7{)}_{f_{k}} & CW28_{{\text{v}}_{\text{i}}}(n=[e_{\text{j}}]{)}_{f_{k}} & ACWR_{{COUPLED}_{{\text{v}}_{\text{i}}}}(n=[e_{\text{j}}]{)}_{f_{k}} \end{array}\right)}_{\#(V)\;\times \;\#(E)}$$
  Notably, if a player did not partake in an ‘${\text{e}}_{\text{j}}$’ session, then ‘ $x_{{\text{v}}_{\text{i}}}(e_{\text{j}})$’ is assigned as ‘0’ $\forall v_{i}\in V$. As a consequence, the ‘*footprint*’ size has the same dimension for each player $\forall f_{k}\in F$. We applied featuring engineering process based on ‘*AW7*’, ‘*CW28*’ and ‘${\text{ACWR}}_{\text{COUPLED}}$’, so the models exclusively will leverage the values of the event variables from the current day based in ‘*AW7*’, ‘*CW28*’ and ‘${\text{ACWR}}_{\text{COUPLED}}$’, in order to predict.
- **Dataset 4** = $\left[\begin{array}{c} \left(FWF_{V\times \;E}\;(n{)}_{f_{k}}|L_{f_{k}}(n)\right) \end{array}\right]$, $\forall v_{i}\in V$, $\forall e_{j}\in E$, $\forall f_{k}\in F$, where:
  $$FWF_{V\times \;E}\;(n{)}_{f_{k}}=(\begin{array}{c} X_{v_{1}}(n)\\ X_{v_{2}}(n)\\ \vdots \\ X_{v_{i}}(n) \end{array})={\left(\begin{array}{cccc} AW_{{\text{EWMA}}_{v_{1}}}(n=[e_{j}],7{)}_{f_{k}} & AW{\text{EWMA}}_{v_{1}}(n=[e_{j}],28{)}_{f_{k}} & ACWR_{{EWMA}_{v_{1}}}(n=[e_{\text{j}}]{)}_{f_{k}} & \\ AW_{{\text{EWMA}}_{v_{2}}}(n=[e_{j}],7{)}_{f_{k}} & AW_{{\text{EWMA}}_{v_{2}}}(n=[e_{j}],28{)}_{f_{k}} & ACWR_{{EWMA}_{{\text{v}}_{2}}}(n=[e_{\text{j}}]{)}_{f_{k}} & \\ \vdots & \vdots & \vdots \\ AW_{{\text{EWMA}}_{v_{i}}}(n=[e_{j}],7{)}_{f_{k}} & AW_{{\text{EWMA}}_{v_{i}}}(n=[e_{j}],28{)}_{f_{k}} & ACWR_{{EWMA}_{{\text{v}}_{\text{i}}}}(n=[e_{\text{j}}]{)}_{f_{k}} \end{array}\right)}_{\#(V)\;\times \;\#(E)}$$
  Notably, if a player did not partake in an ‘${\text{e}}_{\text{j}}$’ session, then ‘ $x_{{\text{v}}_{\text{i}}}(e_{\text{j}})$’ is assigned as‘0’ $\forall v_{i}\in V$. As a consequence, the ‘*footprint*’ size has the same dimension for each player $\forall f_{k}\in F$. We applied featuring engineering process based on ‘${\text{AW7}}_{\text{EWMA}}$’, ‘${\text{CW28}}_{\text{EWMA}}$’ and ‘${\text{ACWR}}_{\text{EWMA}}$’, so the models exclusively will leverage the values of the event variables from the current day based in ‘${\text{AW7}}_{\text{EWMA}}$’, ‘${\text{CW28}}_{\text{EWMA}}$’ and ‘${\text{ACWR}}_{\text{EWMA}}$’, in order to predict.
- **Dataset 5** = $\left[\begin{array}{c} \left(\sum^{T}FWF_{V\times \;E}\;(n{)}_{f_{k}}^{T}|\Delta^{T}FWF_{V\times \;E}\;(n{)}_{f_{k}}^{T}|L_{f_{k}}(n)\right) \end{array}\right]$, $\forall v_{i}\in V$, $\forall e_{j}\in E$, $\forall f_{k}\in F$ and ∀τ∈T, T={1,2,...,τ=28}.
  Notably, if a player did not partake in an ‘${\text{e}}_{\text{j}}$’ session, then ‘ $x_{{\text{v}}_{\text{i}}}(e_{\text{j}})$’ is assigned as ‘0’ $\forall v_{i}\in V$. As a consequence, the ‘*footprint*’ size has the same dimension for each player $\forall f_{k}\in F$. We applied featuring engineering process based on $\sum^{T}FWF$ and $\Delta^{T}FWF$, so the models exclusively will leverage the values of the event variables from the current day based in $\sum^{T}FWF$ and $\Delta^{T}FWF$ with ∀τ∈T, T={1,2,…,τ=28}, in order to predict.

Subsequently, as delineated in Fig 1, we executed identical procedures for each individual dataset. Table 5 provides a concise summary of the progression of the five datasets over the course of the experiments, spanning various phases of the study. Additionally, it includes the key parameters associated with the training/validation method and model testing.

**Table 5:** Summary of the progression of the five datasets over the course of the experiment and key parameters associated with the training/validation method and model testing.

Dataset ID	Dataset 1	Dataset 2	Dataset 3	Dataset 4	Dataset 5
Number of Players	23	23	23	23	23
Variables	73	73	73	73	73
FWF Modelling	True	True	True	True	True
Events per player	Specific per player	Same for all players: 567	Same for all players: 567	Same for all players: 567	Same for all players: 567
Total Registers	4124	13041	13041	13041	13041
Feature Engineering	False	True - ACWR_*UNCOUPLED*_*v*_*i*___	True - ACWR_*COUPLED*_*v*_*i*___	True - ACWR_*EWMA*_*v*_*i*___	True - $\sum^{T}FWF_{V\times \;E}\;(n{)}_{f_{k}}$ and $\Delta^{T}FWF_{V\times \;E}\;(n{)}_{f_{k}}$
Dataset Dimension	X: (4124, 73) - L: 23	X: (13041, 216) - L: 23	X: (13041, 216) - L: 23	X: (13041, 216) - L: 23	X: (13041, 1460) - L: 23
Undersampling	True - X: (786, 73) - L: 21	True - X: (809, 216) - L: 21	True - X: (809, 216) - L: 21	True - X: (809, 216) - L: 21	True - X: (809, 1460) - L: 21
Outlier Detection	0,175 reduction factor	0,175 reduction factor	0.200 reduction factor	0.240 reduction factor	0.185 reduction factor
	X: (648,73) - L: 16	X(667,216) - L: 16	X: (647,216) - L: 16	X: (615,216) - L: 16	X: (655,1460) - L: 16
Data Splitting	preserves the sequence of events over time	preserves the sequence of events over time	preserves the sequence of events over time	preserves the sequence of events over time	preserves the sequence of events over time
	(80%/20%)	(80%/20%)	(80%/20%)	(80%/20%)	(80%/20%)
	${\text{X}}_{\text{train}}$: (494, 73) - L: 11	${\text{X}}_{\text{train}}$: (524, 216) - L: 11	${\text{X}}_{\text{train}}$: (518, 216) - L: 11	${\text{X}}_{\text{train}}$: (492, 216) - L: 11	${\text{X}}_{\text{train}}$: (524, 1460) - L: 11
	${\text{X}}_{\text{test}}$: (154, 73) - L: 5	${\text{X}}_{\text{test}}$: (143, 216) - L: 5	${\text{X}}_{\text{test}}$: (129, 216) - L: 5	${\text{X}}_{\text{test}}$: (123, 216) - L: 5	${\text{X}}_{\text{test}}$: (131, 1460) - L: 5
Oversampling	Smote over ${\text{X}}_{\text{train}}$	Smote over ${\text{X}}_{\text{train}}$	Smote over ${\text{X}}_{\text{train}}$	Smote over ${\text{X}}_{\text{train}}$	Smote over ${\text{X}}_{\text{train}}$
	${\text{X}}_{\text{train}}$: (966,73) - L: 483	${\text{X}}_{\text{train}}$: (1014,216) - L: 507	${\text{X}}_{\text{train}}$: (1026,216) - L: 513	${\text{X}}_{\text{train}}$: (962,216) - L: 481	${\text{X}}_{\text{train}}$: (708,1460) - L: 354
Feature Selection	Dimensionality Reduction PCA	Dimensionality Reduction PCA	Dimensionality Reduction PCA	Dimensionality Reduction PCA	Dimensionality Reduction PCA
	PCA 95% explained variance	PCA 95% explained variance	PCA 95% explained variance	PCA 95% explained variance	PCA 95% explained variance
	(3 components)	(6 components)	(6 components)	(7 components)	(20 components)
	${\text{X}}_{\text{train}}$: (966,3) - L: 483	${\text{X}}_{\text{train}}$: (1014,3) - L: 507	${\text{X}}_{\text{train}}$: (1026,6) - L: 513	${\text{X}}_{\text{train}}$: (962,7) - L: 481	${\text{X}}_{\text{train}}$: (708,20) - L: 354
	${\text{X}}_{\text{test}}$: (154, 3) - L: 5	${\text{X}}_{\text{test}}$: (143, 6) - L: 5	${\text{X}}_{\text{test}}$: (129, 6) - L: 5	${\text{X}}_{\text{test}}$: (123, 7) - L: 5	${\text{X}}_{\text{test}}$: (152, 20) - L: 5
Crossvalidation	10-Repeated-Stratified-2-Fold	10-Repeated-Stratified-2-Fold	10-Repeated-Stratified-2-Fold	10-Repeated-Stratified-2-Fold	10-Repeated-Stratified-2-Fold

Note: *For a detailed understanding, consider consulting the corresponding information included in Sect 2.4 Exploratory and Confirmatory Analysis.*

**Fig 7:**
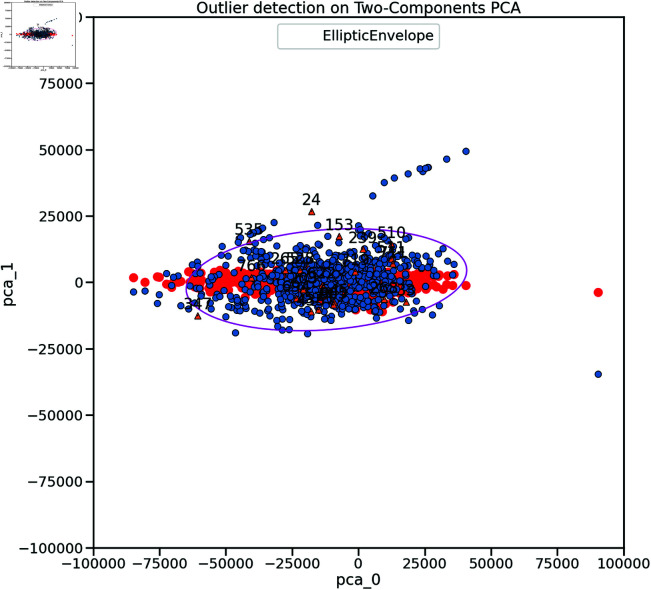
Example of robust PCA outlier detection. Visualization example for Dataset 5.

**Fig 8:**
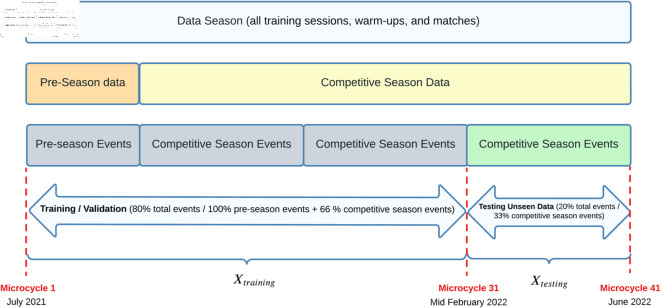
Design of training and testing dataset. Overview of the data splitting process during the confirmatory analysis.

The details for each of these phases are outlined below, providing a comprehensive justification and explanation for each phase:

1. **Undersampling Data Day Before Match**: Predicting injuries is considered a big challenge in AI/ML, mainly because of the highly imbalanced distribution of the two classes (injured vs non-injured) in real datasets. The imbalanced learning issue [29], named so by data science researchers, is concerned with the performance of learning algorithms in the presence of underrepresented data and severe class distribution skews [30]. Undersampling is a popular method in dealing with class-imbalance problems, which uses only a subset of the majority class and thus is very efficient, especially if applied with domain knowledge acquired through data exploration [78].
  Therefore, in order to balance the minority class, a rational exploratory undersampling based on domain knowledge was performed for the five designed datasets. We eliminated all samples that were not associated with the day before the match itself, what is the pre-match moment in which sport scientists ask themselves the key question: “*Can we trust that based on the current fitness levels of the footballer ‘${\text{f}}_{\text{k}}$’, indicated by ACWR calculation of the set ‘V’ of external workload variables, will perform adequately and not be injured if exposed to a competitive load?*” Similarly, the intelligent model must be designed and trained to ask the same question, but in return the possibility of predicting with this model whether a player will be injured in the next training session is lost. It should be noted that more than 90% of all muscle injuries and 51%–64% of joint/ligament injuries in soccer occur in non-contact situations, as reported by Lemes et al. [1]. Furthermore, these injury rates tend to be higher in matches compared to training sessions, according to Pfirrmann et al. [2]. Of the 23 non-contact injuries of all types (Table 2) reported throughout the entire season, only two occurred during a training session, which was not a significant loss for our study.
  Therefore, we decided to exclude from our datasets any pattern that contained information about the player’s training sessions prior to the match in which injury risk was assessed, except for the last one. In other words, the MD-1 events of the microcycles [79] were kept. This means that patterns related to the player’s workload during the match (MD) was also excluded. Furthermore, samples related to players who were not included in the match or did not participate in any minutes of the match were also excluded from the datasets. Consequently, we retained only the patterns from the previous training (MD-1), of those players who participated in the match (MD) and we associated the injury label corresponding to the event (MD-1), in case it occurred on the day of the match (MD). The injury label (Eq (5)) can be directly related to these specific MD-1 temporal events through the modeling capabilities of the ‘*footprint*’ (Eq (7)). This linkage facilitates the experimental execution to determine whether, with the information contained in each pre-match sample from each dataset, the trained models have the requisite capacity to predict a non-contact muscle injury. Additionally, it implies a fair design of experiment to compare which dataset among the five will provide the highest predictive capacity the day before a match.
  In conclusion, undersampling the five datasets the day before the match, allows us to achieve two key objectives: first, reduce the prevalence of the majority class, and thus achieve a more balanced class distribution (although imbalance still remains); and second, structure the datasets in such a way that enables an objective and fair comparison between the several calculations of the acute: chronic workload ratio (ACWR), and our newly proposed model: the Footballer Workload Footprint (FWF) along with the calculation of cumulative and temporary variations.
  Further details of the evolution of the five datasets after applying this phase of the experiment can be found in Table 5. .
2. **Outlier Detection**: In data analysis, outliers can pose challenges as they have the potential to significantly impact the results. Robust statistics is a methodology employed to identify outliers by looking for the model that fits the best with the majority of data [80]. Following data undersampling, a commonly used technique for outlier detection known as ‘Robust PCA Outlier Detection’ [80] was applied to all five datasets. A reduction factor of approximately 20% was applied, decreasing the set of samples considered and affecting the number of injuries considered, as detailed in Table 5.
  It was observed that, after applying the method, some samples labeled as injured were excluded during the process (i.e Fig 7)), and a significant portion of these outlier injuries occurred after a previous injury had already occurred. These types of injuries are known to reduce the predictive capability of the models, as demonstrated in the Carey modeling study [52]. From the perspective of sports science experts, it is suggested that inadequate recovery and re-adaptation after an injury can lead to new injuries that are not solely attributed to fitness or fatigue [52]. Consequently, these injuries were not aligned with the target injuries that were the focus of our study.
  Further details of the evolution of the five datasets after applying this phase of the experiment can be found in Table 5.
3. **Data Splitting**: In the field of machine learning, a fundamental objective is to construct computational models that exhibit strong predictive and generalization capabilities [81]. In the context of supervised learning, these models are trained to predict the outputs of an unknown target function. The target function is defined by a finite training dataset, denoted as “${\text{X}}_{\text{training}}$”, consisting of input examples and their corresponding desired outputs or labels (“${\text{y}}_{\text{training}}$”). At the end of the training process, the final model should not only predict the correct outputs for the input samples in “$X_{\text{training}}$” but also demonstrate effective generalization to previously ‘*unseen data*’, referred to as “$X_{\text{testing}}$”.
  As a result, it is imperative to distinguish between training data and testing data for the five datasets included in our experiment. In our approach, we segregated the samples in accordance with a logical temporal sequence that aligns with the study objectives and the expertise of sports science professionals (Fig 8).
  For all five datasets, we employed the samples from the pre-season through Mid-February, which constitute 80% of the total, as the training set. The remaining 20% was designated as the testing set, encompassing data that was never exposed to the trained models at any point during the training phase. Furthermore, the test set preserves the initial data distribution, posing a realistic challenge for the models that were trained.
  This testing period spans from mid-February until the end of the season, which accounts for 33% of the events in the competitive period, is critical phase in professional soccer when titles are decided, matches become considerably more physically demanding and consequently increase the effects of physical fatigue on the performance of players [82]. During this period, it becomes even more crucial and useful, if possible, to predict non-contact muscular injuries since losing a soccer player at this stage due to such an injury can result in an extended period of absence from 8 days to 3 months [83], encompassing physiological recovery and the process of re-adaptation for a safe return to competition.
  Further details of the evolution of the five datasets after applying this phase of the experiment can be found in Table 5.
4. **Data Oversampling in Training Datasets**: After data splitting process (Fig 8), the five training datasets obtained are highly imbalanced [84] (one class is significantly underrepresented in the data compared to the others), e.g. there is a very small percentage of samples of injured footballers. The main reason is that physical trainers work precisely to avoid these injuries, by daily adjusting and supervising the work loads. Thus, in order to avoid overfitting in the classification models (which will imply problems to predict the injury class) we have generated synthetic samples of the minority class using Synthetic Minority Over-sampling Technique (SMOTE).
  SMOTE [85] is a data augmentation or oversampling method used in machine learning and data analysis to address the problem of class imbalance. The SMOTE algorithm works by selecting a sample from the minority class and then identifying its *k* nearest neighbors. Synthetic samples are then created by interpolating between the original sample and these neighbors. This creates new samples that are similar to the original minority class data, but with slightly different features. This increasing in the amount of samples of injuries will help to balance the dataset and improve the performance of the applied machine learning models. In our study, we kept the same *k* value for all five datasets, seeking to prevent fairness in the results. SMOTE is a widely used technique in hundreds of works in the literature [86].
  Further details of the evolution of the five datasets after applying this phase of the experiment can be found in Table 5.
5. **Feature Selection. Dimensionality Reduction PCA in Training and Testing Datasets**: Many of the external training load variables ‘${\text{v}}_{i}$’ collected and extracted from GPS (Eq (3), were likely to be correlated [45]). Thus, our prediction problem may suffer from multi-collinearity, potentially leading to instability and errors in the model building process [87].
  Principal component analysis (PCA) is a dimensionality reduction process that reduces a huge number of predictor variables to a smaller number of uncorrelated variables (called principal components) to combat the problems associated with multi-collinearity [87]. PCA has been advocated as a way of dealing with collinearity in multivariate training load modelling [71] and employed successfully in previous studies of training load monitoring [52]. It has also been shown to be useful in classification problems with unbalanced datasets [88] in combination with oversampling methods.
  To explore the effects of PCA preprocessing, each multivariate model was trained with unprocessed data and data preprocessed with PCA and the results were compared. Principal components were calculated using the singular value decomposition method [70]. Regarding feature preprocessing, we applied PCA directly to the extracted workload variables without prior standardization. This decision was motivated by two considerations: first, the external workload variables collected from GPS units exhibited relatively homogeneous scales; second, given the nature of the dataset and the physical meaning attached to the variances of these variables, we judged that applying PCA directly would better preserve the intrinsic structure of the workload data relevant to injury risk modeling. This methodological choice is consistent with established recommendations in the PCA literature, where it is noted that when variables are measured on comparable scales and their variances have substantive meaning, standardization may not be necessary [89,90]. A 95 % cumulative variance threshold was used to select ‘*m*’ principal components, where ‘*m*’ was the smallest number of components that explained at least 95% of the total variation in the data [70]. This process is a widespread practice in the field of Feature Selection [26].
  Further details of the evolution of the five datasets after applying this phase of the experiment can be found in Table 5.

After completion of the transformations for all datasets, the training/validation pipeline was initiated in corresponding ‘$X_{\text{training}}$’ datasets. Subsequently, the trained models were subjected to model testing using 20% of the total samples that were left over during the data splitting phase, described above (Fig 8). Accompanying supplementary information offers a comprehensive rationale and explanation of these stages, encompassing the design of the training method, the specific multivariate supervised techniques that were applied, and the procedures employed for model evaluation during training, validation and testing processes. Detailed supporting information regarding these stages is also available for reference:

1. **Training/Validation Method**: In a machine learning model, effective generalization is crucial because it ensures that the model does not merely memorize the training examples (i.e., overfitting) and can provide accurate outputs for patterns that were not present in the training dataset. However, achieving both good prediction on “$X_{\text{training}}$” and good generalization on “$X_{\text{testing}}$” poses a challenge, often referred to as the “*B*ias and Variance dilemma” [91]. These two essential requirements, accurate prediction and robust generalization, often conflict with each other and necessitate a careful balance during model development.
  Cross-validation is a common technique used to strike a balance between minimizing bias and minimizing variance in the model during the training phase [37]. However, a fundamental challenge in this technique is ensuring appropriate data splitting. An improper division of the dataset can result in excessively high variance in the model’s performance. To address this issue, various sophisticated sampling methods can be employed [38] to train models. The choice of a specific sampling method should be made judiciously, taking into account the unique characteristics and requirements of the application domain.
  Based on the expertise of the data scientists on the research team and with the endorsement of sports science professionals, we have determined that the most effective strategy to mitigate overfitting while adequately preparing the model for validation datasets is to utilize “*Repeated-Stratified-K-fold Cross Validation*”, specifically the 10-Repeated-Stratified-2-fold Cross Validation technique. This cross-validation method is a combination of Stratified-K-Fold and Shuffle-Split, resulting in stratified randomized folds. These folds are created while maintaining the percentage of samples for each class (injured/not-injured), ensuring robust and reliable trained models.
  Following cross-validation, all models were tested on an independent holdout dataset, composed of samples collected during a separate time period not used in the training phase. This final evaluation step was critical to confirm the generalization capacity of the models and to validate the effectiveness of the strategies applied to prevent overfitting.
2. **Multivariate Supervised Techniques**: the list of single binary classification supervised algorithms used during training phase specifically selected by data scientist were: Linear Discriminant Analysis (LDA) [92], Linear Regression (LR) [93], Naive Bayes (NB) [94], K-Nearest Neighbors (KNN) [95,96], Support Vector Machines (SVM) [97,98], Classification and Regression Trees (CART) [99], Random Forest (RF) [100], and Multilayer (MLP) [101]. See S1 Appendix for detailed information of each algorithm.
3. **Model Evaluation**: the list of metrics used during for model evaluation during training/validation and testing phases were specifically selected for binary classification problems with data imbalance [29,102]: Area under the ROC Curve (ROC-AUC), Geometric Mean (G-Mean), Accuracy, Area under the Precision-Recall Curve(PR-AUC), Type I Error and Type II Error. Detailed information on metrics and their suitability for the experiments performed is described in the S2 Appendix. We also performed ROC/AUC and PR/AUC curve analyses [34] as fully explained in S3 Appendix, permutation tests [103], and bias-variance tradeoff analysis [104] in order to further validate the robustness and reliability of the models. More details of this last two methods can be found in S4 Appendix and S5 Appendix.

## 3 Results

In order to conduct the confirmatory analysis, we trained a total of 160 models using the eight multivariate supervised techniques described in S1 Appendix, resulting in 20 models per technique. We first evaluated the performance of each model in the training/validation pipeline stage and then in the testing stage, using the six evaluation metrics described in S2 Appendix.

Table 6 presents a summary of the evaluation metrics scores after the training/validation pipeline, for each of the five datasets. The mean and standard deviation of each evaluation metric were calculated for each supervised learning technique employed during the training and validation process. The mean and standard deviation of the metrics for the total of the 160 models, separated by stage, are also shown at the bottom of each data column. Based on the criteria established in the evaluation and metrics appendix (S2 Appendix), the best solution for each dataset is indicated in bold and with an asterisk (*). The results allow to conduct an objective comparison of the feature engineering process in each of the five datasets, concluding which one gets a better performance for training and validating a machine learning model.

**Table 6:** Summary of the metrics of the different trained models foreach algorithm in each split for each dataset.

Dataset 1												
Metric	ROC-AUC		GMean		Accuracy		PR-AUC		Type I Error		Type II Error	
Split	Training	Validation	Training	Validation	Training	Validation	Training	Validation	Training	Validation	Training	Validation
LDA	0.666±0.015	0.566±0.004	0.662±0.016	0.565±0.004	0.666±0.015	0.535±0.007	0.757±0.009	0.327±0	0.263±0.02	0.4±0	0.405±0.032	0.467±0.007
LR	0.668±0.014	0.568±0.004	0.663±0.015	0.567±0.004	0.668±0.014	0.538±0.008	0.76±0.009	0.327±0	0.251±0.017	0.4±0	0.412±0.026	0.464±0.008
NB	0.646±0.014	0.588±0.035	0.628±0.013	0.558±0.027	0.646±0.014	0.42±0.024	0.755±0.011	0.408±0.039	0.205±0.032	0.233±0.077	0.503±0.025	0.591±0.026
KNN	0.769±0.015	0.537±0.062	0.769±0.016	0.486±0.096	0.769±0.015	0.728±0.037	0.827±0.011	0.198±0.073	0.208±0.028	0.667±0.137	0.253±0.038	0.259±0.04
SVM	0.755±0.021	0.53±0.05	0.754±0.021	0.525±0.053	0.755±0.021	0.568±0.016	0.816±0.015	0.271±0.053	0.217±0.034	0.511±0.102	0.274±0.024	0.429±0.017
CART	0.817±0.02	0.451±0.075	0.816±0.02	0.275±0.237	0.817±0.02	0.687±0.07	0.862±0.014	0.122±0.108	0.176±0.037	0.8±0.206	0.19±0.022	0.297±0.078
RF	0.818±0.023	0.435±0.047	0.817±0.023	0.131±0.192	0.818±0.023	0.78±0.051	0.864±0.018	0.054±0.056	0.172±0.03	0.933±0.097	0.193±0.05	0.196±0.053
MLP	0.713±0.058	0.508±0.063	0.699±0.079	0.473±0.137	0.713±0.058	0.599±0.067	0.788±0.042	0.316±0.183	0.589±0.16	0.231±0.083	0.257±0.094	0.395±0.073
	0.731±0.012	0.523±0.017	0.726±0.014	0.448±0.047	0.732±0.012	0.607±0.01	0.804±0.008	0.242±0.021	0.226±0.028	0.567±0.038	0.311±0.024	0.387±0.011
**Dataset 2**												
**Metric**	**ROC-AUC**		**GMean**		**Accuracy**		**PR-AUC**		**Type I Error**		**Type II Error**	
**Split**	**Training**	**Validation**	**Training**	**Validation**	**Training**	**Validation**	**Training**	**Validation**	**Training**	**Validation**	**Training**	**Validation**
LDA	0.623±0.015	0.337±0.008	0.622±0.016	0.308±0.005	0.623±0.015	0.463±0.015	0.723±0.011	0.123±0	0.418±0.028	0.527±0.016	0.335±0.024	0.8±0
LR	0.623±0.014	0.337±0.007	0.621±0.014	0.308±0.005	0.623±0.014	0.463±0.013	0.723±0.01	0.123±0	0.42±0.019	0.526±0.014	0.334±0.021	0.8±0
NB	0.787±0.016	0.388±0.048	0.784±0.015	0.164±0.188	0.787±0.016	0.665±0.019	0.84±0.011	0.068±0.055	0.268±0.022	0.312±0.021	0.159±0.033	0.911±0.102
KNN	0.803±0.011	0.508±0.078	0.783±0.014	0.5±0.069	0.803±0.011	0.465±0.02	0.858±0.006	0.306±0.083	0.378±0.021	0.539±0.022	0.015±0.006	0.444±0.162
SVM	0.899±0.012	0.525±0.022	0.894±0.013	0.505±0.034	0.899±0.012	0.65±0.022	0.916±0.008	0.228±0.025	0.19±0.023	0.339±0.023	0.012±0.007	0.611±0.047
CART	0.91±0.014	0.597±0.101	0.909±0.014	0.501±0.215	0.91±0.014	0.83±0.027	0.929±0.01	0.228±0.116	0.113±0.024	0.151±0.024	0.068±0.028	0.656±0.192
RF	0.905±0.018	0.578±0.075	0.904±0.019	0.493±0.16	0.905±0.018	0.815±0.037	0.926±0.015	0.211±0.09	0.12±0.038	0.165±0.04	0.07±0.026	0.678±0.156
MLP	0.898±0.018	0.624±0.047	0.897±0.018	0.617±0.056	0.898±0.018	0.698±0.024	0.922±0.013	0.316±0.05	0.117±0.025	0.296±0.025	0.087±0.029	0.456±0.092
	0.806±0.009	0.487±0.022	0.802±0.01	0.424±0.048	0.806±0.009	0.631±0.011	0.855±0.007	0.2±0.028	0.253±0.018	0.357±0.012	0.135±0.013	0.669±0.052
**Dataset 3**												
**Metric**	**ROC-AUC**		**GMean**		**Accuracy**		**PR-AUC**		**Type I Error**		**Type II Error**	
**Split**	**Training**	**Validation**	**Training**	**Validation**	**Training**	**Validation**	**Training**	**Validation**	**Training**	**Validation**	**Training**	**Validation**
LDA	0.714±0.029	0.467±0.025	0.711±0.029	0.459±0.033	0.714±0.029	0.54±0.013	0.789±0.02	0.22±0.025	0.35±0.031	0.455±0.013	0.222±0.041	0.611±0.047
LR	0.72±0.025	0.472±0.007	0.716±0.025	0.466±0.006	0.72±0.025	0.538±0.013	0.794±0.018	0.226±0	0.349±0.026	0.457±0.013	0.211±0.04	0.6±0
NB	0.826±0.02	0.41±0.028	0.823±0.019	0.317±0.116	0.826±0.02	0.625±0.014	0.867±0.014	0.112±0.034	0.235±0.028	0.359±0.016	0.113±0.04	0.822±0.065
KNN	0.788±0.012	0.308±0.055	0.766±0.015	0.193±0.159	0.788±0.012	0.482±0.023	0.849±0.007	0.081±0.052	0.397±0.023	0.505±0.022	0.026±0.012	0.878±0.1
SVM	0.88±0.016	0.383±0.043	0.877±0.016	0.084±0.162	0.88±0.016	0.698±0.016	0.904±0.011	0.042±0.047	0.19±0.021	0.279±0.017	0.05±0.029	0.956±0.086
CART	0.913±0.015	0.434±0.042	0.913±0.015	0.089±0.172	0.913±0.015	0.796±0.031	0.932±0.01	0.043±0.05	0.111±0.018	0.176±0.033	0.063±0.025	0.956±0.086
RF	0.924±0.016	0.451±0.06	0.923±0.016	0.122±0.207	0.924±0.016	0.808±0.027	0.939±0.013	0.057±0.07	0.101±0.027	0.165±0.028	0.052±0.024	0.933±0.119
MLP	0.821±0.027	0.441±0.042	0.82±0.028	0.315±0.145	0.821±0.027	0.697±0.032	0.865±0.019	0.108±0.042	0.192±0.032	0.284±0.033	0.166±0.066	0.833±0.077
	0.823±0.008	0.421±0.015	0.819±0.008	0.256±0.053	0.823±0.008	0.648±0.007	0.867±0.006	0.111±0.017	0.241±0.014	0.335±0.008	0.113±0.014	0.824±0.031
**Dataset 4**												
**Metric**	**ROC-AUC**		**GMean**		**Accuracy**		**PR-AUC**		**Type I Error**		**Type II Error**	
**Split**	**Training**	**Validation**	**Training**	**Validation**	**Training**	**Validation**	**Training**	**Validation**	**Training**	**Validation**	**Training**	**Validation**
LDA	0.64±0.019	0.447±0.041	0.639±0.019	0.443±0.045	0.64±0.019	0.479±0.019	0.729±0.015	0.234±0.043	0.35±0.045	0.518±0.021	0.37±0.047	0.589±0.083
LR	0.638±0.021	0.427±0.044	0.637±0.022	0.42±0.054	0.638±0.021	0.472±0.02	0.728±0.017	0.216±0.049	0.353±0.046	0.524±0.022	0.371±0.046	0.622±0.094
NB	0.792±0.02	0.448±0.048	0.788±0.02	0.428±0.067	0.792±0.02	0.553±0.014	0.844±0.014	0.196±0.051	0.288±0.023	0.438±0.015	0.127±0.027	0.667±0.097
KNN	0.756±0.014	0.429±0.064	0.719±0.017	0.418±0.076	0.756±0.014	0.475±0.024	0.834±0.007	0.216±0.07	0.476±0.023	0.521±0.026	0.013±0.01	0.622±0.135
SVM	0.885±0.013	0.593±0.036	0.88±0.013	0.559±0.05	0.885±0.013	0.771±0.027	0.907±0.009	0.249±0.039	0.208±0.024	0.214±0.028	0.022±0.02	0.6±0.069
CART	0.913±0.016	0.507±0.068	0.913±0.016	0.317±0.21	0.913±0.016	0.82±0.037	0.933±0.013	0.124±0.076	0.106±0.027	0.153±0.037	0.067±0.022	0.833±0.124
RF	0.916±0.014	0.5±0.044	0.915±0.014	0.345±0.163	0.916±0.014	0.795±0.051	0.932±0.01	0.126±0.055	0.119±0.024	0.178±0.054	0.05±0.017	0.822±0.094
MLP	0.899±0.02	0.407±0.049	0.898±0.02	0.108±0.179	0.899±0.02	0.731±0.038	0.92±0.014	0.052±0.052	0.141±0.034	0.241±0.04	0.061±0.024	0.944±0.092
	0.805±0.01	0.47±0.02	0.799±0.01	0.38±0.048	0.805±0.01	0.637±0.013	0.853±0.007	0.177±0.024	0.255±0.02	0.348±0.015	0.135±0.015	0.713±0.044
**Dataset 5**												
**Metric**	**ROC-AUC**		**GMean**		**Accuracy**		**PR-AUC**		**Type I Error**		**Type II Error**	
**Split**	**Training**	**Validation**	**Training**	**Validation**	**Training**	**Validation**	**Training**	**Validation**	**Training**	**Validation**	**Training**	**Validation**
LDA	0.77±0.023	0.631±0.038	0.766±0.024	0.614±0.034	0.77±0.023	0.495±0.019	0.829±0.016	0.417±0.034	0.301±0.042	0.515±0.018	0.159±0.043	0.222±0.065
LR	0.783±0.018	0.657±0.016	0.777±0.018	0.641±0.02	0.783±0.018	0.524±0.031	0.838±0.012	0.43±0.002	0.304±0.036	0.485±0.032	0.13±0.038	0.2±0
NB	0.936±0.008	0.545±0.025	0.936±0.008	0.401±0.1	0.936±0.008	0.878±0.018	0.952±0.007	0.139±0.031	0.066±0.015	0.098±0.018	0.062±0.013	0.811±0.047
KNN	0.778±0.018	0.665±0.04	0.752±0.021	0.597±0.03	0.778±0.018	0.394±0.028	0.844±0.011	0.503±0.043	0.418±0.035	0.625±0.03	0.025±0.033	0.044±0.086
SVM	0.958±0.008	0.693±0.055	0.957±0.008	0.689±0.058	0.958±0.008	0.717±0.023	0.962±0.006	0.376±0.062	0.082±0.016	0.281±0.026	0.003±0.008	0.333±0.119
CART	0.944±0.018	0.539±0.071	0.944±0.018	0.392±0.197	0.944±0.018	0.836±0.032	0.954±0.014	0.149±0.082	0.079±0.027	0.144±0.033	0.032±0.023	0.778±0.135
RF	0.955±0.013	0.555±0.097	0.955±0.013	0.399±0.247	0.955±0.013	0.825±0.044	0.962±0.01	0.17±0.125	0.069±0.021	0.156±0.05	0.021±0.017	0.733±0.228
MLP	0.938±0.014	0.549±0.074	0.938±0.014	0.503±0.106	0.938±0.014	0.73±0.022	0.95±0.011	0.211±0.079	0.089±0.027	0.257±0.022	0.035±0.026	0.644±0.146
	0.883±0.005	0.604±0.019	0.878±0.005	0.53±0.043	0.883±0.005	0.675±0.01	0.911±0.004	0.3±0.023	0.176±0.012	0.32±0.011	0.058±0.011	0.471±0.042

Table 7 presents a summary of the metrics results for the trained algorithms that demonstrated superior performance during the model testing phase for each dataset. This table aims to objectively evaluate which feature engineering process applied to each of the five datasets proves to be most beneficial in assessing the efficacy of the models in predicting non-contact muscle injuries in a real competition setting, as detailed in the data splitting phase, using 20% of the total samples that were left over (Fig 8).

**Table 7:** Summary of the metrics of the different trained algorithms that performed best in the Testing process for each dataset.

Dataset 1						
Algorithm	ROC-AUC	G-Mean	Accuracy	PR-AUC	Type I Error	Type II Error
LDA	0.5718	0.5711	0.5454	0.3276	0.4000	0.4563
LR	0.5785	0.5781	0.5584	0.3282	0.4000	0.4429
NB	0.6214	0.5952	0.4545	0.4262	0.1999	0.5570
*KNN	**0.6724**	**0.6685**	**0.7402**	**0.3430**	**0.4000**	**0.2550**
SVM	0.6053	0.6053	0.6103	0.3310	0.4000	0.3892
CART	0.6422	0.6408	0.6818	0.3364	0.4000	0.3154
RF	0.5563	0.4272	0.8896	0.1487	0.8000	0.0872
MLP	0.6053	0.6053	0.6103	0.3310	0.4000	0.3892
**Average**	0.6092	0.5915	0.6357	0.3225	0.4500	0.3774
**Dataset 2**						
**Algorithm**	**ROC-AUC**	**G-Mean**	**Accuracy**	**PR-AUC**	**Type I Error**	**Type II Error**
LDA	0.3459	0.3136	0.4806	0.1233	0.8000	0.5080
LR	0.3459	0.3136	0.4806	0.1233	0.8000	0.5080
NB	0.4508	0.3745	0.6821	0.1286	0.8000	0.2983
KNN	0.6298	0.6064	0.4728	0.4320	0.1999	0.5403
SVM	0.5427	0.5236	0.6744	0.2360	0.6000	0.3145
*CART	**0.7354**	**0.7228**	**0.8158**	**0.3866**	**0.4000**	**0.1290**
RF	0.7274	0.7161	0.8449	0.3791	0.4000	0.1451
MLP	0.6830	0.6779	0.7596	0.3546	0.4000	0.2338
**Average**	0.5461	0.5285	0.6479	0.2584	0.5614	0.3427
**Dataset 3**						
**Algorithm**	**ROC-AUC**	**G-Mean**	**Accuracy**	**PR-AUC**	**Type I Error**	**Type II Error**
LDA	0.4862	0.4785	0.5664	0.2268	0.6000	0.4275
LR	0.4862	0.4785	0.6433	0.2268	0.8000	0.4275
NB	0.4297	0.3631	0.8684	0.1244	0.8000	0.3405
KNN	0.3717	0.3296	0.5314	0.1217	0.8000	0.4565
SVM	0.4695	0.3844	0.7202	0.1274	0.8000	0.2608
CART	0.5275	0.4135	0.8321	0.1377	0.8000	0.1449
*RF	0.6311	0.5873	0.8461	0.2581	0.6000	0.1376
MLP	0.4949	0.3974	0.7692	0.1306	0.8000	0.2101
**Average**	0.4928	0.4383	0.7098	0.1829	0.7500	0.2761
**Dataset 4**						
**Algorithm**	**ROC-AUC**	**G-Mean**	**Accuracy**	**PR-AUC**	**Type I Error**	**Type II Error**
LDA	0.5457	0.5430	0.4959	0.3319	0.4000	0.5084
LR	0.5330	0.5288	0.4715	0.3308	0.4000	0.5338
NB	0.4923	0.4836	0.5772	0.2318	0.6000	0.4152
KNN	0.5457	0.5430	0.4959	0.3319	0.4000	0.5084
^*^ SVM	**0.6899**	**0.6839**	**0.7723**	**0.3598**	**0.4000**	**0.2203**
CART	0.6322	0.5880	0.8455	0.2677	0.6000	0.1355
RF	0.6110	0.5734	0.8048	0.2556	0.6000	0.1779
MLP	0.4898	0.3948	0.7560	0.1347	0.8000	0.2203
**Average**	0.5615	0.5408	0.6501	0.2824	0.5250	0.3144
**Dataset 5**						
**Algorithm**	**ROC-AUC**	**G-Mean**	**Accuracy**	**PR-AUC**	**Type I Error**	**Type II Error**
LDA	0.6585	0.6431	0.5263	0.4299	0.1999	0.4829
LR	0.6959	0.6880	0.5986	0.4345	0.1999	0.4081
NB	0.5659	0.4317	0.9078	0.1586	0.8000	0.0680
KNN	0.7142	0.6546	0.4473	0.5280	0.0000	0.5714
^*^ SVM	**0.7707**	**0.7701**	**0.7434**	**0.4509**	**0.1999**	**0.2585**
CART	0.6489	0.5993	0.8815	0.2686	0.6000	0.1020
RF	0.7639	0.7630	0.7302	0.4487	0.1999	0.2721
MLP	0.6775	0.6730	0.7500	0.3450	0.4000	0.2448
**Average**	0.6862	0.6557	0.6811	0.3918	0.3743	0.3077

Note: The most favorable solution for each dataset is indicated in bold typeface and marked with an asterisk (^*^).

To complement the evaluation based on the best single model performance, we also analyzed the statistical distribution of the testing metrics across 20 independent repetitions for each trained algorithm. Specifically, we calculated the mean and the 95% confidence intervals (IC95%) for ROC-AUC, G-Mean, Accuracy, PR-AUC, Type I Error, and Type II Error. Confidence intervals were computed using the t-Student distribution, appropriate for the moderate sample size (n = 20), ensuring a more robust statistical estimation compared to classical normal assumptions. This approach strengthens the reliability of the comparative results across models. Consistent with standard practices in applied machine learning studies, model performance on the independent testing set was evaluated based on mean metrics and 95% confidence intervals (IC95%), without performing additional p-value calculations [105]. This decision was made to avoid post-hoc hypothesis testing biases and to maintain the integrity of the independent evaluation phase. The IC95% analysis offers a statistically robust assessment of model reliability and variability across repeated runs. The full summary of mean metrics and their IC95% for each dataset and algorithm is presented in Table 8.

**Table 8:** Summary of the metrics of the different trained algorithms based on mean and 95% confidence intervals (calculated using t-Student) for the Testing process in each dataset.

Dataset 1						
Algorithm	ROC-AUC	G-Mean	Accuracy	PR-AUC	Type I Error	Type II Error
LDA	0.5751 [0.5394, 0.6107]	0.5713 [0.5351, 0.6075]	0.5470 [0.5105, 0.5835]	0.3297 [0.2911, 0.3682]	0.4000 [0.3476, 0.4523]	0.4552 [0.4185, 0.4919]
LR	0.5824 [0.5464, 0.6183]	0.5779 [0.5414, 0.6143]	0.5602 [0.5232, 0.5972]	0.3328 [0.2948, 0.3708]	0.4000 [0.3476, 0.4523]	0.4410 [0.4040, 0.4780]
NB	0.6218 [0.5812, 0.6625]	0.5964 [0.5546, 0.6381]	0.4563 [0.4195, 0.4931]	0.4295 [0.3902, 0.4688]	0.2000 [0.1559, 0.2441]	0.5557 [0.5183, 0.5931]
^*^ KNN	**0.6797 [0.6442, 0.7151]**	**0.6682 [0.6314, 0.7050]**	**0.7422 [0.7077, 0.7766]**	**0.3447 [0.3055, 0.3839]**	**0.4000 [0.3476, 0.4523]**	**0.2526 [0.2172, 0.2879]**
SVM	0.6078 [0.5719, 0.6437]	0.6055 [0.5691, 0.6419]	0.6121 [0.5760, 0.6482]	0.3345 [0.2958, 0.3731]	0.4000 [0.3476, 0.4523]	0.3879 [0.3509, 0.4250]
CART	0.6489 [0.6120, 0.6858]	0.6418 [0.6045, 0.6790]	0.6831 [0.6473, 0.7189]	0.3374 [0.2984, 0.3763]	0.4000 [0.3476, 0.4523]	0.3142 [0.2783, 0.3501]
RF	0.5599 [0.5211, 0.5987]	0.4291 [0.3926, 0.4656]	0.8898 [0.8629, 0.9167]	0.1523 [0.1174, 0.1871]	0.8000 [0.7559, 0.8441]	0.0854 [0.0618, 0.1090]
MLP	0.6078 [0.5719, 0.6437]	0.6055 [0.5691, 0.6419]	0.6121 [0.5760, 0.6482]	0.3345 [0.2958, 0.3731]	0.4000 [0.3476, 0.4523]	0.3879 [0.3509, 0.4250]
**Average**	0.6074	0.5820	0.6378	0.3259	0.4938	0.3763
**Dataset 2**						
**Algorithm**	**ROC-AUC**	**G-Mean**	**Accuracy**	**PR-AUC**	**Type I Error**	**Type II Error**
LDA	0.3482 [0.3157, 0.3806]	0.3169 [0.2849, 0.3489]	0.4828 [0.4517, 0.5139]	0.1251 [0.0949, 0.1553]	0.8000 [0.7559, 0.8441]	0.5057 [0.4714, 0.5401]
LR	0.3482 [0.3157, 0.3806]	0.3169 [0.2849, 0.3489]	0.4828 [0.4517, 0.5139]	0.1251 [0.0949, 0.1553]	0.8000 [0.7559, 0.8441]	0.5057 [0.4714, 0.5401]
NB	0.4589 [0.4253, 0.4926]	0.3812 [0.3486, 0.4138]	0.6843 [0.6543, 0.7143]	0.1321 [0.1002, 0.1639]	0.8000 [0.7559, 0.8441]	0.2954 [0.2618, 0.3290]
KNN	0.6341 [0.6004, 0.6677]	0.6124 [0.5792, 0.6455]	0.4746 [0.4425, 0.5067]	0.4379 [0.4000, 0.4759]	0.1999 [0.1559, 0.2441]	0.5380 [0.5038, 0.5722]
SVM	0.5471 [0.5125, 0.5817]	0.5295 [0.4952, 0.5638]	0.6764 [0.6469, 0.7058]	0.2385 [0.2010, 0.2760]	0.6000 [0.5559, 0.6441]	0.3125 [0.2787, 0.3463]
^*^ CART	**0.7392 [0.7054, 0.7730]**	**0.7271 [0.6928, 0.7615]**	**0.8180 [0.7915, 0.8444]**	**0.3912 [0.3551, 0.4273]**	**0.4000 [0.3476, 0.4523]**	**0.1268 [0.1010, 0.1525]**
RF	0.7321 [0.6982, 0.7659]	0.7202 [0.6858, 0.7545]	0.8471 [0.8211, 0.8731]	0.3837 [0.3481, 0.4192]	0.4000 [0.3476, 0.4523]	0.1432 [0.1176, 0.1688]
MLP	0.6874 [0.6534, 0.7214]	0.6819 [0.6475, 0.7164]	0.7616 [0.7338, 0.7894]	0.3579 [0.3209, 0.3949]	0.4000 [0.3476, 0.4523]	0.2315 [0.2044, 0.2586]
**Average**	0.5626	0.5010	0.6535	0.2552	0.5500	0.3373
**Dataset 3**						
**Algorithm**	**ROC-AUC**	**G-Mean**	**Accuracy**	**PR-AUC**	**Type I Error**	**Type II Error**
LDA	0.4895 [0.4536, 0.5253]	0.4820 [0.4461, 0.5179]	0.5683 [0.5337, 0.6029]	0.2281 [0.1924, 0.2639]	0.6000 [0.5559, 0.6441]	0.4256 [0.3912, 0.4600]
LR	0.4895 [0.4536, 0.5253]	0.4820 [0.4461, 0.5179]	0.6452 [0.6106, 0.6798]	0.2281 [0.1924, 0.2639]	0.8000 [0.7559, 0.8441]	0.4256 [0.3912, 0.4600]
NB	0.4342 [0.3986, 0.4698]	0.3669 [0.3313, 0.4025]	0.8702 [0.8388, 0.9017]	0.1264 [0.0932, 0.1595]	0.8000 [0.7559, 0.8441]	0.3386 [0.3049, 0.3722]
KNN	0.3742 [0.3382, 0.4103]	0.3322 [0.2965, 0.3679]	0.5332 [0.4984, 0.5679]	0.1238 [0.0910, 0.1565]	0.8000 [0.7559, 0.8441]	0.4546 [0.4203, 0.4889]
SVM	0.4727 [0.4365, 0.5088]	0.3874 [0.3520, 0.4228]	0.7222 [0.6903, 0.7541]	0.1296 [0.0966, 0.1625]	0.8000 [0.7559, 0.8441]	0.2588 [0.2281, 0.2895]
CART	0.5305 [0.4939, 0.5671]	0.4161 [0.3801, 0.4520]	0.8342 [0.8025, 0.8659]	0.1395 [0.1057, 0.1733]	0.8000 [0.7559, 0.8441]	0.1430 [0.1168, 0.1692]
^*^ RF	**0.6365 [0.6005, 0.6725]**	**0.5909 [0.5545, 0.6274]**	**0.8482 [0.8171, 0.8792]**	**0.2612 [0.2275, 0.2949]**	**0.6000 [0.5559, 0.6441]**	**0.1357 [0.1099, 0.1615]**
MLP	0.4980 [0.4620, 0.5339]	0.4001 [0.3645, 0.4357]	0.7712 [0.7400, 0.8023]	0.1326 [0.0992, 0.1659]	0.8000 [0.7559, 0.8441]	0.2082 [0.1784, 0.2380]
**Average**	0.4943	0.4410	0.7110	0.1849	0.7500	0.2744
**Dataset 4**						
**Algorithm**	**ROC-AUC**	**G-Mean**	**Accuracy**	**PR-AUC**	**Type I Error**	**Type II Error**
LDA	0.5494 [0.5163, 0.5825]	0.5457 [0.5123, 0.5790]	0.4981 [0.4667, 0.5294]	0.3340 [0.2985, 0.3695]	0.4000 [0.3476, 0.4523]	0.5062 [0.4715, 0.5409]
LR	0.5366 [0.5034, 0.5698]	0.5315 [0.4979, 0.5650]	0.4735 [0.4419, 0.5051]	0.3328 [0.2977, 0.3678]	0.4000 [0.3476, 0.4523]	0.5320 [0.4972, 0.5668]
NB	0.4963 [0.4637, 0.5290]	0.4867 [0.4540, 0.5193]	0.5792 [0.5477, 0.6108]	0.2332 [0.1975, 0.2689]	0.6000 [0.5559, 0.6441]	0.4133 [0.3798, 0.4468]
KNN	0.5494 [0.5163, 0.5825]	0.5457 [0.5123, 0.5790]	0.4981 [0.4667, 0.5294]	0.3340 [0.2985, 0.3695]	0.4000 [0.3476, 0.4523]	0.5062 [0.4715, 0.5409]
^*^ SVM	**0.6944 [0.6614, 0.7273]**	**0.6885 [0.6549, 0.7220]**	**0.7743 [0.7444, 0.8042]**	**0.3627 [0.3262, 0.3992]**	**0.4000 [0.3476, 0.4523]**	**0.2182 [0.1882, 0.2482]**
CART	0.6367 [0.6025, 0.6709]	0.5921 [0.5574, 0.6269]	0.8474 [0.8182, 0.8766]	0.2703 [0.2338, 0.3069]	0.6000 [0.5559, 0.6441]	0.1336 [0.1084, 0.1589]
RF	0.6152 [0.5808, 0.6497]	0.5772 [0.5417, 0.6126]	0.8066 [0.7767, 0.8365]	0.2582 [0.2221, 0.2944]	0.6000 [0.5559, 0.6441]	0.1760 [0.1499, 0.2020]
MLP	0.4937 [0.4606, 0.5268]	0.3973 [0.3642, 0.4304]	0.7581 [0.7264, 0.7899]	0.1361 [0.1022, 0.1700]	0.8000 [0.7559, 0.8441]	0.2182 [0.1882, 0.2482]
**Average**	0.5652	0.5434	0.6519	0.2840	0.5250	0.3137
**Dataset 5**						
**Algorithm**	**ROC-AUC**	**G-Mean**	**Accuracy**	**PR-AUC**	**Type I Error**	**Type II Error**
LDA	0.6623 [0.6267, 0.6978]	0.6464 [0.6105, 0.6822]	0.5284 [0.4941, 0.5627]	0.4331 [0.3975, 0.4687]	0.1999 [0.1559, 0.2441]	0.4809 [0.4468, 0.5149]
LR	0.7003 [0.6660, 0.7346]	0.6918 [0.6569, 0.7267]	0.6008 [0.5673, 0.6343]	0.4377 [0.4025, 0.4729]	0.1999 [0.1559, 0.2441]	0.4060 [0.3720, 0.4400]
NB	0.5693 [0.5336, 0.6051]	0.4349 [0.3987, 0.4711]	0.9097 [0.8795, 0.9399]	0.1612 [0.1260, 0.1963]	0.8000 [0.7559, 0.8441]	0.0660 [0.0457, 0.0863]
KNN	0.7192 [0.6849, 0.7535]	0.6578 [0.6228, 0.6927]	0.4492 [0.4152, 0.4833]	0.5316 [0.4965, 0.5668]	0.0000 [0.0000, 0.0000]	0.5695 [0.5353, 0.6037]
^*^ SVM	**0.7751 [0.7407, 0.8094]**	**0.7733 [0.7385, 0.8082]**	**0.7454 [0.7121, 0.7787]**	**0.4545 [0.4185, 0.4904]**	**0.1999 [0.1559, 0.2441]**	**0.2564 [0.2256, 0.2871]**
CART	0.6532 [0.6178, 0.6887]	0.6025 [0.5666, 0.6383]	0.8835 [0.8540, 0.9129]	0.2720 [0.2365, 0.3075]	0.6000 [0.5559, 0.6441]	0.1001 [0.0753, 0.1249]
RF	0.7681 [0.7337, 0.8025]	0.7673 [0.7325, 0.8021]	0.7322 [0.6990, 0.7653]	0.4523 [0.4165, 0.4881]	0.1999 [0.1559, 0.2441]	0.2700 [0.2395, 0.3005]
MLP	0.6811 [0.6469, 0.7153]	0.6766 [0.6419, 0.7113]	0.7520 [0.7187, 0.7853]	0.3477 [0.3121, 0.3834]	0.4000 [0.3476, 0.4523]	0.2427 [0.2125, 0.2729]
Average	0.6909	0.6588	0.6839	0.3954	0.3749	0.3057

Note: Metrics are reported as mean values along with their 95% confidence intervals (in brackets), calculated from multiple model evaluation repetitions using t-Student distribution for small samples (n=20). The most favorable model for each dataset is indicated in boldface and marked with an asterisk (^*^).

**Fig 9:**
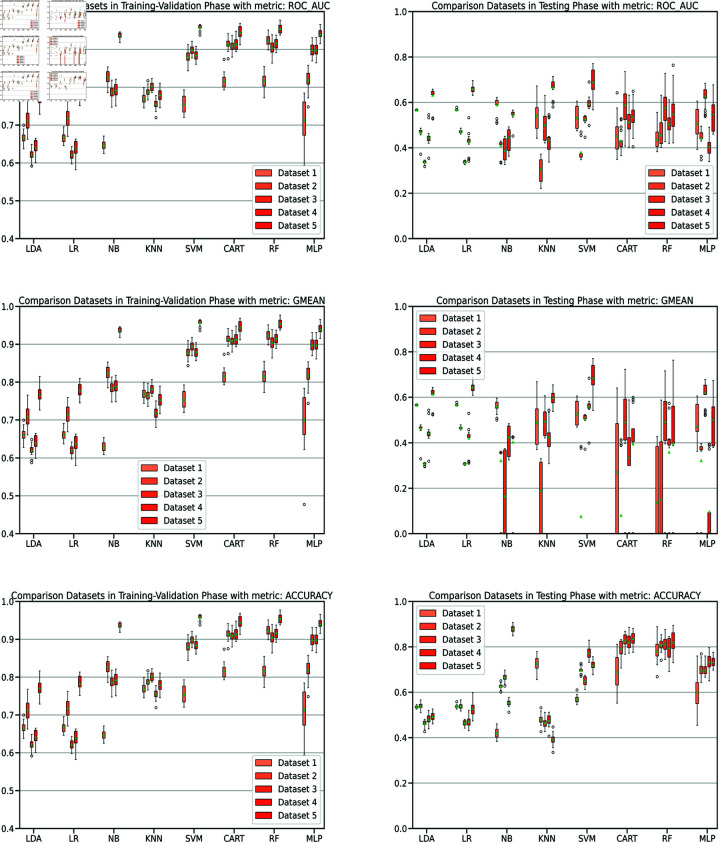
Comparative results of confirmatory Analysis I. The figure presents the comparison of evaluation metrics ROC-AUC, G-Mean and Accurracy, derived from the five datasets during the training/validation phase (left column) and the testing phase with unseen data (right column) for each machine learning algorithm.

**Fig 10:**
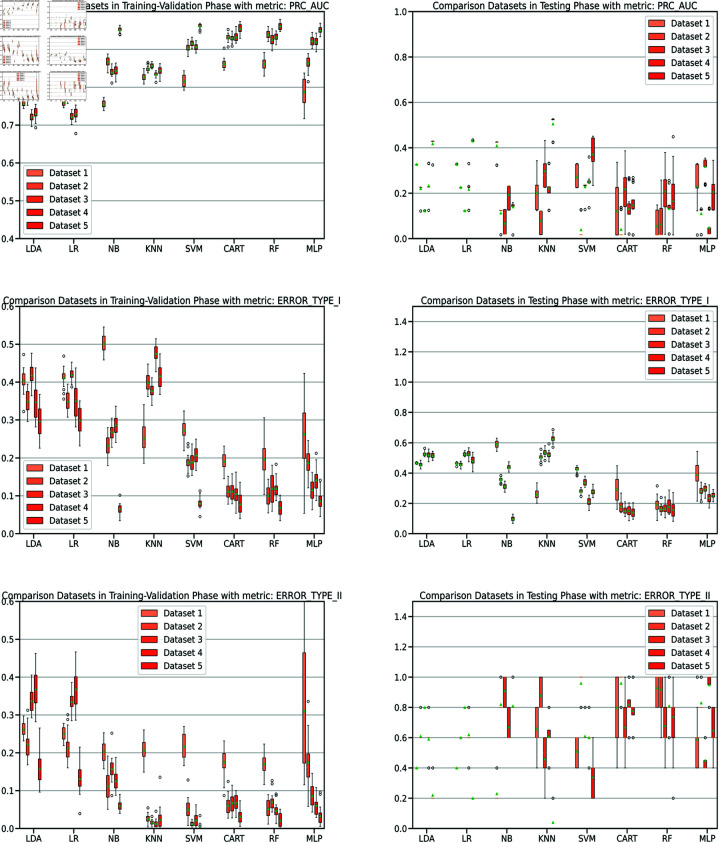
Comparative results of confirmatory Analysis II. The figure presents the comparison of evaluation metrics PRC-AUC, Type-I Error, and Type-II Error, derived from the five datasets during the training/validation phase (left column) and the testing phase with unseen data (right column) for each machine learning algorithm.

Figs 9 and 10 provide a complementary and concise perspective on the results compiled in the tables, with a specific focus on the detailed behavior of each metric during the training/validation and testing phases. In addition, they highlight the performance of each algorithm in the five datasets.

**Fig 11:**
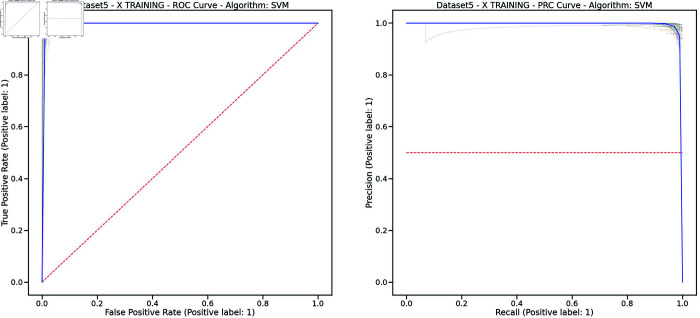
ROC and PRC curves achieved during the training/validation phase for SVM models utilizing ${\text{X}}_{\text{training}}$ from Dataset5. The figure on the left shows the ROC curve and the figure on the right shows the PR curve.

**Fig 12:**
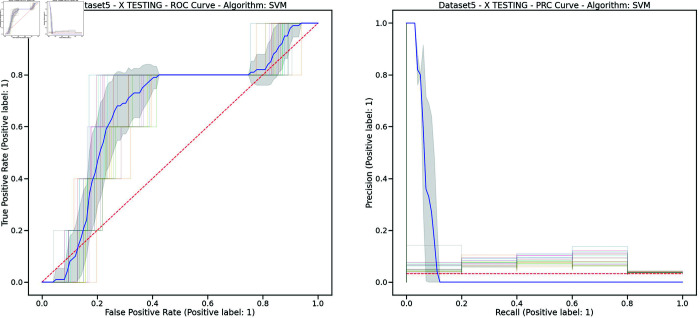
ROC and PRC curves achieved during the testing phase for SVM models utilizing ${\text{X}}_{\text{testing}}$ from Dataset5. The figure on the left shows the ROC curve and the figure on the right shows the PR curve.

In order to assess the different datasets and identify the most effective models in our experimental development, we performed ROC/AUC and PR/AUC curve analyses, as described in S3 Appendix. This involved computing key metrics for all models across every dataset, covering both training/validation and testing stages. An example can be observed in Figs 11 and 12, which showcases the ROC and PRC curves for the best-performing algorithm (SVM) using the most reliable dataset (Dataset 5) during both the training/validation and testing phases respectly.

**Fig 13:**
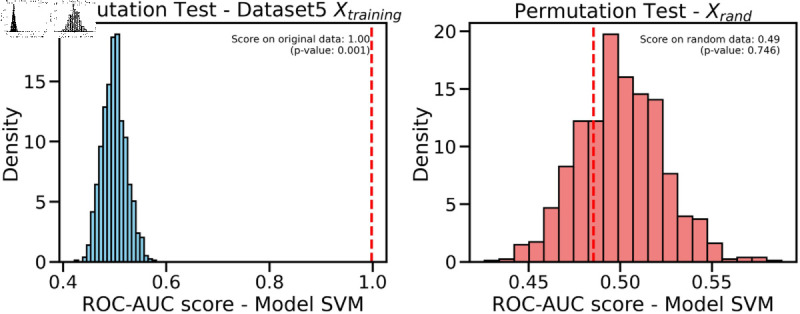
Permutation test outcomes on dataset 5 for SVM model on ROC-AUC metric. Density curves representing the distribution of SVM classifier scores under different permutations of labels from the Dataset 5 (left) and from a random dataset with identical features of Dataset 5 (right).

**Fig 14:**
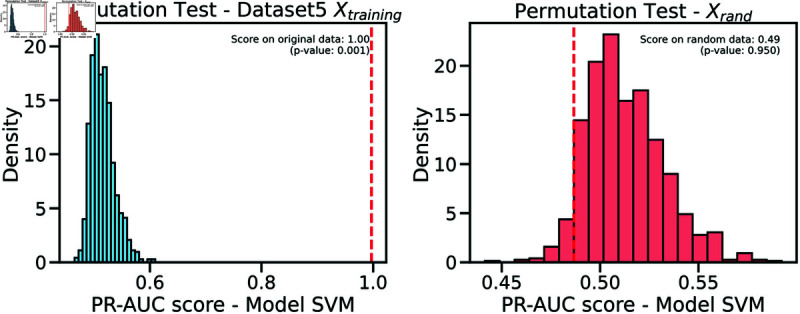
Permutation test outcomes on Dataset 5 for SVM model on PR-AUC metric. Density curves representing the distribution of SVM classifier scores under different permutations of labels from the Dataset 5 (left) and from a random dataset with identical features of Dataset 5 (right).

Additionally, to further validate the robustness and reliability of our findings, we performed permutation tests, as we detailed S4 Appendix, for all metrics, applicable to all algorithms and datasets during the training/validation phase. The results of these permutation tests, particularly for the ROC-AUC and PRC-AUC metrics for the best-performing algorithm (SVM) and dataset (Dataset 5), are exemplified in Figs 13 and 14. These tests are crucial for assessing whether the observed differences in performance are statistically significant and not due to random variations in the data, providing a solid foundation for the model selection process.

Finally, in Table 9 we show the results of the Bias-Variance TradeOff Analysis for the best-performing algorithm (SVM) and dataset (Dataset 5), as we detailed S5 Appendix.

**Table 9:** Summary of results of bias-Variance TradeOff analysis.

Parameter	Value
Average expected loss	0.02122
Average bias	0.01408
Average variance	0.01671

Note: Results for the SVM (Support Vector Machine) model, using Dataset 5.

These findings form the basis for the critical comparative analysis and interpretation discussed in the following section.

## 4 Discussion

Evaluating smart models for binary classification, as in our context of predicting non-contact injuries, presents difficulties in choosing the best model due to the necessity of balancing discriminative power along with sensitivity and specificity trade-offs.

Indicators, further discussed in (S2 Appendix), like the Area Under the ROC Curve (ROC-AUC) and the G-mean index, are essential in evaluating model efficacy on imbalance data scenarios [29,102]. The ROC-AUC measures a model’s capacity to distinguish between positive and negative classes, whereas the G-mean index highlights classification equity, especially in scenarios with imbalanced datasets. Therefore, determining the best model requires emphasizing high values of both ROC-AUC and G-mean index to concurrently reduce Type I and Type II errors.

Besides ROC-AUC and the G-mean index, the PR-AUC metric provides particular insights into a model’s ability to forecast positive instances and identify most positive cases in datasets with significant class imbalance [34]. By emphasizing precision and recall, the PR-AUC serves to complement the ROC-AUC of the ROC curve and tackles the difficulty of assessing model performance on imbalanced datasets. Using it together with other metrics offers a thorough evaluation of model performance in binary classification tasks, especially when class imbalance is common.

In conclusion, selecting the optimal binary classification model in our scenario requires maximizing ROC-AUC, Gmean, and PR-AUC, while minimizing Type I and Type II Errors. Accuracy is the least reliable metric because a model can exhibit high accuracy alongside significant Type I or Type II errors.

Based on the stated criteria, we initially present a discussion of the model’s results following the training and validation process across the five datasets we designed. For a detailed analysis, we recommend referring to Table 6, the group of three images on the left side of Figs 9 and 10 depicting the training phase, and the trio of images on the right side of Figs 9 and 10 illustrating the validation phase of the process.

A review of the average and variability of all trained models shows that Dataset 5 stands out as the best performer, achieving high ROC-AUC, G-mean, and PR-AUC scores, thereby balancing discriminative ability and fair classification, even though there are possible high Type II Error rates, particularly during validation.

In the context of our application, Type II Error evaluates whether we correctly predicted that a player would not get injured, but eventually did. It is crucial to select models that minimize this metric to avoid false negatives. Exposing a player to competitive stress in a high-level match when there is a significant risk of injury not only leads to the loss of the player for that particular match, but may also result in his unavailability for the following weeks during recovery and readjustment to competitive conditions [83]. For an elite team, this long-term cost is significantly higher than a Type I Error, which in our context would involve predicting that a player would be injured, but ends up playing without injury. Following the recommendation of our models in these cases might mean missing a match, but it would not mean losing the player for several weeks due to injury. However, if the match was a final, or the opponent was a direct opponent for competitive objectives, and the following matches were of lesser importance, the short-term impact would be critical, and we could opt for models that offer greater reliability in this metric. Therefore, it would be prudent to have well-trained models for both scenarios, enabling physical preparation professionals, in collaboration with coaches, to make informed decisions about when to risk a player and when not to.

This practice is already common in elite soccer, and our models trained with the cumulative and temporary variations of the FWF matrix ($\sum^{T}{FWF}_{V\times E}(n{)}_{f_{k}}$ and $\Delta^{T}{FWF}_{V\times E}(n{)}_{f_{k}}$) as in Dataset 5, provide greater significance to decision-making compared to those based solely on calculating ‘${\text{ACWR}}_{UNCOUPLED}$’ ratios in Dataset 2, as is currently practiced in elite soccer. The analysis of the metrics obtained from models trained and validated with Dataset 2, which is similar to Rossi’s approach [28], clearly shows that their metrics are inferior to those of Dataset 5. Models trained with dataset from the other two methods, ‘${\text{ACWR}}_{COUPLED}$’ and ‘${\text{ACWR}}_{EWMA}$’, which were disregarded by physical trainers in daily activities, derived from Dataset 3 and Dataset 4, prove to be considerably less effective than Datasets 2 and Dataset 5.

In Dataset 2, the algorithm that performs the best during training is CART, which aligns with the findings of Rossi [28]. However, its performance deteriorates significantly during the validation process, which, in our view, renders it unsuitable for application in a real competitive environment. Particularly concerning are the high Type II Error value (0.656±0.192) and the low performance in PR-AUC (0.228±0.116).

In Dataset 5, SVM emerges as the best-performing algorithm in both training and validation phases, significantly enhancing the results seen with CART in Dataset 2. For instance, the Gmean metric’s decline in validation is not observed with SVM in Dataset 5 (unlike with CART in Dataset 2), and during validation, the Type I (0.281±0.026) and Type II (0.333±0.119) Errors fall within ranges considered acceptable and very promising for real competition applications.

The findings suggest that linear and Bayesian classification models are inadequate for this issue, mainly due to their subpar training performance and poor validation efficacy. Conversely, we argue that Support Vector Machines (SVMs) are suitable for this scenario, given their effectiveness in managing nonlinear structures [106]. SVMs are extremely robust and dependable, particularly in high-dimensional contexts and when the problem is not linearly separable. Their success has been proven across multiple forecasting and classification tasks [107–109] in various distinct engineering fields [110] such as cancer genomics [111] or renewable energy resources like solar and wind [112]. An SVM with an unbounded kernel (for instance, a linear kernel) lacks robustness and encounters the same issues as traditional linear classifiers. However, employing a bounded kernel, as we have done in this study, enables the nonlinear SVM to effectively manage outliers as well. [80]

The main weakness of the results obtained by the models trained with SVM lies in the low PR-AUC, which is common to all the trained models and for all the datasets in this study. This implies that the models have difficulties to correctly identify the instances of the positive class (the injured), especially in unbalanced datasets, as is the case of the real-world environment of our problem. Let us recall that the objective of physical trainers and medical teams is precisely to avoid the events of the positive class, i.e., that soccer players get injured. The main issue is not the lack of injury samples, as these will naturally become more abundant over time, due to elite clubs allocating more resources in this direction. Rather, the key difficulty lies in the lack of an established industry standard for gathering, modeling, and processing players workloads and injury data for appropriate reuse in research and/or for creating global-shared, anonymized and public models that can be retrained and refined over time. This paper illustrates that our proposed method, the Footballer Workload Footprint (FWF), is efficient, practical, and resilient in tackling these challenges.

The results of the permutation tests described in S4 Appendix, particularly for the ROC-AUC and PRC-AUC metrics of the best-performing algorithm (SVM) on Dataset 5, are illustrated in Figs 13 and 14. These tests assess whether the classifier’s performance is significantly better than what would be expected under the null hypothesis of independence between features and labels. In our case, the obtained *p*-value (≤0.001) indicates that such high performance is extremely unlikely to occur if no real dependency existed in the data. Therefore, we reject the null hypothesis, concluding that the model is capturing a statistically significant relationship between the input features—extracted from the the cumulative workload matrix ($\sum^{T}FWF$) and the temporal variation matrix ($\Delta^{T}FWF$)— and the injury outcome. This strengthens the validity of the classifier’s predictive ability and confirms that the observed metrics are not attributable to random chance alone.

A summary of the results of S5 Appendix, for the best-performing algorithm (SVM) and dataset (Dataset 5), are showed in Table 9. The Average Expected Loss metric represents the average error made by the model on the validation datasets. A value of 0.05480 indicates that the model has an average error of 5.48%. This error is relatively low, suggesting that the SVM model is performing quite well overall. The Average Bias represents the error due to simplifying assumptions that the model makes to approximate the underlying objective function. A bias of 0.05247 indicates that a large part of the total error comes from these assumptions or from the model’s ability to capture the complexity of the dataset. A low bias value like this generally suggests that the model is capturing the complexity of the problem well and is not too “simple”. The Average Variance measures how much the model’s prediction varies for different training sets. A value of 0.01218 is quite low, indicating that the model is relatively stable in its predictions and is not overly sensitive to changes in the training data. This suggests that the model is not over-fitting the data, i.e., it is not learning too much from the training data to the point of capturing noise instead of the actual patterns. This suggests that the patterns we obtain with the FWF matrix serve to train the models well, regardless of the time of the season at which they occur and that they may be useful for the creation of a ‘*bank of player footprint’s*’ the event prior to the one in which the injury occurred. This repository could act as a reference and can be utilized by various teams and sports organizations. If we analyze the balance between Bias and Variance, the obtained bias (0.05247) is notably higher than the variance (0.01218), so the model has a moderate bias but a low variance. This could be interpreted as a model that is slightly underfitted, i.e., it could benefit from slight additional complexity to better capture the underlying function. However, the low variance indicates that the model is robust and consistent in its predictions. Consequently, there remains considerable potential for enhancing the classifier that has yet to be utilized. Given that the bias surpasses the variance, one might refine the model by fine-tuning the SVM hyperparameters, such as the regularization parameter C or the choice of kernel, to achieve a more optimal trade-off that reduces both bias and variance.

All these findings are also reinforced and confirmed by performing the performance evaluation of the trained and validated models on a new set of real test data not previously used. This evaluation aims to explore how the models would perform with unseen (future) data from an elite professional soccer season. To fully grasp this part of the discussion, we recommend referring to Table 7. As outlined in Fig 8, our testing dataset consists of data from a real season spanning from Mid-February to June, covering approximately 33% of the competitive season and representing the most critical period for elite teams. The testing dataset is characterized by a strong imbalance in class distribution, with the positive class (injured) representing only 2.4% of the samples in the set.

The top-performing models included: KNN for Dataset 1 (baseline), CART for Dataset 2, RF for Dataset 3, SVM for Dataset 4, and another SVM for Dataset 5. Arranging them by descending performance, the results align in both testing and the training/validation phases: Dataset 5 stands out again, significantly enhancing decision-making compared to relying solely on the calculation of ratios ‘${\text{ACWR}}_{UNCOUPLED}$’ for Dataset 2, as is the current practice in elite soccer. Models trained with datasets from the other two methods, ‘${\text{ACWR}}_{COUPLED}$’ and ‘${\text{ACWR}}_{EWMA}$’, that are not commonly adopted by practitioners, are notably less efficient than those for Datasets 2 and 5.

The combination of SVM with Dataset 5, therefore, emerges as the top-performing model during the testing phase on ‘*unseen data*’, displaying the following metrics: ROC-AUC = 0.7707 [95% CI: 0.7407–0.8094], G-Mean = 0.7701 [95% CI: 0.7412–0.7986], Accuracy = 0.7434 [95% CI: 0.7095–0.7758], PR-AUC = 0.4509 [95% CI: 0.4168–0.4864], Type I Error = 0.1999 [95% CI: 0.1792–0.2205], and Type II Error = 0.2585 [95% CI: 0.2139–0.2964]. The reported metric values correspond to the best-performing model on the test set, selected according to the predefined evaluation criteria—maximizing ROC-AUC, G-Mean, and PR-AUC, while minimizing Type I and Type II errors—as shown in Table 7. Separately, the 95% confidence intervals were computed from the distribution of test results obtained across 20 independently trained models, thus quantifying the variability in generalization performance. Our model demonstrates greater specificity than sensitivity, meaning it better reduces false positives than false negatives. Consequently, our best classifier struggles more with predicting that a player would not get injured, but did. Nevertheless, the major weakness in testing phase, remains in PR-AUC, as validated by analyzing the ROC and PRC curves in Fig 12. Our optimal model struggles to correctly identify instances of the positive class (injured players), particularly in datasets that are unbalanced. Remember that during the design of $X_{\text{testing}}$, we aimed to maintain the original data distribution that depicted a real-world scenario of our problem domain, to challenge it with our best-trained models.

To properly interpret the PR-AUC results in an unbalanced dataset, it is essential to recalculate the random classifier baseline using the method described in the literature, which is detailed in S3 Appendix. The baseline for textit ${\text{X}}_{\text{testing}}$ in Dataset 5 is approximately 0.0329. This value indicates that the expected accuracy of a random classifier on this dataset would be 3.29%, assuming all samples were classified randomly. Observations from Fig 12, which displays the PR curves of the tested SVM classifiers, provide insight into their performance. Some classifiers exhibit PR curves that remain relatively high across a substantial recall range, demonstrating their ability to maintain good accuracy while correctly identifying a higher proportion of positive cases. These classifiers are considered the best performers in this dataset.

Conversely, classifiers whose PR curves fall more steeply illustrate a rapid decrease in accuracy with increasing recall, indicating less robust performance. Despite this, the fact that the curves do not steeply drop to the baseline suggests that SVM classifiers, in general, exhibit decent performance, albeit with some variability. Among these, some classifiers achieve a good balance between accuracy and recall, making them suitable for broader application across various threshold scenarios. In contrast, others may be more finely tuned for specific conditions, which could limit their effectiveness in different contexts. These differences are captured in the width of the confidence intervals, reinforcing the need for cautious model selection based on the application scenario.

Our best classifier with SVM obtained a PRC-AUC of 0.4509 [95% CI: 0.4168–0.4864], which is significantly higher than the baseline of 0.0329. This suggests that our classifier outperforms a random model by a considerable margin. If the baseline were near 0.5, as in a balanced dataset like the one we created for the training and validation phase (with ROC and PR curves depicted in Fig 11), a PR-AUC of 0.4509 would be less remarkable. Nevertheless, given the very low baseline of 0.0329, a PR-AUC of 0.4509 signifies that our classifier is proficient at detecting positive samples, even within a highly unbalanced dataset. We can conclude that the classifier is doing a good job in distinguishing between positive and negative classes, especially considering the unbalanced proportion in the dataset. Although there is always room for improvement, a PRC-AUC of 0.4509 in this context shows that the classifier performs robustly and is much better than a randomized approach. Moreover, the tight confidence interval suggests that this performance is stable across testing subsets. It is expected that as we expand the distribution of workload footprints for injured soccer players (minority class) [113], the PR-AUC will improve while the Type I and Type II errors will decrease. Hence, we highlight the importance of creating a formal shared-global and anonymized database of workload footprints for injured soccer players, to enhance the reliability of machine learning models in an elite team setting.

With the aim of complement the evaluation based on the best single model performance, we also analyzed the statistical distribution of testing metrics across 20 independently trained models for each algorithm. Each of these models was evaluated on the same hold-out test set composed of unseen data. By computing the mean and the 95% confidence intervals (IC95%) for key evaluation metrics (ROC-AUC, G-Mean, Accuracy, PR-AUC, Type I Error, and Type II Error), we aimed to assess not only peak performance but also the consistency and reliability of each modeling approach. The resulting distributions allowed us to compute mean values and 95% confidence intervals (IC95%) using the t-Student method, providing robust estimates of generalization performance variability.

The analysis, summarized in Table 8, revealed that the Footballer Workload Footprint (FWF)-based datasets consistently achieved superior mean values across most metrics, accompanied by narrower confidence intervals compared to traditional ACWR-based methods. This indicates not only improved metrics of performance, but also lower variability, suggesting greater reliability across different samples. Specifically, the models trained using cumulative and temporary variations of the FWF showed substantial improvements in ROC-AUC, PR-AUC, and G-Mean scores, while simultaneously reducing both Type I and Type II errors. Importantly, the non-overlapping IC95% intervals between FWF-based and ACWR-based models across key metrics support that these improvements are unlikely due to random variation but reflect a true underlying advantage. Thus, from a scientific and practical standpoint, incorporating richer, temporally-structured representations of player workload, as enabled by the FWF matrices, appears to substantially enhance the predictability of non-contact injury risks in elite soccer environments. This structured temporal modeling, reinforced by statistical validation, provides a robust framework that bridges academic rigor with real-world applicability in injury prevention.

In addition to confirming the same conclusions from the training and validation process, results of testing phase proves that our feature engineering approach—the Calculation of Cumulative and Temporal Variations of an External Workload Footprint of a Soccer Player—representing in Dataset 5, is the best proposal in predicting non-contact muscle injuries in real competition scenarios, enhancing decision-making compared to relying solely on the calculation of ratios ‘${\text{ACWR}}_{UNCOUPLED}$’. To the best of our knowledge [20], our approach has achieved the best results, matching the performance of the most cited proposal in the current state of the art, which is Rossi’s [28]. It also happens, comparing with Colby et al. [44], Carey et al. [45], Vallance et al. [46] Hecksteden et al. and [47] proposals.

The papers by Rossi et al. and Carey et al. [28,45] both report the highest testing results with a ROC/AUC of 0.76. However, the study by Carey et al. [45] specifically addresses hamstring injuries in soccer players, whereas Rossi’s encompasses various types of non-contact injuries. Furthermore, Carey et al.’s study lacks a variety of metrics that would provide a more comprehensive assessment of prediction capabilities. Given the unbalanced nature of the dataset involved, it would be beneficial to incorporate metrics more aligned with the specific challenges posed by the data, such as G-Mean, Error Type I, and Error Type II. Rossi et al. [28] have more effectively addressed this aspect in their research. However, neither of the most cited papers presents an analysis of the Precision Recall curve or the PR-AUC metric, which could further enrich the evaluation of their predictive models in handling class imbalance. We encourage researchers in this field to publish their PR curves and PR-AUC metrics from now on. At this time then, comparing different proposals and studies in an objective and formal manner is challenging due to the absence of a standardized modeling approach. To further analyze this gap and objectively position our contribution, we present in Table 10 a structured comparison of the three approaches, including the number of models trained, whether the reported metrics correspond to unseen data, the application of confidence intervals, the validation strategy, and model interpretability.

**Table 10:** Comparison of methodologies and results between Carey et al. (2018), Rossi et al. (2018), and our study.

Aspect	Carey et al.	Rossi et al.	This work
Sport	Australian Football	Soccer (Calcio)	Soccer (LaLiga + UEFA)
Data	GPS, RPE	GPS	GPS
Sample size	46 players, 3 season	26 players, 23 weeks	23 players, 1 season
Feature Engineering	Daily loads, ACWR, monotony; no selection or transformation	55 load + biometric vars; 12 ACWR + 12 MSWR; RFECV	FWF matrices; cumulative/differential variations; 5 pipelines; PCA (95% VE)
Balancing Technique	SMOTE	ADASYN	Undersampling + SMOTE
Modeling Techniques	LogReg, SVM, RF, GEE	DT, LogReg, RF, NB, SVM, KNN	SVM, RF, MLP, CART, LDA, NB, KNN
Validation Strategy	Train 2 seasons, test 3rd	Rolling week-by-week retraining and forecasting	10-Repeated-Stratified-2-Fold + Independent Test
Best ROC-AUC	0.76 (hamstrings)	0.76 (general)	0.7707 (muscular)
PR-AUC Reported	No	No	0.4509
Bias-Variance Analysis	No	No	Yes
Permutation Test	No	No	Yes (p ≤ 0.001)
Number of Models Trained	12	6–7	160
Metrics on Unseen Data	Yes	Partial (rolling)	Yes
IC95% Reported on Testing Data	No	No	Yes
Model Interpretability	None reported	Rule-based trees with simple thresholds	Heatmaps of FWF, cumulative/temporal variation matrices, error decomp.

Carey et al. [45] highlighted the challenges of injury prediction with GPS-derived metrics, noting that even advanced machine learning methods struggled to exceed ROC-AUC values of 0.65 for most injury types, except for hamstring injuries where an AUC of 0.76 was achieved. Their methodology systematically trained models on two seasons of data and evaluated them on a third, thus reporting performance on truly unseen data, although without reporting formal confidence intervals on testing results and using a limited variety of evaluation metrics.

Rossi et al. [28] made a significant step forward by introducing a multidimensional feature set consisting of over 50 biomechanical and workload variables, including 12 ACWR and 12 MSWR-derived metrics. Their approach implemented a weekly rolling forecast with recursive feature elimination, but was constrained by a limited sample size, absence of dimensionality reduction, and no independent testing block.

In contrast, our work proposes a more rigorous and generalizable framework through the introduction of the Footballer Workload Footprint (FWF), which models training loads as discrete time series matrices and systematically computes cumulative and differential variations. Moreover, 160 models were trained across eight supervised multivariate techniques, ensuring a robust exploration of the predictive space. The application of advanced undersampling, oversampling (SMOTE), dimensionality reduction (PCA), repeated stratified cross-validation, permutation testing, and bias-variance tradeoff analysis provides a stronger empirical foundation for our results.

Our models, particularly those trained with FWF cumulative and temporary variations (Dataset 5), achieved a best ROC-AUC of 0.7707 [95% CI: 0.7407–0.8094] and a best PR-AUC of 0.4509 [95% CI: 0.4168–0.4864] when tested on truly unseen data from the most competitive phase of the season. Additionally, all reported performance metrics were accompanied by 95% confidence intervals, further reinforcing the robustness and statistical validity of our findings.

In terms of model interpretability, Carey et al. provided no explicit interpretative tools, whereas Rossi et al. emphasized the extraction of human-readable rules from decision trees. Our study advances this dimension by integrating visual heatmaps and structured variation matrices (FWF) that facilitate interpretability at the individual player level, aligning predictive outputs with intuitive representations usable by medical and technical staff.

Therefore, the FWF approach not only improves predictive accuracy but also enhances the explainability, robustness, and real-world applicability of injury forecasting models in elite soccer. This methodological advancement suggests a pathway toward the establishment of standardized, reproducible, and scalable frameworks for injury prediction across professional sports contexts.

Given the critical importance of player health and the growing competitive demands on soccer players throughout the season, it is crucial for official bodies such as FIFA and/or UEFA to consider allocating resources towards continued research in this area. Establishing a standardized modeling approach, as FWF matrix proposed in this paper, could facilitate the creation of an global-shared, anonymized and accessible workloads footprints injury database. Such a resource would enable medical teams, physical trainers, and sports data scientists to contribute data and access a centralized repository, thereby enhancing collective efforts in injury prevention and management.

Results are encouraging, as they underscores the validity and potential of our methodology within the context of advanced analytics in sports science, offering a robust alternative that could be implemented on elite soccer teams in the medium term, specially if data shared-banks are consolidated. Our approach not only improves injury prediction ability comparatively to the various ACWR calculations used by experts, but also could paves the way for a more detailed and refined analysis of risk factors, thus optimizing strategies for prevention and intelligent management of workloads for elite soccer players thanks to the FWF’s explanatory capabilities, as depicted in Figs 8 and 9. The effective implementation of this methodology could significantly contribute to improving athlete well-being and career longevity, as well as maximizing their performance in critical moments of competitions.

## 5 Conclusions and future work

In this work, we propose new ideas to model and control the training workload of players, aimed to forecast more effectively non-contact injuries in elite soccer teams, the day before a match. Acute:chronic workload ratio (ACWR) measurement is an accepted practice in elite soccer teams to prevent non-contact injuries and minimize risk, but as we have shown in our paper, it has important shortcomings to track monotony and temporal variations in load. Further, from a machine learning perspective, the inherently imbalanced nature of most collected datasets necessitates additional effort to properly handle the skewed class distribution.

To deal with all of that, we present a new approach to control training load inspired by bilinear modeling [39] and the theoretical foundations of signal processing [40]. Our method represents each external workload variable extracted from GPS data as a discrete time series (DTS), which are joined together in a temporal discrete matrix that we call the Footballer Workload Footprint (FWF). We also show how to calculate the cumulative and temporary variations of the ‘*FWF*_*V*× *E*_(*n*)_*f*_*k*__’ matrix, based on integral and differential calculus. This calculation can be considered the new calculation of ACWR in the era of machine learning era and, for this reason, we also proposed a look at explainability.

In order to evaluate our proposal, we designed an exploratory and confirmatory analysis which compared models trained with our new method, versus the models trained using different calculations of ACWR. We carried out it with the standard and most representative techniques of supervised machine learning, facing a real highly imbalanced and complete season dataset from a Spanish First Division (LaLiga) soccer elite team, that also compited on Copa del Rey and European UEFA competitions.

The conducted experiments supported significant improvements and insights. The results are coherent with existing literature, demonstrating that the predictive power of the different training load calculations, such as ‘${\text{ACWR}}_{COUPLED}$’ [7], ‘${\text{ACWR}}_{UNCOUPLED}$’ [8,12], and ‘${\text{ACWR}}_{EWMA}$’ [15,16] varies significantly and are not consistent enough to be relied upon in a real competitive environment to predict non contact injuries the day before the match, inline with Impellizeri’s et al. research [10]. However, the ‘*footprint*’-based models $\sum^{T}FWF$ and $\Delta^{T}FWF$ showed consistently superior performance during training/validation phase and in testing phase. During the testing phase with “unseen data”, spanning from mid-February to June, our top-performing model exhibited the following metrics: ROC-AUC = 0.7707 [95% CI: 0.7407–0.8094], G-Mean = 0.7701 [95% CI: 0.7412–0.7986], Accuracy = 0.7434 [95% CI: 0.7095–0.7758], PR-AUC = 0.4509 [95% CI: 0.4168–0.4864], Type I Error = 0.1999 [95% CI: 0.1792–0.2205], and Type II Error = 0.2585 [95% CI: 0.2139–0.2964]. The reported metrics reflect the performance of the top model on the test set, selected based on our evaluation criteria—maximizing ROC-AUC, G-Mean, and PR-AUC, while minimizing Type I and II errors. To account for variability, 95% confidence intervals were derived from the distribution of results across 20 independently trained models, providing a robust estimate of generalization performance. These results showed that, our approach improved the prediction performance with regard to the main state of the art accepted methods by sports science professionals. Indeed, to the best of our knowledge [20], our approach has achieved the best results, matching the performance of the most cited proposal in the current state of the art, which is Rossi’s [28]. It also happens, comparing with Colby et al. [44], Carey et al. [45], Vallance et al. [46] Hecksteden et al. and [47] proposals. The obtained results improved all the evaluation metrics: accuracy, type I error, type II error, G-mean, ROC/AUC, and PR/RC curves. The permutations test show that exists a real connection between the data modeled using the $\sum^{T}FWF$ and $\Delta^{T}FWF$ matrixes, and the class labels with a high level of significance (p−value≤0.001) and the bias-variance trade-off analysis performed to our best model showed the ability to capture the underlying relationship between the input and output variables.

The main weakness of the results obtained lies in the low PR-AUC, so the models have difficulties to correctly identify the instances of the positive class (the injured), especially in unbalanced datasets, as is the case of the real-world environment of our problem. However, neither most cited papers present an analysis of the Precision Recall curve or the PR-AUC metric, which could further enrich the evaluation of their predictive models in handling class imbalance. We encourage researchers in this field to publish their PR curves and PR-AUC metrics from now on, ideally accompanied by confidence intervals. Doing so would allow not only comparisons of central performance values, but also assessments of stability and statistical reliability, which are essential in high-stakes applications like injury prediction.

The main issue is not the lack of injury samples, as these will naturally become more abundant over time, due to elite clubs allocating more resources in this direction. Let us recall that the objective of physical trainers and medical teams is precisely to avoid the events of the positive class, i.e., that soccer players get injured. Rather, the key difficulty lies in the lack of an established industry standard for gathering, modeling, and processing players workloads and injury data for appropriate reuse in research and/or for creating global-shared, anonymized and public models that can be retrained and refined over time. Our paper illustrates that our proposed method, the Footballer Workload Footprint (FWF), is efficient, practical, and resilient in tackling these challenges.

Results are therefore encouraging, as they underscores the validity and potential of our methodology within the context of advanced analytics in sports science, offering a robust alternative that could be implemented on elite soccer teams in the medium term, specially if shared-data banks are consolidated. However, some limitations must be noted. The present study used data from a single elite Spanish First Division team (LaLiga) over one season, excluding goalkeepers, which may affect the generalizability of the results to other leagues, competitive contexts, or playing styles. Furthermore, variations in GPS technology, differences in internal workload monitoring practices, and non-standardized injury classification across clubs can introduce additional variability that may impact model transferability. Future studies should seek to validate the Footballer Workload Footprint (FWF) approach across multiple teams, seasons, and leagues, ideally using harmonized data collection protocols.

Building on the results obtained and addressing the limitations identified, we propose the following specific lines of future research:

1. *Access to new GPS Data from Elite Teams*: It is recommended to gain access to complete season GPS data from other elite teams. This will enable the application of the ‘*footprint*’ method across multiple datasets, thereby improving the model’s generalizability and its ability to predict injuries in different competitive contexts.
2. *Hyperparameter Optimization*: It is suggested to optimize hyperparameters at various stages of the methodology, including the model training phase, to enhance performance metrics. A meticulous hyperparameter tuning could enhance model performance metrics and its capacity to adapt to different data scenarios.
3. *Exploration of Oversampling Methods*: The utilization of various oversampling methods and the optimization of their hyperparameters should be explored to achieve better performance metrics. This is particularly relevant for addressing class imbalance and enhancing the model’s ability to accurately predict both injury events and non-events.
4. *Feature Selection and Dimensionality Reduction*: Implement additional feature selection and/or dimensionality reduction methods, such as dimensional reduction techniques or specific feature selection algorithms. Analyzing the impact on performance metrics will help identify the most relevant features and improve model interpretability.
5. *Applied Conformal Prediction and Classifier Calibration Probabilities*: The application of conformal prediction techniques and classifier calibration probabilities can significantly improve the accuracy and reliability of injury prediction models. Conformal prediction offers a statistical framework to generate valid prediction sets with a specified confidence level, allowing models to quantify the uncertainty associated with their predictions. Coupled with classifier calibration and utilize metrics such as Brier Score and Log Loss, can enhance the precision of predicted probabilities for injury risk. Accurate calibration ensures that the predicted probabilities align closely with actual outcomes, providing sports professionals with a more dependable basis for decision-making in injury prevention and athlete management.
6. *Use of Ensembles and Deep Learning*: Investigate the use of ensemble techniques and deep learning, focusing on improving metrics such as the area under the precision-recall curve (PR-AUC) and minimizing Type I and Type II errors. These advanced techniques could offer significant improvements in the prediction and management of injury risk.
7. *Exploration of Footprint Model Explainability and Interpretability*: Further investigate the explainability and interpretability of the ‘*footprint*’ model through new exploratory data analyses, both at the individual player level and by field position. This will allow a better understanding of the factors contributing to injuries and help develop more effective prevention strategies.
8. *Establishment of Comparative Metrics for Footprints*: Develop and validate a specific metric that allows for the objective comparison of different player’s FWF matrixes. This metric should be capable of capturing key differences in ‘*footprint*’ characteristics, thereby facilitating comparisons across different players, teams, and even, playing conditions. A robust and standardized metric will enhance the interpretation of results and help identify common or anomalous patterns that may be associated with a higher risk of injury.
9. *Categorizing Footprints and Associating Them with Injury Typology*: Implement a system for categorizing the ‘*footprint*’s of injured players to associate them with specific types of non-contact injuries. This analysis should investigate which variables have the greatest impact and significance for each type of injury. By identifying specific ‘*footprint*’ patterns associated with different types of injuries, more personalized strategies for injury prevention and management can be developed.
10. *Training of Specific Models*: Train specific models tailored to individual players and/or by field position. This model customization could enhance predictive accuracy and allow for more personalized interventions in injury prevention.
11. *Batch-wise Unfolding in Footprint Modeling*: Explore the possibility of modeling ‘*footprints*’ using a batch-wise unfolding approach instead of a variable-wise unfolding approach. Although the variable-wise patterns integrate time within them, batch-wise unfolding alternative method could provide a better representation of temporal dynamics in patterns and maybe improve the model’s predictive metrics.
12. *Incorporation of New Variables into the Footprint Model*: Expand the ‘*footprint*’ model to include new variables that may influence injury risk. For example:
  - *Internal Load Variables for the Player*: Indicators such as heart rate, heart rate variability, and other biomarkers reflecting the physiological response of the player to training and competition. These metrics can provide crucial insights into the player’s adaptation to workload.
  - *Nutritional Variables*: Data on macronutrient and micronutrient intake, hydration levels, and other dietary indicators that could affect performance and recovery. Adequate nutrition is fundamental for injury prevention and recovery, and its inclusion in the model could enhance predictive accuracy.
  - *Muscle Quality Variables*: Parameters such as lactate levels and other biomarkers of muscle fatigue and recovery. These indicators offer a more detailed view of the player’s muscular status and their capacity to handle workloads without an elevated risk of injury.
  - *Wellness-Related Variables*: Factors such as sleep quality, perceived stress levels, subjective recovery, and overall mental health. These variables can significantly influence injury risk and the player’s overall performance, and their inclusion could improve the model’s ability to predict non-contact injuries.

These strategies not only have the potential to improve current metrics but could also contribute new insights into injury prevention within the elite sports context. Furthermore, they may enhance the generalization and practical applicability of injury prediction models, helping to bridge the gap between scientific research and daily decision-making by sports professionals. Also will contribute to the development of more reliable and personalized injury prediction and prevention systems. But the implementation of these proposals will require ongoing interdisciplinary collaboration and expanded access to high-quality datasets, such as the one that was available to us in the present study.

## Supporting information

S1 Appendix List of single binary classification supervised algorithms trained. (PDF)

S2 Appendix Metrics for model evaluation. (PDF)

S3 Appendix ROC/PR curves for model evaluation. (PDF)

S4 Appendix Permutation test for model evaluation. (PDF)

S5 Appendix Bias-variance TradeOff analysis for model evaluation. (PDF)

S1 TableDetailed information and descriptive information of each ${\text{v}}_{\text{i}}$ extracted from GPS devices.(PDF)
