# Supplementary material for: Advanced feature engineering in Acute:Chronic Workload Ratio (ACWR) calculation for injury forecasting in elite soccer
Source: PLoS One. 2025 Jul 23;20(7):e0327960. doi: 10.1371/journal.pone.0327960 (PMC12286412; doi:10.1371/journal.pone.0327960)
Supplement: S1 Appendix — (PDF) [file pone.0327960.s001.pdf]

Advanced feature engineering in acute:chronic workload ratio (ACWR) calculation for injury forecasting in elite soccer.

Jaime B. Matas-Bustos[1,*], Antonio M. Mora-García[1], Moisés De Hoyo-Lora[2], Alejandro Nieto-Alarcón[3], and Francisco T. Gonzalez-Fernández[4].

1 Department of Signal Theory, Telematics and Communications, University of Granada, Granada, Spain
2 Department of Physical Education and Sports, University of Sevilla, Sevilla, Spain
3 Escuela Técnica Superior de Ingeniería Informática y Telecomunicaciones (ETSIIT), University of Granada, Granada, Spain
4 Department of Physical Education and Sports, University of Granada, Granada, Spain

* jmatasbustos@gmail.com

Supporting information

S1 Appendix - List of Single Binary Classification Supervised Algorithms Trained :
We choosed the following algorithms:

- **Linear Discriminant Analysis:** Linear Discriminant Analysis (LDA) [1] is a supervised algorithm used to classify data into different groups or categories. It finds the linear combination of input features that maximizes the separation between the classes, thereby reducing the dimensionality of the data while retaining most of its discriminatory information. LDA assumes that the data is normally distributed and that the covariance matrices of the different classes are equal. It is commonly used in machine learning, pattern recognition, and data mining applications [2, 3].

- **Linear Regression:** Linear Regression (LR) [4] is a statistical technique used to model the relationship between two or more variables. If this is a linear relationship, a change in one variable will mean a proportional change in the rest. These variables are known as the dependent variable and the independent ones. LR aims to find the best-fit line that represents the relationship between the variables by minimizing the sum of squared errors between the predicted values and the actual ones. Thus, LR can be used to predict values of the dependent variable for new values of the independent variables. It is a widely used technique in data analysis and forecasting [5].

- **Naïve Bayes:** The Naive Bayes (NB) classifier is a probabilistic classifier that utilizes the Bayes' Theorem [6]. It assumes a "naive" or strong independence between the variables of the samples to be classified. This classifier is often employed as a baseline for comparison due to its ability to produce competitive results in a short amount of computational time. Additionally, the Naive Bayes classifier performs well in supervised training stages. It is highly scalable, requiring a linear number of parameters that depend on the number of variables in the problem. These parameters consist of the mean and standard deviations of

the variables of each class. As a result, the Naive Bayes classifier only evaluates closed-form expressions and does not require iterative refinement, as most other methods do. It will be considered as a baseline method for comparisons.

- **K-Nearest Neighbors:** The kNN [7, 8] is an instance-based classifier that classifies unknown patterns by comparing them to labelled patterns using a distance measure, typically Euclidean or Minkowski distances. The algorithm works on the premise that patterns that are far apart from each other are less likely to belong to the same class than patterns that are close together. In its simplest form, the algorithm identifies the nearest neighbour in the pattern space for each unknown pattern and assigns it the same class as its nearest neighbour. However, a more robust approach is commonly used, where k neighbours are identified, and the unclassified pattern is assigned the class of the majority of these neighbours.

- **Support Vector Machines:** SVM [9, 10] is a statistical learning theory-based method used in classification problems. The algorithm searches for the optimal hyperplane that maximizes the margin between two classes. This hyperplane is defined by a subset of support vectors from the training set. SVM is a highly robust and reliable method, even in high-dimensional spaces and when the problem is not linearly separable. Its success has been demonstrated in various forecasting and classification problems [11–13].

- **Classification and Regression Trees:** The goal of CART [14] is to create a tree-like model that represents the decision-making process for predicting the outcome of a dependent variable from several independent ones. The tree is built by selecting the best variable to split the data into subsets that are as homogeneous as possible. The splitting process continues until the tree is fully grown or a stopping criterion is met. For regression problems, the CART model predicts the mean value of the dependent variable for each leaf node, while for classification problems, it predicts the class label of the majority of samples in the leaf node. CART is a flexible and powerful algorithm that can handle both continuous and categorical data, so it can be applied to a wide range of applications [15, 16].

- **Random Forest:** RF [17] is a machine learning algorithm that combines multiple decision trees to create a powerful ensemble model. Random Forest builds a large number of decision trees on randomly selected subsets of the training data and then combines their predictions to make the final decision. Each decision tree is constructed using a different subset of the data and a different subset of the features, resulting in a diverse set of trees that collectively have better accuracy and generalization than any individual tree. Random Forest is capable of handling both classification and regression problems, and is particularly useful in situations where the number of input features is large. It is a robust algorithm that is less prone to overfitting than other decision tree-based algorithms. It can also effectively handle missing data and noisy features. RF is one of the most successful techniques applied to a vast amount of problems in the literature [18–20].

- **Multilayer Perceptron:** The Multilayer Perceptron (MLP) [21] is an artificial neural network that is frequently utilized for classification or approximation/regression problems. It is an extension of the standard linear perceptron, utilizing multiple layers of nodes (neurons). Each neuron is composed of a linear combination of weighted inputs that are passed through a non-linear activation

function, producing its output. As a result, MLPs are capable of solving linearly inseparable problems [22]. The network is usually trained using supervised learning, with the back-propagation method proposed by Werbos [23], Parker [24], and Rumelhart et al. [25]. This technique updates the weights of the output layer neurons based on the obtained erroneous output, then updates the weights backwards up to the input layer. Typically, MLPs include two hidden layers, as studies [26, 27] have demonstrated that it is sufficient to create classifying regions of any kind.

References

[1] GJ McLachlan. "Discriminant analysis and statistical pattern recognition. Wiley-Interscience: Hoboken". In: (2004).

[2] Alok Sharma and Kuldip K Paliwal. "Linear discriminant analysis for the small sample size problem: an overview". In: *International Journal of Machine Learning and Cybernetics* 6 (2015), pp. 443–454.

[3] Yaqian Guo, Trevor Hastie, and Robert Tibshirani. "Regularized linear discriminant analysis and its application in microarrays". In: *Biostatistics* 8.1 (2007), pp. 86–100.

[4] Xin Yan and Xiaogang Su. *Linear regression analysis: theory and computing.* world scientific, 2009.

[5] Sanford Weisberg. *Applied linear regression.* Vol. 528. John Wiley & Sons, 2005.

[6] Stuart Russell and Peter Norvig. *Artificial Intelligence: A Modern Approach.* Vol. 3rd ed. Prentice Hall, 1995.

[7] D. Aha and D. Kibler. "Instance-based learning algorithms". In: *Machine Learning* 6 (1991), pp. 37–66.

[8] T.M. Mitchell. "Machine Learning". In: *McGraw-Hill Companies, Inc.* (1997).

[9] C. Cortes and V. Vapnik. "Support Vector Networks". In: *Machine Learning* 20 (1995), pp. 273–297.

[10] S. K. Shevade et al. "Improvements to the SMO Algorithm for SVM Regression". In: *IEEE Transactions on Neural Networks* 11.5 (2000), pp. 1188–1193.

[11] L. Cao. "Support vector machine experts for time series forecasting". In: *Neurocomputing* 51 (2003), pp. 321–339.

[12] Jae H. Min and Youngchan Lee. "Bankruptcy prediction using support vector machine with optimal choice of kernel function parameters". In: *Expert Systems with Applications* 28.4 (2005), pp. 603–614.

[13] C. Jari, L. Wen, and L. Kang. "Support vector machine classification for large data set via minimum enclosing ball clustering". In: *Neurocomputing* 71 (2008), pp. 611–619.

[14] Leo Breiman. *Classification and regression trees.* Routledge, 2017.

[15] Stephenie C Lemon et al. "Classification and regression tree analysis in public health: methodological review and comparison with logistic regression". In: *Annals of behavioral medicine* 26 (2003), pp. 172–181.

[16] Xin Ma. *Using classification and regression trees: A practical primer.* IAP, 2018.

[17] Adele Cutler, D Richard Cutler, and John R Stevens. “Random forests”. In: *Ensemble machine learning: Methods and applications* (2012), pp. 157–175.

[18] Xi Chen and Hemant Ishwaran. “Random forests for genomic data analysis”. In: *Genomics* 99.6 (2012), pp. 323–329.

[19] Robin Genuer et al. “Random forests for big data”. In: *Big Data Research* 9 (2017), pp. 28–46.

[20] Yanjun Qi. “Random forest for bioinformatics”. In: *Ensemble machine learning: Methods and applications.* Springer, 2012, pp. 307–323.

[21] B. Widrow and M.A. Lehr. “30 years of adaptive neural networks: Perceptron, madaline and backpropagation”. In: *Proc. of the IEEE* 78.9 (1990), pp. 1415–1440.

[22] J. Steinwender and S. Bitzer. “Multilayer Perceptrons”. In: *A discussion of The Algebraic Mind. Technical Report. University of Osnabrueck* (2003).

[23] P.J. Werbos. “Beyond regression: New tools for prediction and analysis in the behavioral sciences”. In: *Ph.D. Thesis, Harvard University* (1974).

[24] D.B. Parker. “Learning logic”. In: *Tech. Report TR-47, Centre for Computational Research in Economics and manegement Science, MIT* (1985).

[25] D.E. Rumelhart and D. Zipser. “Feature discovery by competitive learning”. In: *Cognitive Science* 9 (1985), pp. 75–112.

[26] R.P. Lippmann. “An introduction to computing with neural nets”. In: *ASSP Magazine* (1987), pp. 4–22.

[27] C.M. Bishop. “Neural Networks for Pattern Recognition”. In: *Clarendon Press. Oxford University Press Inc., New York.* (1996).
