# Supplementary material for: Advanced feature engineering in Acute:Chronic Workload Ratio (ACWR) calculation for injury forecasting in elite soccer
Source: PLoS One. 2025 Jul 23;20(7):e0327960. doi: 10.1371/journal.pone.0327960 (PMC12286412; doi:10.1371/journal.pone.0327960)
Supplement: S2 Appendix — (PDF) [file pone.0327960.s002.pdf]

Advanced feature engineering in acute:chronic workload ratio (ACWR) calculation for injury forecasting in elite soccer.

Jaime B. Matas-Bustos[1,*], Antonio M. Mora-García[1], Moisés De Hoyo-Lora[2], Alejandro Nieto-Alarcón[3], and Francisco T. Gonzalez-Fernández[4].

1 Department of Signal Theory, Telematics and Communications, University of Granada, Granada, Spain
2 Department of Physical Education and Sports, University of Sevilla, Sevilla, Spain
3 Escuela Técnica Superior de Ingeniería Informática y Telecomunicaciones (ETSIIT), University of Granada, Granada, Spain
4 Department of Physical Education and Sports, University of Granada, Granada, Spain

* jmatasbustos@gmail.com

Supporting information

S2 Appendix - Metrics for Model Evaluation :
The metrics employed to evaluate and compare models' performance are based in the confusion matrix. In the field of machine learning and specifically the problem of statistical classification, a confusion matrix, also known as error matrix, is a specific table, layout that allows visualization of the performance of an algorithm, typically a supervised learning one; in unsupervised learning it is usually called a matching matrix. Each row of the matrix represents the instances in an actual class while each column represents the instances in a predicted class, or vice versa – both variants are found in the literature. The name stems from the fact that it makes it easy to see whether the system is confusing two classes (i.e. commonly mislabeling one as another).
Different types of metrics can be calculated from the confusion matrix to help measure and compare the performance of a model from different perspectives, as we show in Figure 1 [1]. Below is the description and formula of the key metrics selected for evaluating the models in our research, along with their justification and relevance for predicting non-contact injuries in elite soccer players.

Fig 1. Key metrics derived from the confusion matrix. Source: Wikimedia commons.

- **ROC-AUC (Area under the ROC Curve):** The ROC-AUC measures a model's ability to distinguish between positive and negative classes. It represents the area under the Receiver Operating Characteristic (ROC) curve. True Positive Rate (Sensitivity): Measures the proportion of positive instances correctly classified as positive. False Positive Rate (Specificity): Measures the proportion of negative instances incorrectly classified as positive. ROC-AUC: The area under the ROC curve provides a consolidated measure of the model's performance in discerning between positive and negative classes. A higher value signifies improved overall performance.

- **Formula:**

$$\text{ROC-AUC} = \int_0^1 \text{True Positive Rate (TPR)} \, d(\text{False Positive Rate (FPR)})$$

- **G-Mean (Geometric Mean):** The G-Mean aims to strike a balance between sensitivity and specificity, making it particularly useful when dealing with imbalanced classes. It is calculated as the square root of the product of the true positive and true negative rates. In the context of injuries, the G-Mean evaluates the model's ability to balance sensitivity and specificity, being sensitive to both false positives and false negatives. A higher G-mean value indicates a good performance for the two classes and that a classifier is not biased toward any of the classes.

  - **Formula:**

$$\text{G-Mean} = \sqrt{\text{Sensitivity} \times \text{Specificity}} = \sqrt{\text{TPR} \times \text{TNR}} = \sqrt{\frac{\text{TP}}{\text{P}} \times \frac{\text{FP}}{\text{N}}}$$

- **Accuracy:** Accuracy measures the proportion of correct predictions relative to the total number of predictions made by the model. Accuracy measures the overall proportion of correct predictions, encompassing both true positives and true negatives. In the context of injuries, it assesses the model's general ability to accurately classify both players who sustain injuries and those who do not. We will use it as a baseline metric since counting only total hits, without distinguishing between positive or negative, is not a good metric to use in unbalanced sets, such as the one we face in our application context. The accuracy of the prediction model is misleading as it favours the majority class and rarely predicts the minority class. This means that the model could achieve high accuracy values by correctly predicting many players who do not get injured (majority class present) but failing to predict any injuries (minority class present) when they are going to occur.

  - **Formula:**

$$\text{Accuracy} = \frac{\text{True Positives} + \text{True Negatives}}{\text{Total Predictions}} = \frac{\text{TP} + \text{TN}}{\text{P} + \text{N}}$$

- **PR-AUC (Area under the Precision-Recall Curve):** The PR-AUC measures the area under the precision-recall curve and is especially valuable when dealing with imbalanced classes. Precision: Precision measures the proportion of positive predictions that are truly positive. In the context of injuries, precision would be the ratio of injury predictions that are genuinely injuries. Recall: Recall measures the proportion of positive instances correctly identified by the model. In the context of injuries, this would be the ratio of injured players correctly identified. PR-AUC: The area under the Precision-Recall curve provides a succinct measure of the model's performance, balancing precision and recall. A higher value indicates superior overall performance in the injury detection task.

  - **Formula:**

$$\text{PR-AUC} = \int_0^1 \text{Precision} \, d(\text{Recall})$$

- **Type I Error (False Positive Rate - FPR):** Represents the proportion of negative instances incorrectly classified as positive, providing insights into the model's specificity. The lower the value, the fewer false positives we have and,

consequently, higher and better specificity. In our context application, if we predict that a player will get injured but they end up playing without injury, it could cause them to miss an important game. Therefore, it is important to aim for a low value.

- **Formula:**

$$\text{Error Type I} = \frac{\text{False Positives}}{\text{Total Real Negatives}} = \frac{\text{FP}}{\text{N}}$$

- **Type II Error (False Negative Rate - FNR):** The proportion of positive instances incorrectly classified as negative is represented by this metric, which provides insights into the model's sensitivity. A lower value is better, as it indicates fewer false negatives and, consequently, higher and better sensitivity. In our application context, this metric measures whether we correctly predicted that a player would not get injured, but they ultimately did. Consequently our priority will be to minimize this metric by avoiding false negatives.

- **Formula:**

$$\text{Error Type II} = \frac{\text{False Negatives}}{\text{Total Real Positives}} = \frac{\text{FN}}{\text{P}}$$

In summary, these metrics provide various viewpoints on the model's performance in injury detection, taking into account factors such as precision, recall, discriminative ability, the balance between sensitivity and specificity, and the overall accuracy of predictions.

S3 Appendix - ROC/PR Curves for Model Evaluation :
The subsequent paragraph contains supporting information that is a direct excerpt from the article titled "*The Precision-Recall Plot Is More Informative than the ROC Plot When Evaluating Binary Classifiers on Imbalanced Datasets*", authored by Takaya Saito and Marc Rehmsmeier [2]:
"*The ROC plot shows the tradeoff between specificity (TNR = 1 - FPR) and sensitivity (TPR = 1 - FNR)[3]. It is model-wide because it shows pairs of specificity and sensitivity values calculated at all possible threshold scores. In ROC plots, classifiers with random performance show a straight diagonal line from (0, 0) to (1, 1)[3], and this line can be defined as the baseline of ROC. A ROC curve provides a single performance measure called the Area under the ROC curve (AUC) score. AUC is 0.5 for random and 1.0 for perfect classifiers[4]. AUC scores are convenient to compare the performances of multiple classifiers. The precision-recall (PRC) plot shows precision values for corresponding sensitivity (recall) values. Similar to the ROC plot, the PRC plot provides a model-wide evaluation. The AUC score of PRC, denoted as AUC (PRC), is likewise effective in multiple-classifier comparisons [5]. While the baseline is fixed with ROC, the baseline of PRC is determined by the ratio of positives (P) and negatives (N) as y = P / (P + N). For instance, we have y = 0.5 for a balanced class distribution, but y = 0.09 for an imbalanced class distribution in which the ratio of P:N is 1:10. Because of this moving baseline, AUC (PRC) also changes with the P:N ratio. For instance, the AUC (PRC) of random classifiers is 0.5 only for balanced class distributions, whereas it is P / (P + N) for the general case, including balanced and imbalanced distributions. In fact, the AUC (PRC) is identical to the y position of the PRC baseline.*"
An example of calculating the baseline on the Precision-Recall Curve (PRC) for X_{testing} for Dataset 5 could be delineated as follows. As indicated in Table **??**, the dataset comprises P = 5 positive samples and N = 147 negative samples, resulting in

a total of P+N = 152 samples. The baseline for the PRC, representing the proportion of positive samples in the dataset, would thus be calculated by the formula:

$$y = \frac{P}{P+N} = \frac{5}{5+147} = \frac{5}{152} \approx 0.0329$$

This baseline represents the maximum expected recall for a model that classifies all samples as positive, providing a point of reference against which the performance of predictive models can be assessed.

References

[1] *Template:Diagnostic testing diagram.* en. Page Version ID: 1159643334. June 2023. URL: https://en.wikipedia.org/w/index.php?title=Template:Diagnostic_testing_diagram&oldid=1159643334 (visited on 08/12/2023).

[2] Takaya Saito and Marc Rehmsmeier. "The Precision-Recall Plot Is More Informative than the ROC Plot When Evaluating Binary Classifiers on Imbalanced Datasets". In: *PLoS ONE* 10.3 (Mar. 2015), e0118432. ISSN: 1932-6203. DOI: 10.1371/journal.pone.0118432. URL: https://www.ncbi.nlm.nih.gov/pmc/articles/PMC4349800/ (visited on 08/15/2024).

[3] Tom Fawcett. "An introduction to ROC analysis". en. In: *Pattern Recognition Letters* 27.8 (June 2006), pp. 861–874. ISSN: 01678655. DOI: 10.1016/j.patrec.2005.10.010. URL: https://linkinghub.elsevier.com/retrieve/pii/S016786550500303X (visited on 10/17/2021).

[4] J. A. Hanley and B. J. McNeil. "The meaning and use of the area under a receiver operating characteristic (ROC) curve". eng. In: *Radiology* 143.1 (Apr. 1982), pp. 29–36. ISSN: 0033-8419. DOI: 10.1148/radiology.143.1.7063747.

[5] Jesse Davis and Mark Goadrich. "The relationship between Precision-Recall and ROC curves". In: *Proceedings of the 23rd international conference on Machine learning.* ICML '06. New York, NY, USA: Association for Computing Machinery, June 2006, pp. 233–240. ISBN: 978-1-59593-383-6. DOI: 10.1145/1143844.1143874. URL: https://doi.org/10.1145/1143844.1143874 (visited on 08/15/2024).
