# Supplementary material for: Advanced feature engineering in Acute:Chronic Workload Ratio (ACWR) calculation for injury forecasting in elite soccer
Source: PLoS One. 2025 Jul 23;20(7):e0327960. doi: 10.1371/journal.pone.0327960 (PMC12286412; doi:10.1371/journal.pone.0327960)
Supplement: S3 Appendix — (PDF) [file pone.0327960.s003.pdf]

Advanced feature engineering in acute:chronic workload ratio (ACWR) calculation for injury forecasting in elite soccer.

Jaime B. Matas-Bustos[1,*], Antonio M. Mora-García[1], Moisés De Hoyo-Lora[2], Alejandro Nieto-Alarcón[3], and Francisco T. Gonzalez-Fernández[4].

1 Department of Signal Theory, Telematics and Communications, University of Granada, Granada, Spain
2 Department of Physical Education and Sports, University of Sevilla, Sevilla, Spain
3 Escuela Técnica Superior de Ingeniería Informática y Telecomunicaciones (ETSIIT), University of Granada, Granada, Spain
4 Department of Physical Education and Sports, University of Granada, Granada, Spain

* jmatasbustos@gmail.com

Supporting information

S3 Appendix - ROC/PR Curves for Model Evaluation :
The subsequent paragraph contains supporting information that is a direct excerpt from the article titled "*The Precision-Recall Plot Is More Informative than the ROC Plot When Evaluating Binary Classifiers on Imbalanced Datasets*", authored by Takaya Saito and Marc Rehmsmeier [1]:
"*The ROC plot shows the tradeoff between specificity (TNR = 1 - FPR) and sensitivity (TPR = 1 - FNR)[2]. It is model-wide because it shows pairs of specificity and sensitivity values calculated at all possible threshold scores. In ROC plots, classifiers with random performance show a straight diagonal line from (0, 0) to (1, 1)[2], and this line can be defined as the baseline of ROC. A ROC curve provides a single performance measure called the Area under the ROC curve (AUC) score. AUC is 0.5 for random and 1.0 for perfect classifiers[3]. AUC scores are convenient to compare the performances of multiple classifiers. The precision-recall (PRC) plot shows precision values for corresponding sensitivity (recall) values. Similar to the ROC plot, the PRC plot provides a model-wide evaluation. The AUC score of PRC, denoted as AUC (PRC), is likewise effective in multiple-classifier comparisons [4]. While the baseline is fixed with ROC, the baseline of PRC is determined by the ratio of positives (P) and negatives (N) as y = P / (P + N). For instance, we have y = 0.5 for a balanced class distribution, but y = 0.09 for an imbalanced class distribution in which the ratio of P:N is 1:10. Because of this moving baseline, AUC (PRC) also changes with the P:N ratio. For instance, the AUC (PRC) of random classifiers is 0.5 only for balanced class distributions, whereas it is P / (P + N) for the general case, including balanced and imbalanced distributions. In fact, the AUC (PRC) is identical to the y position of the PRC baseline.*"
An example of calculating the baseline on the Precision-Recall Curve (PRC) for X_{testing} for Dataset 5 could be delineated as follows. As indicated in Table **??**, the dataset comprises P = 5 positive samples and N = 147 negative samples, resulting in a total of P+N = 152 samples. The baseline for the PRC, representing the proportion

of positive samples in the dataset, would thus be calculated by the formula:

$$y = \frac{P}{P+N} = \frac{5}{5+147} = \frac{5}{152} \approx 0.0329$$

This baseline represents the maximum expected recall for a model that classifies all samples as positive, providing a point of reference against which the performance of predictive models can be assessed.

References

[1] Takaya Saito and Marc Rehmsmeier. "The Precision-Recall Plot Is More Informative than the ROC Plot When Evaluating Binary Classifiers on Imbalanced Datasets". In: *PLoS ONE* 10.3 (Mar. 2015), e0118432. ISSN: 1932-6203. DOI: 10.1371/journal.pone.0118432. URL: https://www.ncbi.nlm.nih.gov/pmc/articles/PMC4349800/ (visited on 08/15/2024).

[2] Tom Fawcett. "An introduction to ROC analysis". en. In: *Pattern Recognition Letters* 27.8 (June 2006), pp. 861–874. ISSN: 01678655. DOI: 10.1016/j.patrec.2005.10.010. URL: https://linkinghub.elsevier.com/retrieve/pii/S016786550500303X (visited on 10/17/2021).

[3] J. A. Hanley and B. J. McNeil. "The meaning and use of the area under a receiver operating characteristic (ROC) curve". eng. In: *Radiology* 143.1 (Apr. 1982), pp. 29–36. ISSN: 0033-8419. DOI: 10.1148/radiology.143.1.7063747.

[4] Jesse Davis and Mark Goadrich. "The relationship between Precision-Recall and ROC curves". In: *Proceedings of the 23rd international conference on Machine learning*. ICML '06. New York, NY, USA: Association for Computing Machinery, June 2006, pp. 233–240. ISBN: 978-1-59593-383-6. DOI: 10.1145/1143844.1143874. URL: https://doi.org/10.1145/1143844.1143874 (visited on 08/15/2024).
