# Supplementary material for: Advanced feature engineering in Acute:Chronic Workload Ratio (ACWR) calculation for injury forecasting in elite soccer
Source: PLoS One. 2025 Jul 23;20(7):e0327960. doi: 10.1371/journal.pone.0327960 (PMC12286412; doi:10.1371/journal.pone.0327960)
Supplement: S4 Appendix — (PDF) [file pone.0327960.s004.pdf]

Advanced feature engineering in acute:chronic workload ratio (ACWR) calculation for injury forecasting in elite soccer.

Jaime B. Matas-Bustos[1,*], Antonio M. Mora-García[1], Moisés De Hoyo-Lora[2], Alejandro Nieto-Alarcón[3], and Francisco T. Gonzalez-Fernández[4].

1 Department of Signal Theory, Telematics and Communications, University of Granada, Granada, Spain
2 Department of Physical Education and Sports, University of Sevilla, Sevilla, Spain
3 Escuela Técnica Superior de Ingeniería Informática y Telecomunicaciones (ETSIIT), University of Granada, Granada, Spain
4 Department of Physical Education and Sports, University of Granada, Granada, Spain

* jmatasbustos@gmail.com

Supporting information

S4 Appendix - Permutation Test for Model Evaluation :
To assess the dependence between features and targets in a dataset and to determine the ability of the estimator to exploit this relationship to make effective predictions, a permutation testing approach is employed. This method consists of permuting the targets to generate "random data" and calculating the empirical p-value against the null hypothesis that the features and targets are independent.
Any permutation test follows the following scheme:

1. **Permutation of Targets**: Targets are randomly permuted, decoupling them from the features with which they were originally associated. This creates a new data distribution where any observed correlation between features and targets should be the product of chance.

2. **Estimator Evaluation**: Model performance metrics (such as accuracy, ROC-AUC, PRC-AUC etc.) are recalculated using the permuted targets. This process is repeated multiple times (e.g., 1,000 or 10,000 permutations) to construct a distribution of the performance metric under the null hypothesis.

3. **Calculation of the p-value**: The p-value is calculated as the proportion of times the performance metric calculated from the permuted data equals or exceeds the metric observed in the original data. A low p-value indicates that such a high performance metric is unlikely to be obtained by chance, suggesting a significant dependence between features and targets.

The interpretation of the p-value is as follows:

- **Small p-value**: A low p-value (commonly less than 0.05) suggests that there is a real dependence between the characteristics and the targets. This implies that the estimator is effectively using this dependence to make accurate predictions.

- **Large p-value**: A high p-value may indicate that there is no real dependence between the features and the targets, or that the estimator has failed to take advantage of the existing dependence to improve predictions. This may be an indication that the model is not adequate or that improvement in feature selection or transformation is required.

Specifically, in our study, we employed label permutation Test 1 and calculated the p-error, as outlined in Definition 1 of the paper titled "*Permutation Tests for Studying Classifier Performance*" by Markus Ojala and Gemma C. Garriga[1]: "*A significant classifier for Test 1, that is, obtaining a small p-value, rejects the null hypothesis that the features and the labels are independent, meaning that there is no difference between the classes. Let us now study this by considering the following case analysis. If the original data contains a real (i.e., not a random effect) dependency between data points and labels, then: (1) a significant classifier f will use such information to achieve a good classification accuracy and this will result in a small p-value (because the randomized samples do not contain such dependency. (2) if the classifier f is not significant in the sense of Test 1 (that is, f was not able to use the existing dependency between data and labels in the original data), then the p-value would tend to be high because the error in the randomized data will be similar to the error obtained in the original data. Finally, if the original data did not contain any real dependency between data points and labels, that is, such dependency was similar to randomized datasets, then all classifiers tend to have a high p-value.*"

References

[1] Markus Ojala and Gemma C. Garriga. "Permutation Tests for Studying Classifier Performance". In: *The Journal of Machine Learning Research* 11 (2010), pp. 1833–1863. ISSN: 1532-4435.
