# Supplementary material for: Advanced feature engineering in Acute:Chronic Workload Ratio (ACWR) calculation for injury forecasting in elite soccer
Source: PLoS One. 2025 Jul 23;20(7):e0327960. doi: 10.1371/journal.pone.0327960 (PMC12286412; doi:10.1371/journal.pone.0327960)
Supplement: S5 Appendix — (PDF) [file pone.0327960.s005.pdf]

Advanced feature engineering in acute:chronic workload ratio (ACWR) calculation for injury forecasting in elite soccer.

Jaime B. Matas-Bustos[1,*], Antonio M. Mora-García[1], Moisés De Hoyo-Lora[2], Alejandro Nieto-Alarcón[3], and Francisco T. Gonzalez-Fernández[4].

1 Department of Signal Theory, Telematics and Communications, University of Granada, Granada, Spain
2 Department of Physical Education and Sports, University of Sevilla, Sevilla, Spain
3 Escuela Técnica Superior de Ingeniería Informática y Telecomunicaciones (ETSIIT), University of Granada, Granada, Spain
4 Department of Physical Education and Sports, University of Granada, Granada, Spain

* jmatasbustos@gmail.com

Supporting information

S5 Appendix - Bias-Variance TradeOff Analysis for Model Evaluation :
The analysis of the Bias-Variance TradeOff is an essential method for assessing and comprehending the performance of machine learning models. This approach breaks down a model's error into two primary components: bias and variance. [1]
Bias represents the difference between the model's average predictions and the true values; that is, it measures the model's ability to capture the underlying relationship between the input variables and the output. A high bias indicates a model that is too simplified and fails to capture the complexity of the phenomenon, resulting in systematic error or underfitting. [1]
On the other hand, variance quantifies the sensitivity of the model to fluctuations in the training data. A model with high variance may overfit the training data, capturing not only the true patterns but also random noise, leading to overfitting. An effective Bias-Variance TradeOff analysis seeks to identify an optimal equilibrium point where both components, bias and variance, are jointly minimized to reduce the overall model error. [1]
This analysis is crucial for model selection and fitting in machine learning problems, as it provides guidance for tuning model hyperparameters, selecting relevant features, and improving model generalization on unobserved data. Ultimately, a proper balance between bias and variance is essential for developing robust and accurate models that are able to perform well in a variety of data scenarios. [2]

References

[1] Thomas G Dietterich and Eun Bae Kong. "Machine Learning Bias, Statistical Bias, and Statistical Variance of Decision Tree Algorithms". en. In: ().

[2] Jerome H. Friedman. "On Bias, Variance, 0/1—Loss, and the Curse-of-Dimensionality". en. In: *Data Mining and Knowledge Discovery* 1.1 (Mar. 1997), pp. 55–77. ISSN: 1573-756X. DOI: 10.1023/A:1009778005914. URL: https://doi.org/10.1023/A:1009778005914 (visited on 08/27/2024).
