# Supplementary material for: Advanced feature engineering in Acute:Chronic Workload Ratio (ACWR) calculation for injury forecasting in elite soccer
Source: PLoS One. 2025 Jul 23;20(7):e0327960. doi: 10.1371/journal.pone.0327960 (PMC12286412; doi:10.1371/journal.pone.0327960)
Supplement: S1 Table — (PDF) [file pone.0327960.s006.pdf]

Advanced feature engineering in acute:chronic workload ratio (ACWR) calculation for injury forecasting in elite soccer.

Jaime B. Matas-Bustos[1,*], Antonio M. Mora-García[1], Moisés De Hoyo-Lora[2], Alejandro Nieto-Alarcón[3], and Francisco T. Gonzalez-Fernández[4].

1 Department of Signal Theory, Telematics and Communications, University of Granada, Granada, Spain
2 Department of Physical Education and Sports, University of Sevilla, Sevilla, Spain
3 Escuela Técnica Superior de Ingeniería Informática y Telecomunicaciones (ETSIIT), University of Granada, Granada, Spain
4 Department of Physical Education and Sports, University of Granada, Granada, Spain

* jmatasbustos@gmail.com

Supporting information

S1 Table. Detailed information and descriptive information of each v_i extracted from GPS devices.

Table 1. Detailed information and descriptive information of each v_i extracted from GPS devices.

Category	Variable Name (Units)	Description	Datatype
(1)	Total Time (min)	The total time elapsed since the start of the match or training session.	int
(1)	Hours (n)	The number of hours elapsed since the start of the match or training session.	int
(1)	Minutes (n)	The number of minutes elapsed since the start of the match or training session.	int
(2)	Distance (m)	The total distance covered by the player during the training session or match, measured in meters.	float
(3)	MAX Speed (km/h)	The maximum speed reached during the training session or match, measured in kilometers per hour.	float
(3)	[75-85]% (Cnt)	The number of times that the player reaches a speed between 75% and 85% of their maximum speed.	float
(3)	[85-95]% (Cnt)	The number of times that the player reaches a speed between 85% and 95% of their maximum speed.	float
(3)	[95-100]% (Cnt)	The number of times that the player reaches a speed between 95% and 100% of their maximum speed.	float
(3)	[0,6]km/h (Cnt)	The number of times that the player reaches a speed between 0 and 6 kilometers per hour, during the match or training session.	float
(3)	[6,12]km/h (Cnt)	The number of times that the player reaches a speed between 6 and 12 kilometers per hour, during the match or training session.	float
(3)	[12,18]km/h (Cnt)	The number of times that the player reaches a speed between 12 and 18 kilometers per hour, during the match or training session.	float
(3)	[18,21]km/h (Cnt)	The number of times that the player reaches a speed between 18 and 21 kilometers per hour, during the match or training session.	float
(3)	[21,24]km/h (Cnt)	The number of times that the player reaches a speed between 21 and 24 kilometers per hour, during the match or training session.	float
(3)	[24,50]km/h (Cnt)	The number of times that the player reaches a speed between 24 and 50 kilometers per hour, during the match or training session.	float
(4)	MAX Acc (m/s^2)	The maximum acceleration of the player during the training session or match, measured in meters per second squared.	float
(4)	MAX Dec (m/s^2)	The maximum deceleration of the player during the training session or match, measured in meters per second squared.	float
(4)	[70-80]% (Cnt)	The number of times the player reaches a range of 70 and 80 percent of the player's maximum acceleration achieved during the match or training session.	float
(4)	[80-90]% (Cnt)	The number of times the player reaches a range of 80 and 90 percent of the player's maximum acceleration achieved during the match or training session.	float
(4)	[90-100]% (Cnt)	The number of times the player reaches a range of 90 and 100 percent of the player's maximum acceleration achieved during the match or training session.	float
(4)	[-80,-70]% (Cnt)	The number of times the player reaches a range of -80 and -70 percent of the player's maximum deceleration achieved during the match or training session.	float
(4)	[-90,-80]% (Cnt)	The number of times the player reaches a range of -90 and -80 percent of the player's maximum deceleration achieved during the match or training session	float
(4)	[-100, -90]% (Cnt)	The number of times the player reaches a range of -100 and -90 percent of the player's maximum deceleration achieved during the match or training session.	float
(6)	Player Load (Arb)	The total load placed on the player during the match or training session, measured in arbitrary units.	float
(6)	HMLD (Cnt)	The number of times the player reaches HMLD intensity during the match or training session.	float
(7)	[25,5 - 35]w/kg (Cnt)	The number of times the player reaches a power output of 25.5-35 watts per kilogram of body weight during the match or training session.	float
(7)	[35 - 55]w/kg (Cnt)	The number of times the player reaches a power output of 35-55 watts per kilogram of body weight during the match or training session.	float
(7)	[55 - 100]w/kg (Cnt)	The number of times the player reaches a power output of 55-100 watts per kilogram of body weight during the match or training session.	float
(8)	[8-100]G (Cnt)	The number of impacts with a force of 8-100 G s experienced by the player. It is measured in counts.	float
(8)	Step Balance(%)	The balance of the player while running, measured as a percentage of steps taken with the left and right foot.	float
(8)	Jumps count (Cnt)	The number of jumps made by the player during the match or training session.	float
(2) (3)	[75-85]% (m)	The percentage of distance covered by the player during the match or training session at a speed between 75% and 85% of their maximum speed, measured in meters.	float
(2) (3)	[85-95]% (m)	The percentage of distance covered by the player during the match or training session at a speed between 85% and 95% of their maximum speed, measured in meters.	float
(2) (3)	[95-100]% (m)	The percentage of distance covered by the player during the match or training session at a speed between 95% and 100% of their maximum speed, measured in meters.	float
(2) (3)	[0-6]km/h (m)	The distance covered by the player during the match or training session at a speed between 0 and 6 kilometers per hour, measured in meters.	float
(2) (3)	[6-12]km/h (m)	The distance covered by the player during the match or training session at a speed between 6 and 12 kilometers per hour, measured in meters.	float
(2) (3)	[12-18]km/h (m)	The distance covered by the player during the match or training session at a speed between 12 and 18 kilometers per hour, measured in meters.	float
(2) (3)	[18-21]km/h (m)	The distance covered by the player during the match or training session at a speed between 18 and 21 kilometers per hour, measured in meters.	float
(2) (3)	[21-24]km/h (m)	The distance covered by the player during the match or training session at a speed between 21 and 24 kilometers per hour, measured in meters.	float
(2) (3)	[24-50]km/h (m)	The distance covered by the player during the match or training session at a speed between 24 and 50 kilometers per hour, measured in meters.	float
(2) (4)	[0-1] m/s^2 (m)	The distance covered by the player during the match or training session while accelerating at a rate of 0 to 1 meters per second squared, measured in meters.	float
(2) (4)	[1-2] m/s^2 (m)	The distance covered by the player during the match or training session while accelerating at a rate of 1 to 2 meters per second squared.	float
(2) (4)	[2-3]m/s^2 (m)	The distance covered by the player during the match or training session while accelerating at a rate of 2 to 3 meters per second squared, measured in meters.	float
(2) (4)	[3-4]m/s^2 (m)	The distance covered by the player during the match or training session while accelerating at a rate of 3 to 4 meters per second squared, measured in meters.	float
(2) (4)	[4-5]m/s^2 (m)	The distance covered by the player during the match or training session while accelerating at a rate of 4 to 5 meters per second squared, measured in meters.	float
(2) (4)	[5-6]m/s^2 (m)	The distance covered by the player during the match or training session while accelerating at a rate of 5 to 6 meters per second squared, measured in meters.	float
(2) (4)	[6-10]m/s^2 (m)	The distance covered by the player during the match or training session while accelerating at a rate of 6 to 10 meters per second squared, measured in meters.	float
(2) (4)	[-1,0]m/s^2 (m)	The distance covered by the player during the match or training session while decelerating at a rate of 0 to -1 meters per second squared, measured in meters.	float
(2) (4)	[-2,-1]m/s^2 (m)	The distance covered by the player during the match or training session while decelerating at a rate of -1 to -2 meters per second squared, measured in meters.	float
(2) (4)	[-3,-2]m/s^2 (m)	The distance covered by the player during the match or training session while decelerating at a rate of -2 to -3 meters per second squared, measured in meters.	float
(2) (4)	[-4,-3]m/s^2 (m)	The distance covered by the player during the match or training session while decelerating at a rate of -3 to -4 meters per second squared, measured in meters.	float
(2) (4)	[-5,-4]m/s^2 (m)	The distance covered by the player during the match or training session while decelerating at a rate of -4 to -5 meters per second squared, measured in meters.	float
(2) (4)	[-6,-5]m/s^2 (m)	The distance covered by the player during the match or training session while decelerating at a rate of -5 to -6 meters per second squared, measured in meters.	float
(2) (4)	[-10,-6]m/s^2 (m)	The distance covered by the player during the match or training session while decelerating at a rate of -6 to -10 meters per second squared, measured in meters.	float
(2) (4)	[70-80]% (m)	The total distance covered by the player in a range of 70 and 80 percent of the player's maximum acceleration achieved during the match or training session, measured in meters.	float
(2) (4)	[80-90]% (m)	The total distance covered by the player in a range of 80 and 90 percent of the player's maximum acceleration achieved during the match or training session, measured in meters.	float
(2) (4)	[90-100]% (m)	The total distance covered by the player in a range of 90 and 100 percent of the player's maximum acceleration achieved during the match or training session, measured in meters.	float
(2) (4)	[-80,-70]% (m)	The total distance covered by the player in a range of -80 and -70 percent of the player's maximum deceleration achieved during the match or training session, measured in meters.	float
(2) (4)	[-90,-80]% (m)	The total distance covered by the player in a range of -90 and -80 percent of the player's maximum deceleration achieved during the match or training session, measured in meters.	float
(2) (4)	[-100,-90]% (m)	The total distance covered by the player in a range of -100 and -90 percent of the player's maximum deceleration achieved during the match or training session, measured in meters.	float
(2) (6)	HMLD (m)	The total distance covered by the player during the match or training session while moving at high metabolic load (HMLD) intensity, measured in meters.	float
(2) (7)	[25,5 - 35]w/kg (m)	The total distance covered by the player during the match or training session while moving at a power output of 25.5-35 watts per kilogram of body weight, measured in meters.	float
(2) (7)	[35 - 55]w/kg (m)	The total distance covered by the player during the match or training session while moving at a power output of 35-55 watts per kilogram of body weight, measured in meters.	float
(2) (7)	[55 - 100]w/kg (m)	The total distance covered by the player during the match or training session while moving at a power output of 55-100 watts per kilogram of body weight, measured in meters.	float
(2) (3) (9)	D-12-21 km/h (m)	The total distance covered by the player during the match or training session at a speed range of 12-21 km/h, measured in meters.	float
(2) (3) (9)	D-18-21 km/h (m)	The total distance covered by the player during the match or training session at a speed range of 18-21 km/h, measured in meters.	float
(2) (3) (9)	D-18-24 km/h (m)	The total distance covered by the player during the match or training session at a speed range of 18-24 km/h, measured in meters.	float
(2) (3) (9)	D-21-24 km/h (m)	The total distance covered by the player during the match or training session at a speed range of 21-24 km/h, measured in meters.	float
(2) (3) (9)	D->24 km/h (m)	The total distance covered by the player during the match or training session at a speed range greater than 24 km/h, measured in meters.	float
(2) (3) (9)	SP 75-85% (m)	The total distance covered by the player at a speed between 75-85% of the player's maximum speed during the match or training session.	float
(2) (3) (9)	D>85% (m)	The total distance covered by the player at a speed greater than 85% of the player's maximum speed during the match or training session.	float
(2) (4) (9)	D-ACC>3 (m)	The total distance covered by the player during periods of high acceleration (> +3 m/s^2) during the match or training session.	float
(2) (4) (9)	D-DEC>3 (m)	The total distance covered by the player during periods of high deceleration (> -3 m/s^2) during the match or training session.	float
(2) (4) (9)	D-ACC>80% (m)	The total distance covered by the player during periods of high acceleration (>80% of the player's maximum acceleration) during the match or training session.	float
(2) (4) (9)	D-DEC>80% (m)	The total distance covered by the player during periods of high deceleration (>80% of the player's maximum deceleration) during the match or training session.	float
(3) (5) (9)	18-21 (n)	The number of sprints made by the player at speed range of 18-21 km/h during the match or training session.	float
(3) (5) (9)	21-24 (n)	The number of sprints made by the player at speed range of 21-24 km/h during the match or training session.	float
(3) (5) (9)	18-24 (n)	The number of sprints made by the player at speed range of 18-24 km/h during the match or training session.	float
(3) (5) (9)	>24 (n)	The number of sprints made by the player at speed range greater than 24 km/h during the match or training session.	float
(3) (5) (9)	SP 75-85% (n)	The number of sprints made by the player at a speed between 75-85% of the player's maximum speed during the match or training session.	float
(3) (5) (9)	D>85% (n)	The number of sprints made by the player at a speed greater than 85% of the player's maximum speed during the match or training session.	float
(3) (5) (9)	D 12-21 (n)	The number of sprints made by the player at speed range of 12-21 km/h during the match or training session.	float
(4) (5) (9)	D-ACC>3 (n)	The number of sprints made by the player during periods of high acceleration (> +3 m/s^2) during the match or training session.	float
(4) (5) (9)	D-DEC>3 (n)	The number of sprints made by the player during periods of high deceleration (> -3 m/s^2) during the match or training session.	float
(4) (5) (9)	D-ACC>80% (n)	The number of sprints made by the player during periods of high acceleration (>80% of the player's maximum acceleration) during the match or training session.	float
(4) (5) (9)	D-DEC>80% (n)	The number of sprints made by the player during periods of high deceleration (>80% of the player's maximum deceleration) during the match or training session.	float
(1) (2) (10)	D Total (m/min)	Total distance per minute covered by the player during the match or training session.	float
(1) (6) (10)	HLMD (n/min)	Number of high metabolic load events per minute.	float
(1) (2) (3) (10)	D 12-21 Km/h (m/min)	Distance covered at a speed of 12 to 21 km/h in meters per minute of the match or training session.	float
(1) (2) (3) (10)	D 18-24 (m/min)	Distance covered at a speed of 18 to 24 km/h in meters per minute of the match or training session.	float
(1) (2) (3) (10)	D 21-24 (m/min)	Distance covered at a speed of 21 to 24 km/h in meters per minute of the match or training session.	float
(1) (2) (3) (10)	D>24 (m/min)	Distance covered at a speed greater than 24 km/h in meters per minute of the match or training session.	float
(1) (2) (3) (10)	SP 75-85% (m/min)	Distance covered in meters at a speed between 75-85% of maximal sprinting speed.	float
(1) (2) (3) (10)	D>85% (m/min)	Distance covered in meters at a speed greater than 85% of maximal sprinting speed per minute.	float
(1) (2) (4) (10)	ACC>3 (m/min)	The total distance covered by the player during periods of high acceleration (> +3 m/s^2)in meters per minute of the match or training session.	float
(1) (2) (4) (10)	ACC>80% (m/min)	The total distance covered by the player during periods of high acceleration (>80% of the player's maximum acceleration) in meters per minute of the match or training session.	float
(1) (2) (4) (10)	DEC>3 (m/min)	The total distance covered by the player during periods of high deceleration (> -3 m/s^2) in meters per minute of the match or training session.	float
(1) (2) (4) (10)	DEC>80% (m/min)	The total distance covered by the player during periods of high deceleration (>80% of the player's maximum deceleration) in meters per minute of the match or training session.	float
(1) (2) (6) (10)	HLMD (m/min)	High metabolic load distance covered in meters per minute.	float
(1) (3) (5) (10)	D 12-21 (n/min)	Number of sprints performed at a speed of 12 to 21 km/h per minute of the match or training session.	float
(1) (3) (5) (10)	18-24 (n/min)	Number of sprints performed at a speed of 18 to 24 km/h per minute of the match or training session.	float
(1) (3) (5) (10)	21-24 (n/min)	Number of sprints performed at a speed of 21 to 24 km/h per minute of the match or training session.	float
(1) (3) (5) (10)	D>24 (n/min)	Number of sprints performed at a speed greater than 24 km/h per minute of the match or training session.	float
(1) (3) (5) (10)	SP 75-85% (n/min)	Number of sprints performed at a speed between 75-85% of maximal sprinting speed per minute.	float
(1) (3) (5) (10)	D>85% (n/min)	Number of sprints performed at a speed greater than 85% of maximal sprinting speed per minute.	float
(1) (4) (5) (10)	ACC>3 (n/min)	The number of sprints made by the player during periods of high acceleration (> +3 m/s^2)in meters per minute of the match or training session.	float
(1) (4) (5) (10)	ACC>80%(n/min)	The number of sprints made by the player during periods of high acceleration (>80% of the player's maximum acceleration) in meters per minute of the match or training session.	float
(1) (4) (5) (10)	DEC>3 (n/min)	The number of sprints made by the player during periods of high deceleration (> -3 m/s^2) in meters per minute of the match or training session.	float
(1) (4) (5) (10)	DEC>80%(n/min)	The number of sprints made by the player during periods of high deceleration (>80% of the player's maximum deceleration) in meters per minute of the match or training session.	float
